# A New, Convenient Way to Fully Substituted α,β-Unsaturated γ-Hydroxy Butyrolactams

**DOI:** 10.3390/ijms241210213

**Published:** 2023-06-16

**Authors:** Alexander V. Aksenov, Dmitrii A. Aksenov, Igor A. Kurenkov, Alexander V. Leontiev, Nicolai A. Aksenov

**Affiliations:** Department of Chemistry, North Caucasus Federal University, 1a Pushkin St., 355009 Stavropol, Russia; daksenov@ncfu.ru (D.A.A.); kurenkman@icloud.com (I.A.K.); alvlleontev@ncfu.ru (A.V.L.); naksenov@ncfu.ru (N.A.A.)

**Keywords:** chalcones, cascade transformations, 5-hydroxy-3-pyrrolin-2-ones

## Abstract

The synthesis of novel, highly functionalized 5-hydroxy 3-pyrrolin-2-ones via a two-step procedure involving an addition reaction between KCN and corresponding chalcones, followed by ring condensation of the obtained β-cyano ketones with het(aryl)aldehydes under basic conditions is described. This protocol enables the preparation of various 3,5-di-aryl/heteroaryl-4-benzyl substituted α,β-unsaturated γ-hydroxy butyrolactams, which are subjects of significant interest to synthetic organic and medicinal chemistry.

## 1. Introduction

**Scheme 1:**
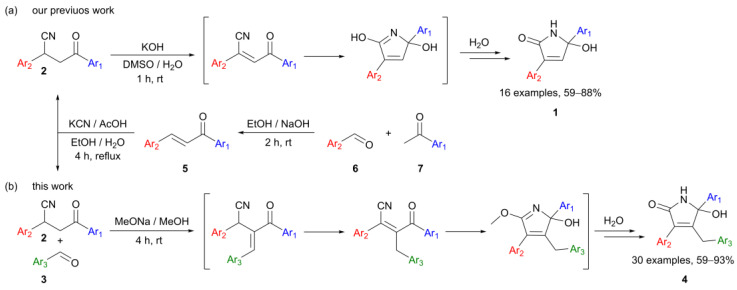
Synthesis of 3,5- and 3,4,5-substituted 5-hydroxy 3-pyrrolin-2-ones (**1** and **4**, correspondingly) by ring closure of 3-cyanoketones **2**.

Recently, we reported [1] the synthesis of a variety of 3,5-diaryl substituted 5-hydroxy 3-pyrrolin-2-ones **1** (referred therein for simplicity as γ-hydroxy-γ-lactams, γ-hydroxy butyrolactams or γ-hydroxy lactams) from readily available 3-cyanoketones **2** through a base-assisted intramolecular cyclization (Scheme 1a). As a continuation of this work, we envisioned that the core of those α,β-unsaturated γ-hydroxy lactams could be easily functionalized further at the C4-position by introducing into the reaction mixture a non-enolizable aldehyde **3** as an electrophilic component (Scheme 1b). In this case, in a similar manner to that described for the preparation of γ-hydroxy butenolides [2], an aldol condensation followed by double-bond isomerization and a base catalyzed cyclization should happen in one pot affording corresponding 3,4,5-trisubstituted 5-hydroxy-3-pyrrolin-2-ones **4**. Such highly functionalized γ-hydroxy butyrolactams, being an important subclass of 3-pyrrolin-2-ones [3,4], were found in the large number of biologically active natural products [5] either as simple heterocycles or as a part of more complex systems, including fused polycyclic ones. On the other hand, in addition to a hydroxyl group at the C5 quaternary carbon center, the skeleton of the herein-described lactams bears unsubstituted NH and conjugated enone moieties, meaning that these compounds can still be conveniently modified further [1,6] and, therefore, serve as useful synthetic intermediates in the preparation of other heterocyclic structures possessing interesting pharmacological properties.

It should be noticed that while numerous synthetic approaches to 5-hydroxy-3-pyrrolin-2-one derivatives have been reported [7,8,9,10,11,12], to our best knowledge, to date, there are just a few methods leading to 3,5-diaryl substituted γ-hydroxy butyrolactams **1** [1,13,14], and none that would afford the herein-described 3,5-di-aryl/heteroaryl-4-benzyl pattern. Thus, the developed procedures greatly expand the chemical space [15] of available 5-hydroxy 3-pyrrolin-2-ones which, in turn, could be used among other things for a convenient access to other heterocyclic systems [16].

## 2. Results and Discussion

**Scheme 2:**
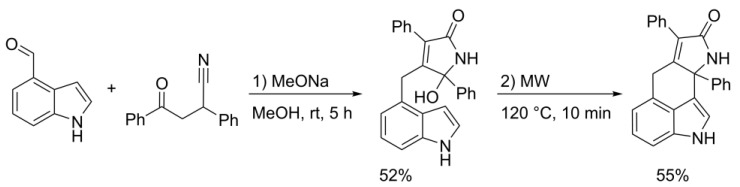
One-pot synthesis of polynuclear indoles based on intramolecular Friedel–Crafts alkylation with γ-hydroxy butyrolactams.

By the time we embarked on this project, well-functioning reaction conditions (KOH/DMSO/H_2_O, rt, 1 h) for the ring closure of β-ketonitriles **2** to 3,5 disubstituted γ-hydroxy lactams **1** had been established [1]. Additionally, the feasibility of preparation of 3,4,5-trisubstituted lactams **4** (Scheme 1b) was demonstrated earlier [16] with indole-4-carbaldehyde as a non-enolizable carbonyl component **3** (Scheme 2). Still, the scope and limitations of the proposed method as well as optimized conditions for the expected cascade transformation—aldol condensation, double bond isomerization and successive cyclization—were yet to be found. Therefore, a number of experiments probing the effects of solvents, bases, concentrations and reaction time were carried out (Table 1).

As can be seen above, the highest yield was achieved through simple stirring of the staring materials in methanol at room temperature for 4 h in the presence of a 4-fold excess of sodium methoxide (entry 1). The latter can be reduced to just one equivalent (entry 3), although at the expense of the reaction time (28 vs. 4 h). Additionally, it seems that the given combination—4 equiv. of MeONa in methanol—is superior both in terms of yields and experiment duration to the other studied systems (entries 5–12) utilizing different bases or/and solvents. This result is in agreement with our previous finding [16] where the herein-discussed synthetic methodology towards highly functionalized γ-hydroxy lactams were used to construct the LSD-like polycyclic indoles (Scheme 2).

**Scheme 3:**
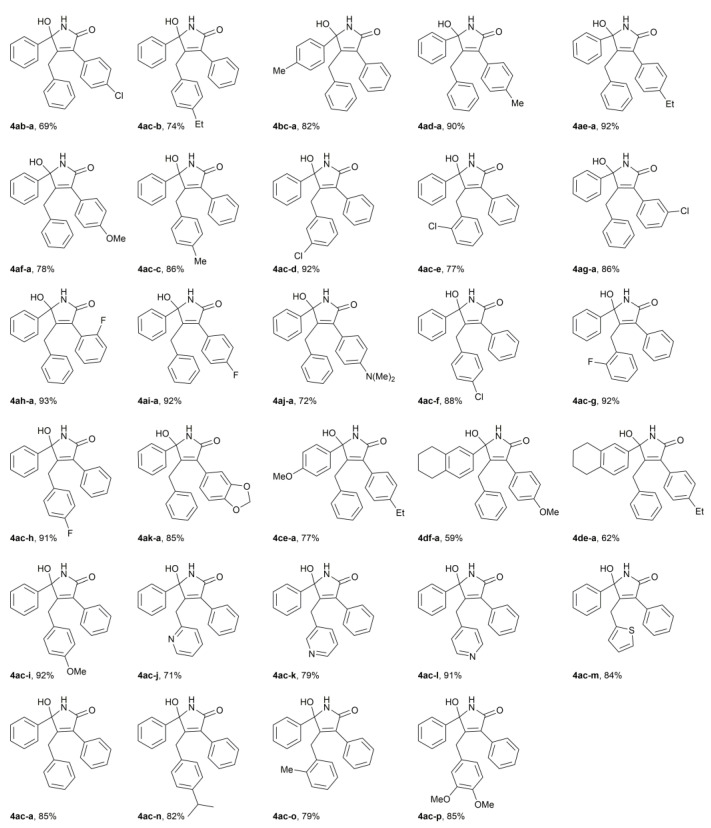
The library of novel 3,5-di-aryl/heteroaryl-4-benzyl-5-hydroxy 3-pyrrolin-2-ones **4** prepared by given procedure.

Thus, with the optimized reaction conditions in hand, we synthesized a focused, 30-substance-strong collection of novel 3,5-diaryl/heteroaryl-4-benzyl substituted 5-hydroxy 3-pyrrolin-2-ones **4** (Table 2 and Scheme 3). Its distinguished features are its generally good yields (59–93%) and its versatility in regard to the starting (hetero)aromatic aldehydes **3** and β-ketonitriles **2**. The latter can be conveniently prepared [1,2] from the corresponding substituted chalcones **5** which in turn are the products of typical cross aldol condensation between aldehydes **6** and acetophenones **7** (Scheme 1).

**Scheme 4:**
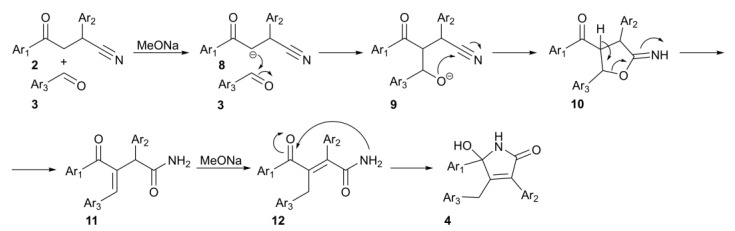
Plausible mechanism of a ring closure reaction between β-ketonitrile **2** and aromatic aldehyde **3**.

As for a mechanism of this cascade transformation, we assume it (Scheme 4) likely runs through the deprotonation of a cyanoketone **2** to a corresponding enolate **8**, that reacts further with an aldehyde **3** to give an intermediate **9**. The following intramolecular cycloaddition of the alkoxide ion into the nitrile group leads to an iminofuran **10**. The latter undergoes the ring-opening reaction to give chalcone **11**, which in turn under basic conditions is easily isomerized to an acrylamide **12**. The nucleophilic attack of the amide group at the carbonyl carbon furnishes target lactams **4**.

**Scheme 5:**
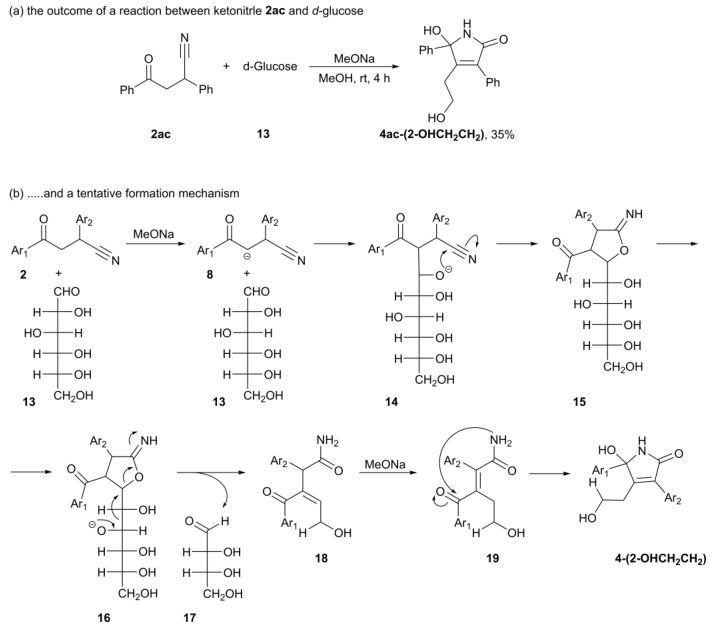
An unusual 5-hydroxy 3-pyrrolin-2-ones **4ac-(2-OHCH_2_CH_2_)** isolated after reaction of cyanoketone **2ac** with d-glucose **13** and a speculative mechanism of its formation.

In addition, we tried to run this reaction with a non-aromatic enolizable aldehyde, bearing an α-protons. For certain reasons, first we took the d-glucose **13**, and a γ-hydroxy lactam **4** indeed was obtained but not the one we expected (Scheme 5a).

It looks like the d-glucose served as a synthetic analog of glycolaldehyde. To address this observation, we propose the following mechanism (Scheme 5b) that in fact is very similar to that of Scheme 4. Thus, the condensation of a ketonitrile **2** with the glucose **13** gives an intermediate **14** which should further form an iminofuran **15**. At this stage, opposite to that shown in the Scheme 4, the ring opening of **15** is accompanied not by the elimination of a proton but by the loss of d-eritrose **17** fragment. Further transformation of **18** to **19** ends up eventually in the observed, seemingly unusual product **4-(2-OHCH_2_CH_2_)**. It should be noticed that the examples of glucose cleavage to glycolaldehyde and other small oxygenates, although in the rather harsh conditions (thermal cracking, hydrothermal pyrolysis, supercritical water, etc.), are known [17,18,19]; however, to our best knowledge, no precedents of such a transformation in a mild basic media have been reported. On the other hand, none of our attempts to use other aliphatic aldehydes as butyraldehyde, isobutyraldehyde and octanal were successful, probably due to the preferential self-condensation of the latter.

## 3. Materials and Methods

### 3.1. General Information

NMR spectra, ^1^H, ^13^C and ^19^F were measured in solutions of CDCl_3_ or DMSO-*d_6_* on a Bruker AVANCE-III HD instrument (at 400, 101 and 376 MHz, respectively). Residual solvent signals were used as internal standards, in DMSO*-d_6_* (2.50 ppm for ^1^H, and 40.45 ppm for ^13^C nuclei) or in CDCl_3_ (7.26 ppm for ^1^H, and 77.16 ppm for ^13^C nuclei). HRMS spectra was measured on a Bruker maXis impact (electrospray ionization, in MeCN solutions, employing HCO_2_Na–HCO_2_H for calibration). IR spectra was measured on an FT-IR spectrometer Shimadzu IRAffinity-1S equipped with an ATR sampling module. See Supplementary Materials for NMR (Figures S1–S73) spectral charts. Reaction progress, purity of isolated compounds, and R*_f_* values were monitored with TLC on Silufol UV-254 plates. Column chromatography was performed on silica gel (32–63 μm, 60 Å pore size). Melting points were measured with Stuart SMP30 apparatus. All 3-cyanoketones **2** and chalcones **5** except **5ce**, **5df**, **5de**, **2ce**, **2df** and **2de** were synthesized according to the previously reported procedures and were identical to those described [1]. All reagents and solvents were purchased from commercial venders and used as received.

### 3.2. Preparation of Chalcones **5ce**, **5df** and **5de** (General Procedure)

These compounds were prepared in analogy to the method described in [2]. The corresponding substituted benzaldehyde **6** (5.00 mmol) and acetophenone **7** (5.00 mmol) were dissolved in 5 mL of 96% ethanol then treated with 10% aqueous NaOH solution (0.2 mL). The mixture was stirred at room temperature for 2–4 h (TLC control) then concentrated in vacuo and crude material was purified by column chromatography (EtOAc/Hexane, *v*/*v*).

(E)-3-(4-Ethylphenyl)-1-(4-methoxyphenyl)prop-2-en-1-one (**5ce**): this compound was prepared employing 1-(4-methoxyphenyl)ethanone (750 mg, 5.00 mmol) and 4-ethylbenzaldehyde (670 mg, 5.00 mmol) in a yield of 891 mg (3.35 mmol, 67%). Purification was performed by column chromatography (EtOAc/Hexane = 1:4). The titled compound was obtained as yellow solid, m.p. 59.5–60.3 °C, R*_f_* 0.31 (EtOAc/Hexane = 1:4). ^1^H NMR (400 MHz, CDCl_3_) δ 8.09–8.00 (m, 2H), 7.80 (d, *J* = 15.7 Hz, 1H), 7.57 (d, *J* = 8.2 Hz, 2H), 7.52 (d, *J* = 15.7 Hz, 1H), 7.24 (d, *J* = 8.2 Hz, 2H), 7.02–6.94 (m, 2H), 3.87 (s, 3H), 2.68 (q, *J* = 7.6 Hz, 2H), 1.26 (t, *J* = 7.6 Hz, 3H); ^13^C{^1^H} NMR (101 MHz, CDCl_3_) δ 188.9, 163.4, 147.2, 144.2, 132.6, 131.3, 130.9 (2C), 128.6 (4C), 120.9, 113.9 (2C), 55.6, 28.9, 15.5; FTIR, *v_max_*: 2969, 1653, 1595, 1509, 1416, 1331, 1261, 1222, 1176, 1112, 989 cm^−1^; HRMS (ESI TOF) *m*/*z*: calculated for C_18_H_18_Na_1_O_2_ [M + Na]^+^: 289.1199, found 289.1207 (−2.8 ppm).

(E)-3-(4-Methoxyphenyl)-1-(5,6,7,8-tetrahydronaphthalen-2-yl)prop-2-en-1-one (**5df**): this compound was prepared employing 1-(5,6,7,8-tetrahydronaphthalen-2-yl)ethanone (870 mg, 5.00 mmol) and 4-methoxybenzaldehyde (680 mg, 5.00 mmol) in a yield of 1154 mg (3.95 mmol, 79%). Purification was performed by column chromatography (EtOAc/Hexane = 1:4). The titled compound was obtained as yellow solid, m.p. 94.3–95.8 °C, R*_f_* 0.31 (EtOAc/Hexane = 1:4). ^1^H NMR (400 MHz, CDCl_3_) δ 7.82–7.71 (m, 3H), 7.61 (d, *J* = 8.8 Hz, 2H), 7.42 (d, *J* = 15.7 Hz, 1H), 7.17 (d, *J* = 8.4 Hz, 1H), 6.93 (d, *J* = 7.6 Hz, 2H), 3.85 (s, 3H), 2.84 (d, *J* = 6.8 Hz, 4H), 1.83 (s, 4H); ^13^C{^1^H} NMR (101 MHz, CDCl_3_) δ 190.4, 161.6, 144.2, 142.9, 137.6, 136.0, 130.3 (2C), 129.5 (2C), 127.9, 125.7, 120.0, 114.5 (2C), 55.5, 29.8, 29.6, 23.1, 23.0; FTIR, *v_max_*: 2919, 1647, 1562, 1511, 1420, 1290, 1244, 1220, 1141, 1046, 984 cm^−1^; HRMS (ESI TOF) *m*/*z*: calculated for C_20_H_20_Na_1_O_2_ [M + Na]^+^: 315.1356, found 315.1364 (−2.5 ppm).

(E)-3-(4-Ethylphenyl)-1-(5,6,7,8-tetrahydronaphthalen-2-yl)prop-2-en-1-one (**5de-a**): this compound was prepared employing 1-(5,6,7,8-tetrahydronaphthalen-2-yl)ethanone (870 mg, 5.00 mmol) and 4-ethylbenzaldehyde (670 mg, 5.00 mmol) in a yield of 1030 mg (3.55 mmol, 71%). Purification was performed by column chromatography (EtOAc/Hexane = 1:10). The titled compound was obtained as yellow oil, R*_f_* 0.49 (EtOAc/Hexane = 1:10). ^1^H NMR (400 MHz, CDCl_3_) δ 7.79 (d, *J* = 15.6 Hz, 1H), 7.76–7.74 (m, 2H), 7.59–7.57 (m, 2H), 7.50 (d, *J* = 15.8 Hz, 1H), 7.25 (d, *J* = 6.8 Hz, 2H), 7.19–7.17 (m, 1H), 2.86–2.82 (m, 4H), 2.69 (q, *J* = 7.6 Hz, 2H), 1.86–1.81 (m, 4H), 1.26 (t, *J* = 7.6 Hz, 3H); ^13^C{^1^H} NMR (101 MHz, CDCl_3_) δ 190.5, 147.3, 144.4, 143.0, 137.6, 135.9, 132.7, 129.50, 129.46, 128.7 (2C), 128.6 (2C), 125.7, 121.4, 29.8, 29.5, 29.0, 23.1, 23.0, 15.5; FTIR, *v_max_*: 2930, 1913, 1659, 1595, 1564, 1515, 1424, 1323, 1226, 987 cm^−1^; HRMS (ESI TOF) *m*/*z*: calculated for C_21_H_22_Na_1_O_1_ [M + Na]^+^: 313.1563, found 313.1568 (−1.6 ppm).

### 3.3. Preparation of 3-cyanoketones **2ce**, **2df** and **2de** (General Procedure)

These compounds were prepared according to the method described in [1]. A 25 mL round bottom flask was charged with a corresponding chalcones **5** (3.00 mmol), KCN (4.5 mmol), EtOH (8 mL), water (0.5 mL) and acetic acid (0.17 mL). The reaction mixture was heated to reflux for 2–5 h (TLC control) then allowed to cool down to room temperature. The precipitated product was collected by filtration, washed with water and after drying at the open air purified if necessary by column chromatography (EtOAc/Hexane, *v*/*v*).

2-(4-Ethylphenyl)-4-(4-methoxyphenyl)-4-oxobutanenitrile (**2ce**): this compound was prepared employing (*E*)-3-(4-ethylphenyl)-1-(4-methoxyphenyl)prop-2-en-1-one (**5ce**) (798 mg, 3.00 mmol) in a yield of 759 mg (2.59 mmol, 86%). Purification was performed by column chromatography (EtOAc/Hexane = 1:2). The titled compound was obtained as white solid, m.p. 75.2–76.5 °C, R*_f_* 0.46 (EtOAc/Hexane = 1:2). ^1^H NMR (400 MHz, CDCl_3_) δ 7.90 (d, *J* = 8.9 Hz, 2H), 7.34 (d, *J* = 8.2 Hz, 2H), 7.21 (d, *J* = 8.3 Hz, 2H), 6.93 (d, *J* = 9.0 Hz, 2H), 4.53 (dd, *J* = 8.1, 6.0 Hz, 1H), 3.87 (s, 3H), 3.66 (dd, *J* = 17.7, 8.1 Hz, 1H), 3.44 (dd, *J* = 17.7, 6.1 Hz, 1H), 2.64 (q, *J* = 7.6 Hz, 2H), 1.23 (t, *J* = 7.6 Hz, 3H); ^13^C{1H} NMR (101 MHz, CDCl_3_) δ 193.3, 164.2, 144.6, 132.7, 130.6 (2C), 128.9, 128.8 (2C), 127.6 (2C), 121.1, 114.1 (2C), 55.7, 44.4, 31.7, 28.6, 15.6; FTIR, *v_max_*: 2243, 1676, 1604, 1568, 1513, 1364, 1316, 1260, 1214, 1173, 1023 cm^−1^; HRMS (ESI TOF) *m*/*z*: calculated for C_19_H_19_N_1_Na_1_O_2_ [M + Na]^+^: 316.1308, found 316.1302 (1.9 ppm).

2-(4-Methoxyphenyl)-4-oxo-4-(5,6,7,8-tetrahydronaphthalen-2-yl)butanenitrile (**2df**): this compound was prepared employing (*E*)-3-(4-methoxyphenyl)-1-(5,6,7,8-tetrahydronaphthalen-2-yl)prop-2-en-1-one (**5df**) (876 mg, 3.00 mmol) in a yield of 862 mg (2.70 mmol, 90%). Purification was performed by column chromatography (EtOAc/Hexane = 1:4). The titled compound was obtained as yellow oil, R*_f_* 0.34 (EtOAc/Hexane = 1:4). ^1^H NMR (400 MHz, CDCl_3_) δ 7.62 (d, *J* = 4.9 Hz, 2H), 7.34 (d, *J* = 8.8 Hz, 2H), 7.13 (d, *J* = 8.6 Hz, 1H), 6.90 (d, *J* = 8.9 Hz, 2H), 4.55–4.47 (m, 1H), 3.80 (s, 3H), 3.65 (dd, *J* = 17.8, 7.6 Hz, 1H), 3.45 (dd, *J* = 17.8, 6.4 Hz, 1H), 2.79 (d, *J* = 4.2 Hz, 4H), 1.86–1.74 (m, 4H); ^13^C{1H} NMR (101 MHz, CDCl_3_) δ 194.8, 159.5, 144.3, 137.8, 133.3, 129.7, 129.1, 128.8 (2C), 127.4, 125.2, 121.2, 114.6 (2C), 55.5, 44.5, 31.2, 29.8, 29.4, 23.0, 22.8; FTIR, *v_max_*: 2247, 1678, 1608, 1509, 1415, 1348, 1248, 1181, 1029 cm^−1^; HRMS (ESI TOF) *m*/*z*: calculated for C_21_H_21_N_1_Na_1_O_2_ [M + Na]^+^: 342.1464, found 342.1466 (−0.4 ppm).

2-(4-Ethylphenyl)-4-oxo-4-(5,6,7,8-tetrahydronaphthalen-2-yl)butanenitrile (**2de**): this compound was prepared employing (*E*)-3-(4-ethylphenyl)-1-(5,6,7,8-tetrahydronaphthalen-2-yl)prop-2-en-1-one (**5de**) (870 mg, 3.00 mmol) in a yield of 637 mg (2.01 mmol, 67%). Purification was performed by column chromatography (EtOAc/Hexane = 1:2). The titled compound was obtained as yellow oil, R*_f_* 0.23 (EtOAc/Hexane = 1:2). ^1^H NMR (400 MHz, CDCl_3_) δ 7.63 (d, *J* = 6.5 Hz, 2H), 7.34 (d, *J* = 8.2 Hz, 2H), 7.22 (d, *J* = 8.4 Hz, 2H), 7.14 (d, *J* = 8.4 Hz, 1H), 4.53 (dd, *J* = 8.0, 6.1 Hz, 1H), 3.67 (dd, *J* = 17.9, 7.9 Hz, 1H), 3.46 (dd, *J* = 17.9, 6.0 Hz, 1H), 2.79 (d, *J* = 5.5 Hz, 4H), 2.65 (q, *J* = 7.6 Hz, 2H), 1.81 (s, 4H), 1.23 (t, *J* = 7.6 Hz, 3H); ^13^C{1H} NMR (101 MHz, CDCl_3_) δ 194.7, 144.5, 144.2, 137.8, 133.3, 132.7, 129.6, 129.1, 128.8 (2C), 127.5 (2C), 125.2, 121.1, 44.5, 31.6, 29.8, 29.4, 28.6, 22.9, 22.8, 15.6; FTIR, *v_max_*: 2247, 1676, 1602, 1515, 1419, 1354, 1258, 1139, 1020 cm^−1^; HRMS (ESI TOF) *m*/*z*: calculated for C_22_H_23_N_1_Na_1_O_1_ [M + Na]^+^: 340.1672, found 340.1670 (0.5 ppm).

### 3.4. Preparation of γ-hydroxy Butyrolactams **4** (General Procedure)

A 5 mL round bottom flask was charged with a corresponding cyanoketone **2** (1.00 mmol), benzaldehyde **3** (1.05 mmol), methanol (2 mL) and sodium methoxide (4 mmol). The mixture was stirred at room temperature for 4 h (TLC control) then diluted with ethyl acetate (10 mL), washed with water (2 mL), 10% solution of NaHCO_3_ (2 mL) and brine (2 mL). After drying over sodium sulfate, the solution was concentrated in vacuo and the obtained residue was purified by column chromatography (EtOAc/Hexane, *v*/*v*).

4-Benzyl-3-(4-chlorophenyl)-5-hydroxy-5-phenyl-1H-pyrrol-2(5H)-one (**4ab-a**): this compound was prepared employing 2-(4-chlorophenyl)-4-oxo-4-phenylbutanenitrile **2ab** [1] (269 mg, 1.00 mmol) and benzaldehyde **3a** (111 mg, 1.05 mmol) in a yield of 259 mg (0.69 mmol, 69%). Purification was performed by column chromatography (EtOAc/Hexane = 1:2). The titled compound was obtained as colorless oil, R*_f_* 0.49 (EtOAc/Hexane = 1:2). ^1^H NMR (400 MHz, DMSO-*d_6_*) δ 9.13 (s, 1H), 7.50–7.43 (m, 2H), 7.43–7.37 (m, 2H), 7.35–7.21 (m, 5H), 6.99–6.90 (m, 3H), 6.81–6.75 (m, 2H), 6.70 (s, 1H), 3.63 (d, *J* = 16.3 Hz, 1H), 3.58 (d, *J* = 16.3 Hz, 1H); ^13^C{^1^H} NMR (101 MHz, DMSO-*d_6_*) δ 170.9, 157.9, 140.0, 137.2, 132.4, 130.8 (2C), 130.1, 129.5 (2C), 128.5, 128.2, 127.9 (2C), 127.8 (2C), 127.7 (2C), 126.1 (2C), 125.7, 88.3, 30.8; FTIR, *v_max_*: 3308, 3062, 1695, 1491, 1453, 1372, 1236, 1093 cm^−1^; HRMS (ESI TOF) *m*/*z*: calculated for C_23_H_18_N_1_NaO_2_ [M + Na]^+^: 398.0918, found 398.0919 (−0.2 ppm).

4-Benzyl-5-hydroxy-3,5-diphenyl-1H-pyrrol-2(5H)-one (**4ac-a**): this compound was prepared employing 4-oxo-2,4-diphenylbutanenitrile **2ac** [1] (235 mg, 1.00 mmol) and benzaldehyde **3a** (111 mg, 1.05 mmol) in a yield of 290 mg (0.85 mmol, 85%). Purification was performed by column chromatography (EtOAc/Hexane = 1:2). The titled compound was obtained as a yellow amorphous solid, m.p. 218.5–220.0 °C, R*_f_* 0.57 (EtOAc/Hexane = 1:2). ^1^H NMR (400 MHz, DMSO-*d_6_*) δ 9.10 (d, *J* = 1.8 Hz, 1H), 7.47–7.42 (m, 2H), 7.40–7.35 (m, 2H), 7.32–7.20 (m, 6H), 6.99–6.90 (m, 3H), 6.79–6.62 (m, 3H), 3.58 (t, *J* = 15.7 Hz, 2H); ^13^C{^1^H} NMR (101 MHz, DMSO-*d_6_*) δ 171.2, 157.0, 140.3, 137.5, 131.3, 130.8, 129.0 (2C), 128.4 (2C), 128.1 (2C), 127.8 (2C), 127.7 (4C), 126.1 (2C), 125.6, 88.2, 30.8; FTIR, *v_max_*: 3329, 3062, 1698, 1489, 1443, 1374, 1125, 1057 cm^−1^; HRMS (ESI TOF) *m*/*z*: calculated for C_23_H_19_N_1_Na_1_O_2_ [M + Na]^+^: 364.1308, found 364.1312 (−1.2 ppm).

4-(4-Ethylbenzyl)-5-hydroxy-3,5-diphenyl-1H-pyrrol-2(5H)-one (**4ac-b**): this compound was prepared employing 4-oxo-2,4-diphenylbutanenitrile **2ac** [1] (235 mg, 1.00 mmol) and 4-ethylbenzaldehyde **3b** (141 mg, 1.05 mmol) in a yield of 273 mg (0.74 mmol, 74%). Purification was performed by column chromatography (EtOAc/Hexane = 1:2). The titled compound was obtained as a yellow amorphous solid, m.p. 175.5–177.3 °C, R*_f_* 0.51 (EtOAc/Hexane = 1:2). ^1^H NMR (400 MHz, DMSO-*d_6_*) δ 9.02 (s, 1H), 7.46–7.34 (m, 4H), 7.34–7.18 (m, 6H), 6.77 (d, *J* = 7.9 Hz, 2H), 6.65 (d, *J* = 7.8 Hz, 2H), 6.59 (s, 1H), 3.55 (d, *J* = 16.5 Hz, 2H), 3.51 (d, *J* = 16.5 Hz, 2H), 2.40 (q, *J* = 7.6 Hz, 2H), 1.04 (t, *J* = 7.6 Hz, 3H); ^13^C{^1^H} NMR (101 MHz, DMSO-*d_6_*) δ 171.2, 157.2, 140.8, 140.2, 134.6, 131.4, 130.7, 129.0 (2C), 128.4 (2C), 128.1 (2C), 127.8 (2C), 127.7 (2C), 127.1 (2C), 126.0 (2C), 88.2, 30.5, 27.7, 15.7; FTIR, *v_max_*: 3396, 3277, 2970, 1690, 1510, 1493, 1447, 1195, 1121, 1063 cm^−1^; HRMS (ESI TOF) *m*/*z*: calculated for C_25_H_23_N_1_Na_1_O_2_ [M + Na]^+^: 392.1621, found 392.1621 (0.1 ppm).

5-Hydroxy-4-(4-methylbenzyl)-3,5-diphenyl-1H-pyrrol-2(5H)-one (**4ac-c**): this compound was prepared employing 4-oxo-2,4-diphenylbutanenitrile **2ac** [1] (235 mg, 1.00 mmol) and 4-methylbenzaldehyde **3c** (126 mg, 1.05 mmol) in a yield of 305 mg (0.86 mmol, 86%). Purification was performed by column chromatography (EtOAc/Hexane = 1:2). The titled compound was obtained as white amorphous solid, m.p. 214.1–215.4 °C, R*_f_* 0.57 (EtOAc/Hexane = 1:2). ^1^H NMR (400 MHz, DMSO-*d_6_*) δ 9.02 (s, 1H), 7.47–7.40 (m, 2H), 7.40–7.34 (m, 2H), 7.34–7.22 (m, 6H), 6.75 (d, *J* = 7.8 Hz, 2H), 6.62 (d, *J* = 7.8 Hz, 2H), 6.58 (s, 1H), 3.55 (d, *J* = 15.9 Hz, 1H), 3.49 (d, *J* = 15.8 Hz, 1H), 2.10 (s, 3H); ^13^C{^1^H} NMR (101 MHz, DMSO-*d_6_*) δ 171.2, 157.2, 140.2, 134.4 (2C), 131.4, 130.7, 129.0 (2C), 128.3 (4C), 128.1 (2C), 127.8 (2C), 127.7 (2C), 126.1 (2C), 88.2, 30.4, 20.5; FTIR, *v_max_*: 3535, 3193, 3074, 1699, 1513, 1489, 1447, 1370, 1244, 1119, 1053 cm^−1^; HRMS (ESI TOF) *m*/*z*: calculated for C_24_H_21_N_1_Na_1_O_2_ [M + Na]^+^: 378.1464, found 378.1455 (2.5 ppm).

4-(3-Chlorobenzyl)-5-hydroxy-3,5-diphenyl-1H-pyrrol-2(5H)-one (**4ac-d**): this compound was prepared employing 4-oxo-2,4-diphenylbutanenitrile **2ac** [1] (235 mg, 1.00 mmol) and 3-chlorobenzaldehyde **3d** (147 mg, 1.05 mmol) in a yield of 345 mg (0.92 mmol, 92%). Purification was performed by column chromatography (EtOAc/Hexane = 1:2). The titled compound was obtained as a yellow amorphous solid, m.p. 139.0–140.6 °C, R*_f_* 0.49 (EtOAc/Hexane = 1:2). ^1^H NMR (400 MHz, DMSO-*d_6_*) δ 9.08 (s, 1H), 7.46–7.40 (m, 2H), 7.37–7.22 (m, 8H), 7.00–6.92 (m, 2H), 6.73 (dt, *J* = 8.7, 1.8 Hz, 2H), 6.70 (s, 1H), 3.59 (t, *J* = 16.7 Hz, 2H); ^13^C{^1^H} NMR (101 MHz, DMSO-*d_6_*) δ 171.0, 156.4, 140.0, 139.8, 132.4, 131.1, 131.0, 129.3, 129.0 (2C), 128.4, 128.2 (2C), 127.9 (3C), 127.8, 127.2, 126.0 (2C), 125.6, 88.1, 30.3; FTIR, *v_max_*: 3396, 3273, 3062, 1696, 1598, 1489, 1451, 1429, 1199, 1101, 1069 cm^−1^; HRMS (ESI TOF) *m*/*z*: calculated for C_23_H_18_Cl_1_N_1_Na_1_O_2_ [M + Na]^+^: 398.0918, found 398.0917 (0.3 ppm).

4-(2-Chlorobenzyl)-5-hydroxy-3,5-diphenyl-1H-pyrrol-2(5H)-one (**4ac-e**): this compound was prepared employing 4-oxo-2,4-diphenylbutanenitrile **2ac** [1] (235 mg, 1.00 mmol) and 2-chlorobenzaldehyde **3e** (147 mg, 1.05 mmol) in a yield of 289 mg (0.77 mmol, 77%). Purification was performed by column chromatography (EtOAc/Hexane = 1:2). The titled compound was obtained as a white amorphous solid, m.p. 155.0–156.0 °C, R*_f_* 0.46 (EtOAc/Hexane = 1:2). ^1^H NMR (400 MHz, DMSO-*d_6_*) δ 9.12 (s, 1H), 7.45–7.39 (m, 2H), 7.35–7.18 (m, 8H), 7.14 (d, *J* = 7.9 Hz, 1H), 6.97 (t, *J* = 7.6 Hz, 1H), 6.89 (t, *J* = 7.5 Hz, 1H), 6.82 (d, *J* = 7.7 Hz, 1H), 6.69 (d, *J* = 1.0 Hz, 1H), 3.69 (d, *J* = 17.0 Hz, 1H), 3.61 (d, *J* = 17.0 Hz, 1H); ^13^C{^1^H} NMR (101 MHz, DMSO-*d_6_*) δ 170.9, 155.3, 140.0, 134.4, 132.8, 131.6, 131.1, 130.1, 128.7 (2C), 128.6, 128.1 (2C), 127.8 (4C), 127.7, 126.5, 125.9 (2C), 88.1, 28.6; FTIR, *v_max_*: 3404, 3273, 1695, 1672, 1447, 1423, 1195, 1069 cm^−1^; HRMS (ESI TOF) *m*/*z*: calculated for C_23_H_18_Cl_1_N_1_Na_1_O_2_ [M + Na]^+^: 398.0918, found 398.0921 (−0.6 ppm).

4-(4-Chlorobenzyl)-5-hydroxy-3,5-diphenyl-1H-pyrrol-2(5H)-one (**4ac-f**): this compound was prepared employing 4-oxo-2,4-diphenylbutanenitrile **2ac** [1] (235 mg, 1.00 mmol) and 4-chlorobenzaldehyde **3f** (147 mg, 1.05 mmol) in a yield of 330 mg (0.88 mmol, 88%). Purification was performed by column chromatography (EtOAc/Hexane = 1:2). The titled compound was obtained as a white amorphous solid, m.p. 217.4–218.4 °C, R*_f_* 0.57 (EtOAc/Hexane = 1:2). ^1^H NMR (400 MHz, DMSO-*d_6_*) δ 9.07 (s, 1H), 7.43 (d, *J* = 7.5 Hz, 2H), 7.37 (d, *J* = 6.4 Hz, 2H), 7.29 (q, *J* = 6.5 Hz, 6H), 6.98 (d, *J* = 8.1 Hz, 2H), 6.79 (d, *J* = 8.1 Hz, 2H), 6.66 (s, 1H), 3.57 (t, *J* = 17.1 Hz, 2H); ^13^C{^1^H} NMR (101 MHz, DMSO-*d_6_*) δ 171.0, 156.6, 140.1, 136.4, 131.11, 131.0, 130.3 (2C), 130.2, 129.0 (2C), 128.2 (2C), 127.9 (2C), 127.8 (2C), 127.5 (2C), 126.0 (2C), 88.1, 30.1; FTIR, *v_max_*: 3521, 3193, 1701, 1490, 1449, 1360, 1246, 1204, 1051 cm^−1^; HRMS (ESI TOF) *m*/*z*: calculated for C_23_H_18_Cl_1_N_1_Na_1_O_2_ [M + Na]^+^: 398.0918, found 398.0916 (0.4 ppm).

4-(2-Fluorobenzyl)-5-hydroxy-3,5-diphenyl-1H-pyrrol-2(5H)-one (**4ac-g**): this compound was prepared employing 4-oxo-2,4-diphenylbutanenitrile **2ac** [1] (235 mg, 1.00 mmol) and 2-fluorobenzaldehyde **3g** (130 mg, 1.05 mmol) in a yield of 330 mg (0.92 mmol, 92%). Purification was performed by column chromatography (EtOAc/Hexane = 1:2). The titled compound was obtained as colorless oil, R*_f_* 0.51 (EtOAc/Hexane = 1:2). ^1^H NMR (400 MHz, DMSO-*d_6_*) δ 9.08 (s, 1H), 7.44–7.38 (m, 2H), 7.37–7.32 (m, 2H), 7.32–7.17 (m, 7H), 7.03–6.94 (m, 1H), 6.86–6.78 (m, 1H), 6.78–6.72 (m, 2H), 3.72–3.42 (m, 2H); ^13^C{^1^H} NMR (101 MHz, DMSO-*d_6_*) δ 171.0, 160.0 (d, *J* = 243.6 Hz), 155.6, 140.0, 131.5, 131.1, 130.4 (d, *J* = 4.0 Hz), 128.9 (2C), 128.1 (2C), 127.9 (d, *J* = 7.9 Hz), 127.9 (2C), 127.8 (2C), 125. 9 (2C), 123.8 (d, *J* = 15.2 Hz), 123.7 (d, *J* = 3.4 Hz), 114.5 (d, *J* = 21.9 Hz), 88.1, 23.7 (d, *J* = 4.1 Hz); ^19^F NMR (376 MHz, DMSO-*d_6_*) δ −117.23; FTIR, *v_max_*: 3332, 3058, 1690, 1497, 1447, 1232, 1095 cm^−1^; HRMS (ESI TOF) *m*/*z*: calculated for C_23_H_18_F_1_N_1_Na_1_O_2_ [M + Na]^+^: 382.1214, found 382.1215 (−0.4 ppm).

4-(4-Fluorobenzyl)-5-hydroxy-3,5-diphenyl-1H-pyrrol-2(5H)-one (**4ac-h**): this compound was prepared employing 4-oxo-2,4-diphenylbutanenitrile **2ac** [1] (235 mg, 1.00 mmol) and 4-fluorobenzaldehyde **3h** (130 mg, 1.05 mmol) in a yield of 327 mg (0.91 mmol, 91%). Purification was performed by column chromatography (EtOAc/Hexane = 1:2). The titled compound was obtained as a white solid, m.p. 215.2–216.0 °C, R*_f_* 0.43 (EtOAc/Hexane = 1:2). ^1^H NMR (400 MHz, DMSO-*d_6_*) δ 9.05 (d, *J* = 2.1 Hz, 1H), 7.51–7.19 (m, 10H), 6.84–6.68 (m, 4H), 6.65 (d, *J* = 2.1 Hz, 1H), 3.59 (t, *J* = 16.3 Hz, 2H); ^13^C{^1^H} NMR (101 MHz, DMSO-*d_6_*) δ 171.5, 160.7 (d, *J* = 241.4 Hz), 157.4, 140.6, 133.9 (d, *J* = 2.9 Hz), 131.6, 131.3, 130.6 (2C, d, *J* = 8.1 Hz), 129.5 (2C), 128.6 (2C), 128.3 (2C), 128.2 (2C), 126.5 (2C), 114.7 (2C, d, *J* = 21.1 Hz), 88.5, 30.4; FTIR, *v_max_*: 3444, 3352, 3093, 2855, 194, 1504, 1451, 1218, 1153, 1153, 1091, 1061 cm^−1^; HRMS (ESI TOF) *m*/*z*: calculated for C_23_H_18_F_1_N_1_Na_1_O_2_ [M + Na]^+^: 382.1214, found 382.1220 (−1.7 ppm).

5-Hydroxy-4-(4-methoxybenzyl)-3,5-diphenyl-1H-pyrrol-2(5H)-one (**4ac-i**): this compound was prepared employing 4-oxo-2,4-diphenylbutanenitrile **2ac** [1] (235 mg, 1.00 mmol) and 4-methoxybenzaldehyde **3i** (143 mg, 1.05 mmol) in a yield of 341 mg (0.92 mmol, 92%). Purification was performed by column chromatography (EtOAc/Hexane = 1:2). The titled compound was obtained as a white solid, m.p. 176.5–177.7 °C, R*_f_* 0.42 (EtOAc/Hexane = 1:2). ^1^H NMR (400 MHz, DMSO-*d_6_*) δ 9.03 (s, 1H), 7.46–7.41 (m, 2H), 7.41–7.37 (m, 2H), 7.33–7.22 (m, 6H), 6.66 (d, *J* = 8.3 Hz, 2H), 6.61 (s, 1H), 6.50 (d, *J* = 8.5 Hz, 2H), 3.58 (s, 3H), 3.52 (t, *J* = 16.3 Hz, 2H); ^13^C{^1^H} NMR (101 MHz, DMSO-*d_6_*) δ 171.22, 157.48, 140.26, 131.37, 130.58, 129.39 (2C), 129.31 (2C), 129.03 (2C), 128.11 (2C), 127.82, 127.73 (2C), 127.70, 126.06 (2C), 113.10 (2C), 88.19, 54.87, 29.95; FTIR, *v_max_*: 3332, 1692, 1502, 1455, 1415, 1240, 1171, 1107, 1061 cm^−1^; HRMS (ESI TOF) *m*/*z*: calculated for C_24_H_21_N_1_Na_1_O_3_ [M + Na]^+^: 394.1414, found 394.1412 (0.4 ppm).

5-Hydroxy-3,5-diphenyl-4-(pyridin-2-ylmethyl)-1H-pyrrol-2(5H)-one (**4ac-j**): this compound was prepared employing 4-oxo-2,4-diphenylbutanenitrile **2ac** [1] (235 mg, 1.00 mmol) and pyridine-2-carbaldehyde **3j** (112 mg, 1.05 mmol) in a yield of 243 mg (0.71 mmol, 71%). Purification was performed by column chromatography (EtOAc/Hexane = 1:2). The titled compound was obtained as a white solid, m.p. 224.8–226.7 °C, R*_f_* 0.34 (EtOAc/Hexane = 1:2). ^1^H NMR (400 MHz, DMSO-*d_6_*) δ 9.08 (s, 1H), 8.33 (dd, *J* = 4.9, 1.8 Hz, 1H), 7.56–7.52 (m, 2H), 7.46 (td, *J* = 7.7, 1.9 Hz, 1H), 7.42–7.16 (m, 9H), 7.06 (dd, *J* = 7.5, 4.9 Hz, 1H), 6.95 (d, *J* = 7.8 Hz, 1H), 3.77 (d, *J* = 15.8 Hz, 1H), 3.62 (d, *J* = 15.7 Hz, 1H); ^13^C{^1^H} NMR (101 MHz, DMSO-*d_6_*) δ 170.9, 157.5, 155.1, 148.5, 140.4, 136.5, 131.4, 131.0, 129.0 (2C), 128.0 (3C), 127.9 (2C), 127.6, 126.0 (2C), 123.3, 121.4, 88.0, 33.9; FTIR, *v_max_*: 3209, 3062, 2815, 1696, 1491, 1361, 1202, 1113, 1065 cm^−1^; HRMS (ESI TOF) *m*/*z*: calculated for C_22_H_18_N_2_Na_1_O_2_ [M + Na]^+^: 365.1260, found 365.1261 (−0.2 ppm).

5-Hydroxy-3,5-diphenyl-4-(pyridin-3-ylmethyl)-1H-pyrrol-2(5H)-one (**4ac-k**): this compound was prepared employing 4-oxo-2,4-diphenylbutanenitrile **2ac** [1] (235 mg, 1.00 mmol) and pyridine-3-carbaldehyde (112 mg, 1.05 mmol) in a yield of 270 mg (0.79 mmol, 79%). Purification was performed by column chromatography (EtOAc). The titled compound was obtained as a white solid, m.p. 199.1–200.7 °C, R*_f_* 0.3 (EtOAc). ^1^H NMR (400 MHz, DMSO-*d_6_*) δ 9.08 (s, 1H), 8.11 (dd, *J* = 4.8, 1.6 Hz, 1H), 7.98 (d, *J* = 2.2 Hz, 1H), 7.48–7.41 (m, 2H), 7.38–7.22 (m, 8H), 7.08 (dt, *J* = 7.9, 2.0 Hz, 1H), 6.92 (dd, *J* = 7.7, 4.8 Hz, 1H), 6.70 (s, 1H), 3.59 (t, *J* = 16.7 Hz, 2H); ^13^C{^1^H} NMR (101 MHz, DMSO-*d_6_*) δ 171.0, 156.3, 149.6, 146.8, 140.0, 135.6, 133.0, 131.0 (2C), 129.0 (2C), 128.2 (2C), 127.9 (3C), 127.8, 126.0 (2C), 122.7, 88.1, 28.0; FTIR, *v_max_*: 3412, 3300, 2998, 1709, 1485, 1441, 1254, 1204, 1095, 1047 cm^−1^; HRMS (ESI TOF) *m*/*z*: calculated for C_22_H_19_N_2_O_2_ [M + H]^+^: 343.1441, found 343.1446 (−1.3 ppm).

5-Hydroxy-3,5-diphenyl-4-(pyridin-4-ylmethyl)-1H-pyrrol-2(5H)-one (**4ac-l**): this compound was prepared employing 4-oxo-2,4-diphenylbutanenitrile **2ac** [1] (235 mg, 1.00 mmol) and pyridine-4-carbaldehyde **3l** (112 mg, 1.05 mmol) in a yield of 311 mg (0.91 mmol, 91%). Purification was performed by column chromatography (EtOAc). The titled compound was obtained as a white solid, m.p. 216.9–217.9 °C, R*_f_* 0.34 (EtOAc). ^1^H NMR (400 MHz, DMSO-*d_6_*) δ 9.13 (s, 1H), 8.11 (d, *J* = 5.3 Hz, 2H), 7.44 (d, *J* = 7.2 Hz, 2H), 7.38 (d, *J* = 6.9 Hz, 2H), 7.34–7.18 (m, 6H), 6.78 (d, *J* = 5.2 Hz, 2H), 6.70 (s, 1H), 3.60 (t, *J* = 17.9 Hz, 2H); ^13^C{^1^H} NMR (101 MHz, DMSO-*d_6_*) δ 170.9, 155.4, 148.8 (2C), 146.4, 139.8, 131.4, 131.0, 128.9 (2C), 128.2 (2C), 127.9 (3C), 127.9, 126.1 (2C), 123.8 (2C), 88.0, 30.1; FTIR, *v_max_*: 3193, 3065, 2819, 1705, 1602, 1423, 1361, 1248, 1208, 1103, 1061 cm^−1^; HRMS (ESI TOF) *m*/*z*: calculated for C_22_H_19_N_2_O_2_ [M + H]^+^: 343.1441, found 343.1449 (−2.4 ppm).

5-Hydroxy-3,5-diphenyl-4-(thiophen-2-ylmethyl)-1H-pyrrol-2(5H)-one (**4ac-m**): this compound was prepared employing 4-oxo-2,4-diphenylbutanenitrile **2ac** [1] (235 mg, 1.00 mmol) and thiophene-2-carbaldehyde **3m** (118 mg, 1.05 mmol) in a yield of 291 mg (0.84 mmol, 84%). Purification was performed by column chromatography (EtOAc/Hexane = 1:2). The titled compound was obtained as a white solid, m.p. 193.2–194.5 °C, R*_f_* 0.46 (EtOAc/Hexane = 1:2). ^1^H NMR (400 MHz, DMSO-*d_6_*) δ 9.06 (s, 1H), 7.48–7.40 (m, 4H), 7.39–7.23 (m, 6H), 7.04 (dd, *J* = 5.1, 1.2 Hz, 1H), 6.67 (s, 1H), 6.58 (dd, *J* = 5.2, 3.4 Hz, 1H), 6.28 (dd, *J* = 3.5, 1.2 Hz, 1H), 3.74 (s, 2H); ^13^C{^1^H} NMR (101 MHz, DMSO-*d_6_*) δ 171.0, 156.3, 139.9, 139.6, 131.2, 130.7, 129.0 (2C), 128.2 (2C), 128.0 (3C), 127.9, 126.2, 126.1 (2C), 125.8, 124.0, 88.0, 25.1; FTIR, *v_max_*: 3444, 3292, 1694, 1511, 1459, 1370, 1244, 1059 cm^−1^; HRMS (ESI TOF) *m*/*z*: calculated for C_21_H_17_N_1_Na_1_O_2_S_1_ [M + Na]^+^: 370.0872, found 370.0877 (−1.2 ppm).

5-Hydroxy-4-(4-isopropylbenzyl)-3,5-diphenyl-1H-pyrrol-2(5H)-one (**4ac-n**): this compound was prepared employing 4-oxo-2,4-diphenylbutanenitrile **2ac** [1] (235 mg, 1.00 mmol) and 4-isopropylbenzaldehyde **3n** (155 mg, 1.05 mmol) in a yield of 314 mg (0.82 mmol, 82%). Purification was performed by column chromatography (EtOAc/Hexane = 1:2). The titled compound was obtained as a yellow amorphous solid, m.p. 142.9–144.7 °C, R*_f_* 0.46 (EtOAc/Hexane = 1:2). ^1^H NMR (400 MHz, DMSO-*d_6_*) δ 9.01 (s, 1H), 7.44–7.34 (m, 4H), 7.33–7.18 (m, 6H), 6.79 (d, *J* = 8.0 Hz, 2H), 6.65 (d, *J* = 7.9 Hz, 2H), 6.60 (s, 1H), 3.53 (s, 2H), 2.75–2.60 (m, 1H), 1.06 (d, *J* = 6.9 Hz, 6H); ^13^C{^1^H} NMR (101 MHz, DMSO-*d_6_*) δ 171.1, 157.3, 145.4, 140.3, 134.7, 131.4, 130.8, 129.0 (2C), 128.4 (2C), 128.0 (2C), 127.8 (2C), 127.7, 127.6, 126.0 (2C), 125.5 (2C), 88.2, 32.9, 30.5, 23.9 (2C); FTIR, *v_max_*: 3395, 3261, 1670, 1443,1204, 1097, 1055 cm^−1^; HRMS (ESI TOF) *m*/*z*: calculated for C_26_H_25_N_1_Na_1_O_2_ [M + Na]^+^: 406.1784, found 406.1777 (−1.6 ppm).

5-Hydroxy-4-(2-methylbenzyl)-3,5-diphenyl-1H-pyrrol-2(5H)-one (**4ac-o**): this compound was prepared employing 4-oxo-2,4-diphenylbutanenitrile **2ac** [1] (235 mg, 1.00 mmol) and 2-methylbenzaldehyde **3o** (126 mg, 1.05 mmol) in a yield of 280 mg (0.79 mmol, 79%). Purification was performed by column chromatography (EtOAc/Hexane = 1:2). The titled compound was obtained as a white solid, m.p. 215.2–216.3 °C, R*_f_* 0.57 (EtOAc/Hexane = 1:2). ^1^H NMR (400 MHz, DMSO-*d_6_*) δ 9.06 (s, 1H), 7.43–7.39 (m, 2H), 7.34–7.17 (m, 8H), 6.88–6.81 (m, 2H), 6.79–6.72 (m, 1H), 6.69 (d, *J* = 7.6 Hz, 1H), 6.62 (s, 1H), 3.55 (d, *J* = 16.6 Hz, 1H), 3.46 (d, *J* = 16.6 Hz, 1H), 1.98 (s, 3H);^13^C{^1^H} NMR (101 MHz, DMSO-*d_6_*) δ 171.1, 156.7, 140.3, 135.4, 135.3, 131.3(2C), 129.4, 128.8 (2C), 128.3, 128.0 (2C), 127.8 (2C), 127.7, 127.6, 125.9 (2C), 125.7, 125.2, 88.2, 28.6, 19.4; FTIR, *v_max_*: 3547, 3169, 3066, 1711, 1497, 1449, 1364, 1290, 1177, 1107, 1049 cm^−1^; HRMS (ESI TOF) *m*/*z*: calculated for C_24_H_21_N_1_Na_1_O_2_ [M + Na]^+^: 378.1464, found 378.1461 (0.9 ppm).

4-(3,4-Dimethoxybenzyl)-5-hydroxy-3,5-diphenyl-1H-pyrrol-2(5H)-one (**4ac-p**): this compound was prepared employing 4-oxo-2,4-diphenylbutanenitrile **2ac** [1] (235 mg, 1.00 mmol) and 3,4-dimethoxybenzaldehyde **3p** (173 mg, 1.05 mmol) in a yield of 349 mg (0.87 mmol, 87%). Purification was performed by column chromatography (EtOAc/Hexane = 1:1). The titled compound was obtained as a white solid, m.p. 197.0–198.8 °C, R*_f_* 0.42 (EtOAc/Hexane = 1:1). ^1^H NMR (400 MHz, DMSO-*d_6_*) δ 9.02 (s, 1H), 7.45 (d, *J* = 7.2 Hz, 2H), 7.38 (t, *J* = 6.2 Hz, 2H), 7.34–7.22 (m, 6H), 6.64 (s, 1H), 6.53–6.46 (m, 1H), 6.29–6.18 (m, 2H), 3.57 (s, 3H), 3.53 (d, *J* = 15.8 Hz, 1H), 3.49 (d, *J* = 15.8 Hz, 1H), 3.43 (s, 3H);^13^C{^1^H} NMR (101 MHz, DMSO-*d_6_*) δ 171.3, 157.5, 147.9, 146.6, 140.2, 131.5, 130.6, 129.8, 129.1 (2C), 128.1 (2C), 127.8 (3C), 127.7, 126.1 (2C), 120.5, 112.2, 111.2, 88.2, 55.4, 55.0, 30.2; FTIR, *v_max_*: 3527, 3285, 1695, 1672, 1510, 1451, 1260, 1232, 1145, 1029 cm^−1^; HRMS (ESI TOF) *m*/*z*: calculated for C_25_H_23_N_1_Na_1_O_4_ [M + Na]^+^: 424.1519, found 424.1512 (1.8 ppm).

5-Hydroxy-4-(2-hydroxyethyl)-3,5-diphenyl-1H-pyrrol-2(5H)-one (**4ac**-(2-OHCH_2_CH_2_): this compound was prepared employing 4-oxo-2,4-diphenylbutanenitrile **2ac** [1] (235 mg, 1.00 mmol) and d-glucose (189 mg, 1.05 mmol) in a yield of 103 mg (0.35 mmol, 35%). Purification was performed by column chromatography (EtOAc/Hexane = 1:1). The titled compound was obtained as a white solid, m.p. 181.0–182.4 °C, R*_f_* 0.28 (EtOAc/Hexane = 1:1). ^1^H NMR (400 MHz, DMSO-*d_6_*) δ 8.95 (s, 1H), 7.67–7.27 (m, 10H), 6.59 (s, 1H), 4.61 (t, *J* = 5.4 Hz, 1H), 3.36–3.24 (m, 1H), 3.13–2.99 (m, 1H), 2.49–2.37 (m, 1H), 2.20 (ddd, *J* = 12.9, 9.8, 5.7 Hz, 1H);^13^C{^1^H} NMR (101 MHz, DMSO-*d_6_*) δ 171.0, 155.9, 140.4, 131.5, 130.9, 129.1 (2C), 128.3 (2C), 128.1 (2C), 127.9 (2C), 125.8 (2C), 88.0, 58.9, 29.8; FTIR, *v_max_*: 3380, 3169, 1680, 1495, 1447, 1419, 1242, 1191, 1121, 1049, 1033 cm^−1^; HRMS (ESI TOF) *m*/*z*: calculated for C_18_H_17_N_1_Na_1_O_3_ [M + Na]^+^: 318.1101, found 318.1094 (2.0 ppm).

4-Benzyl-5-hydroxy-5-phenyl-3-(p-tolyl)-1H-pyrrol-2(5H)-one (**4ad-a**): this compound was prepared employing 4-oxo-4-phenyl-2-(p-tolyl)butanenitrile **2ad** [1] (249 mg, 1.00 mmol) and benzaldehyde **3a** (111 mg, 1.05 mmol) in a yield of 320 mg (0.9 mmol, 90%). Purification was performed by column chromatography (EtOAc/Hexane = 2:3). The titled compound was obtained as a white amorphous solid, m.p. 171.0–172.1 °C, R*_f_* 0.23 (EtOAc/Hexane = 2:3). ^1^H NMR (400 MHz, DMSO-*d_6_*) δ 9.01 (s, 1H), 7.45–7.38 (m, 2H), 7.32–7.19 (m, 5H), 7.11 (d, *J* = 8.0 Hz, 2H), 6.95 (dd, *J* = 5.0, 1.9 Hz, 3H), 6.77 (dd, *J* = 7.0, 2.5 Hz, 2H), 6.58 (s, 1H), 3.65–3.51 (m, 2H), 2.26 (s, 3H);^13^C{^1^H} NMR (101 MHz, DMSO-*d_6_*) δ 171.3, 156.3, 140.3, 137.6, 137.0, 130.7, 128.9 (2C), 128.5 (2C), 128.4, 128.3 (2C), 128.1 (2C), 127.7 (3C), 126.1 (2C), 125.5, 88.1, 30.8, 20.9; FTIR, *v_max_*: 3432, 1702, 1600, 1459, 1381, 1286, 1200, 1173, 1063 cm^−1^; HRMS (ESI TOF) *m*/*z*: calculated for C_24_H_21_N_1_Na_1_O_2_ [M + Na]^+^: 378.1464, found 378.1469 (−1.2 ppm).

4-Benzyl-3-(4-ethylphenyl)-5-hydroxy-5-phenyl-1H-pyrrol-2(5H)-one (**4ae-a**): this compound was prepared employing 2-(4-ethylphenyl)-4-oxo-4-phenylbutanenitrile **2ae** [1] (263 mg, 1.00 mmol) and benzaldehyde **3a** (111 mg, 1.05 mmol) in a yield of 339 mg (0.92 mmol, 92%). Purification was performed by column chromatography (EtOAc/Hexane = 1:1). The titled compound was obtained as a yellowish amorphous solid, m.p. 62.6–64.1 °C, R*_f_* 0.37 (EtOAc/Hexane = 1:1). ^1^H NMR (400 MHz, DMSO-*d_6_*) δ 9.01 (s, 1H), 7.41 (d, *J* = 7.0 Hz, 2H), 7.33–7.21 (m, 5H), 7.13 (d, *J* = 8.6 Hz, 2H), 6.95 (dd, *J* = 5.1, 1.9 Hz, 3H), 6.81–6.72 (m, 2H), 6.58 (s, 1H), 3.64–3.50 (m, 2H), 2.56 (q, *J* = 7.6 Hz, 2H), 1.14 (t, *J* = 7.6 Hz, 3H);^13^C{^1^H} NMR (101 MHz, DMSO-*d_6_*) δ 171.7, 156.7, 143.8, 140.7, 138.0, 131.2, 129.4 (2C), 129.1, 128.8 (2C), 128.5 (2C), 128.1 (3C), 127.7 (2C), 126.5 (2C), 126.0, 88.6, 31.3, 28.4, 16.0; FTIR, *v_max_*: 1698, 1602, 1453, 1254, 1171, 1097, 1057, 964 cm^−1^; HRMS (ESI TOF) *m*/*z*: calculated for C_25_H_23_N_1_Na_1_O_2_ [M + Na]^+^: 392.1621, found 392.1628 (−1.8 ppm).

4-Benzyl-5-hydroxy-3-(4-methoxyphenyl)-5-phenyl-1H-pyrrol-2(5H)-one (**4af-a**): this compound was prepared employing 2-(4-methoxyphenyl)-4-oxo-4-phenylbutanenitrile **2af** [1] (264 mg, 1.00 mmol) and benzaldehyde **3a** (111 mg, 1.05 mmol) in a yield of 288 mg (0.78 mmol, 78%). Purification was performed by column chromatography (EtOAc/Hexane = 2:3). The titled compound was obtained as a yellowish amorphous solid, m.p. 70.7–72.3 °C, R*_f_* 0.23 (EtOAc/Hexane = 2:3). ^1^H NMR (400 MHz, DMSO-*d_6_*) δ 9.00 (s, 1H), 7.42 (d, *J* = 7.0 Hz, 2H), 7.36 (d, *J* = 8.8 Hz, 2H), 7.31–7.21 (m, 3H), 7.02–6.90 (m, 3H), 6.85 (d, *J* = 8.8 Hz, 2H), 6.79 (dd, *J* = 7.2, 2.3 Hz, 2H), 6.55 (s, 1H), 3.72 (s, 3H), 3.66–3.51 (m, 2H);^13^C{^1^H} NMR (101 MHz, DMSO-*d_6_*) δ 171.4, 158.8, 155.4, 140.3, 137.6, 130.2 (3C), 128.3 (2C), 128.1 (2C), 127.7 (3C), 126.1 (2C), 125.6, 123.5, 113.3 (2C), 88.1, 55.1, 30.9; FTIR, *v_max_*: 1694, 1604, 1509, 1451, 1302, 1242, 1171, 1105, 1029 cm^−1^; HRMS (ESI TOF) *m*/*z*: calculated for C_24_H_21_N_1_Na_1_O_3_ [M + Na]^+^: 394.1414, found 394.1408 (1.4 ppm).

4-Benzyl-3-(3-chlorophenyl)-5-hydroxy-5-phenyl-1H-pyrrol-2(5H)-one (**4ag-a**): this compound was prepared employing 2-(3-chlorophenyl)-4-oxo-4-phenylbutanenitrile **2ag** [1] (269 mg, 1.00 mmol) and benzaldehyde **3a** (111 mg, 1.05 mmol) in a yield of 323 mg (0.86 mmol, 86%). Purification was performed by column chromatography (EtOAc/Hexane = 1:1). The titled compound was obtained as a yellowish amorphous solid, m.p. 60.9–62.8 °C, R*_f_* 0.4 (EtOAc/Hexane = 1:1). ^1^H NMR (400 MHz, DMSO-*d_6_*) δ 9.14 (s, 1H), 7.48 (d, *J* = 7.0 Hz, 2H), 7.37–7.30 (m, 3H), 7.30–7.24 (m, 4H), 7.00–6.89 (m, 3H), 6.77 (dd, *J* = 7.3, 2.2 Hz, 2H), 6.70 (s, 1H), 3.66–3.49 (m, 2H);^13^C{^1^H} NMR (101 MHz, DMSO-*d_6_*) δ 170.7, 158.6, 139.8, 137.1, 133.3, 132.3, 129.5, 129.2, 128.8, 128.5 (2C), 128.2 (2C), 127.9, 127.7 (2C), 127.6, 127.5, 126.1 (2C), 125.7, 88.2, 30.9; FTIR, *v_max_*: 1698, 1602, 1495, 1451, 1274, 1103, 970 cm^−1^; HRMS (ESI TOF) *m*/*z*: calculated for C_23_H_18_ClN_1_Na_1_O_2_ [M + Na]^+^: 398.0918, found 398.0922 (−0.9 ppm).

4-Benzyl-3-(2-fluorophenyl)-5-hydroxy-5-phenyl-1H-pyrrol-2(5H)-one (**4ah-a**): this compound was prepared employing 2-(2-fluorophenyl)-4-oxo-4-phenylbutanenitrile **2ah** [1] (253 mg, 1.00 mmol) and benzaldehyde **3a** (111 mg, 1.05 mmol) in a yield of 335 mg (0.93 mmol, 93%). Purification was performed by column chromatography (EtOAc/Hexane = 2:3). The titled compound was obtained as a yellowish amorphous solid, m.p. 61.2–63.5 °C, R*_f_* 0.23 (EtOAc/Hexane = 2:3). ^1^H NMR (400 MHz, DMSO-*d_6_*) δ 9.05 (s, 1H), 7.48 (d, *J* = 7.1 Hz, 2H), 7.36 (t, *J* = 7.6 Hz, 2H), 7.32–7.25 (m, 2H), 7.14 (t, *J* = 6.5 Hz, 1H), 7.06 (t, *J* = 8.1 Hz, 2H), 6.90–6.85 (m, 3H), 6.78 (s, 1H), 6.71–6.64 (m, 2H), 3.55–3.26 (m, 2H);^13^C{^1^H} NMR (101 MHz, DMSO-*d_6_*) δ 170.5, 160.3, 159.6 (d, *J* = 245.8 Hz), 140.0, 136.7, 131.4 (d, *J* = 3.7 Hz), 130.0 (d, *J* = 8.4 Hz), 128.5 (2C), 128.3 (2C), 127.9, 127.5 (2C), 126.6, 125.9 (2C), 125.6, 123.7 (d, *J* = 3.3 Hz), 119.3 (d, *J* = 16.5 Hz), 115.1 (d, *J* = 21.6 Hz), 88.6, 31.3 (d, *J* = 2.1 Hz); FTIR, *v_max_*: 1698, 1495, 1447, 1216, 1103, 1067, 964 cm^−1^; HRMS (ESI TOF) *m*/*z*: calculated for C_23_H_18_FN_1_Na_1_O_2_ [M + Na]^+^: 382.1214, found 382.1224 (−2.7 ppm).

4-Benzyl-3-(4-fluorophenyl)-5-hydroxy-5-phenyl-1H-pyrrol-2(5H)-one (**4ai-a**): this compound was prepared employing 2-(4-fluorophenyl)-4-oxo-4-phenylbutanenitrile **2ai** [1] (253 mg, 1.00 mmol) and benzaldehyde **3a** (111 mg, 1.05 mmol) in a yield of 331 mg (0.92 mmol, 92%). Purification was performed by column chromatography (EtOAc/Hexane = 2:3). The titled compound was obtained as a yellowish amorphous solid, m.p. 58.5–60.8 °C, R*_f_* 0.29 (EtOAc/Hexane = 2:3). ^1^H NMR (400 MHz, DMSO-*d_6_*) δ 9.08 (s, 1H), 7.46 (d, *J* = 6.9 Hz, 2H), 7.39 (m, 2H), 7.34–7.23 (m, 3H), 7.09 (t, *J* = 9.0 Hz, 2H), 6.94 (dd, *J* = 5.1, 1.9 Hz, 3H), 6.82–6.72 (m, 2H), 6.66 (s, 1H), 3.65–3.51 (m, 2H);^13^C{^1^H} NMR (101 MHz, DMSO-*d_6_*) δ 171.1, 161.5 (d, *J* = 244.7 Hz), 157.2, 140.1, 137.2, 131.1 (2C, d, *J* = 8.4 Hz), 129.6, 128.5 (2C), 128.2 (2C), 127.8, 127.7 (2C), 127.6 (d, *J* = 3.2 Hz), 126.1 (2C), 125.6, 114.7 (2C, d, *J* = 21.3 Hz), 88.2, 30.8 FTIR, *v_max_*: 1702, 1598, 1505, 1451, 1232, 1161, 1063 cm^−1^; HRMS (ESI TOF) *m*/*z*: calculated for C_23_H_18_FN_1_Na_1_O_2_ [M + Na]^+^: 382.1214, found 382.1225 (−3.0 ppm).

4-Benzyl-3-(4-(dimethylamino)phenyl)-5-hydroxy-5-phenyl-1H-pyrrol-2(5H)-one (**4aj-a**): this compound was prepared employing 2-(4-(dimethylamino)phenyl)-4-oxo-4-phenylbutanenitrile **2aj** [1] (278 mg, 1.00 mmol) and benzaldehyde **3a** (111 mg, 1.05 mmol) in a yield of 277 mg (0.72 mmol, 72%). Purification was performed by column chromatography (EtOAc/Hexane = 1:1). The titled compound was obtained as an orange amorphous solid, m.p. 189.3–191.0 °C, R*_f_* 0.17 (EtOAc/Hexane = 1:1). ^1^H NMR (400 MHz, DMSO-*d_6_*) δ 8.93 (s, 1H), 7.39 (d, *J* = 6.8 Hz, 2H), 7.34 (d, *J* = 8.9 Hz, 2H), 7.27–7.18 (m, 3H), 7.03–6.91 (m, 3H), 6.82 (d, *J* = 5.4 Hz, 2H), 6.62 (d, *J* = 9.2 Hz, 2H), 6.45 (s, 1H), 3.68–3.51 (m, 2H), 2.87 (s, 6H);^13^C{^1^H} NMR (101 MHz, DMSO-*d_6_*) δ 171.8, 153.2, 149.7, 140.7, 138.0, 130.4, 129.6 (2C), 128.2 (2C), 128.0 (2C), 127.7 (2C), 127.5, 126.0 (2C), 125.5, 118.7, 111.4 (2C), 88.0, 39.9 (2C), 31.0; FTIR, *v_max_*: 3273, 1668, 1608, 1525, 1447, 1372, 1276, 1204, 1101, 1053, 998 cm^−1^; HRMS (ESI TOF) *m*/*z*: calculated for C_23_H_18_FN_1_Na_1_O_2_ [M+H]^+^: 385.1911, found 385.1905 (1.5 ppm).

3-(Benzo[d][1,3]dioxol-5-yl)-4-benzyl-5-hydroxy-5-phenyl-1H-pyrrol-2(5H)-one (**4ak-a**): this compound was prepared employing (*E*)-3-(benzo[d][1,3]dioxol-5-yl)-1-phenylprop-2-en-1-one **2ak** [1] (252 mg, 1.00 mmol) and benzaldehyde **3a** (111 mg, 1.05 mmol) in a yield of 328 mg (0.85 mmol, 85%). Purification was performed by column chromatography (EtOAc/Hexane = 1:1). The titled compound was obtained as a white amorphous solid, m.p. 152.6–153.7 °C, R*_f_* 0.23 (EtOAc/Hexane = 1:1). ^1^H NMR (400 MHz, DMSO-*d_6_*) δ 9.03 (s, 1H), 7.42 (d, *J* = 7.0 Hz, 2H), 7.35–7.18 (m, 3H), 7.01–6.95 (m, 3H), 6.92–6.87 (m, 2H), 6.82 (d, *J* = 8.4 Hz, 1H), 6.79 (dd, *J* = 7.3, 2.2 Hz, 2H), 6.59 (s, 1H), 5.97 (s, 2H), 3.57 (s, 2H);^13^C{^1^H} NMR (101 MHz, DMSO-*d_6_*) δ 171.2, 156.0, 146.7 (2C), 140.2, 137.5, 130.21, 128.4 (2C), 128.1 (2C), 127.7 (3C), 126.1 (2C), 125.6, 124.9, 122.9, 109.2, 107.9, 101.0, 88.1, 30.8; FTIR, *v_max_*: 1696, 1602, 1499, 1455, 1439, 1336, 1254, 1097, 1065, 1041 cm^−1^; HRMS (ESI TOF) *m*/*z*: calculated for C_24_H_19_N_1_Na_1_O_4_ [M + Na]^+^: 408.1215, found 408.1206 (−2.1 ppm).

4-Benzyl-5-hydroxy-3-phenyl-5-(p-tolyl)-1H-pyrrol-2(5H)-one (**4bc-a**): this compound was prepared employing 4-oxo-2-phenyl-4-(*p*-tolyl)butanenitrile **2bc** [1] (249 mg, 1.00 mmol) and benzaldehyde **3a** (111 mg, 1.05 mmol) in a yield of 290 mg (0.82 mmol, 82%). Purification was performed by column chromatography (EtOAc/Hexane = 1:1). The titled compound was obtained as a white amorphous solid, m.p. 192.1–192.9 °C, R*_f_* 0.31 (EtOAc/Hexane = 1:1). ^1^H NMR (400 MHz, DMSO-*d_6_*) δ 8.99 (s, 1H), 7.39–7.19 (m, 7H), 7.11 (d, *J* = 8.2 Hz, 2H), 6.95 (dd, *J* = 4.9, 2.0 Hz, 3H), 6.84–6.74 (m, 2H), 6.54 (s, 1H), 3.55 (m, 2H), 2.26 (s, 3H);^13^C{^1^H} NMR (101 MHz, DMSO-*d_6_*) δ 171.2, 157.1, 137.5, 137.2, 136.9, 131.3, 130.6, 129.0 (2C), 128.7 (2C), 128.5 (2C), 127.8 (2C), 127.6 (3C), 126.0 (2C), 125.5, 88.2, 30.8, 20.7; FTIR, *v_max_*: 3531, 1710, 1604, 1495, 1366, 1254, 1101, 1051, 1031, 965 cm^−1^; HRMS (ESI TOF) *m*/*z*: calculated for C_24_H_21_N_1_Na_1_O_2_ [M + Na]^+^: 378.1464, found 378.1467 (−0.7 ppm).

4-Benzyl-3-(4-ethylphenyl)-5-hydroxy-5-phenyl-1H-pyrrol-2(5H)-one (**4ce-a**): this compound was prepared employing 2-(4-ethylphenyl)-4-(4-methoxyphenyl)-4-oxobutanenitrile **2ce** (293 mg, 1.00 mmol) and benzaldehyde **3a** (111 mg, 1.05 mmol) in a yield of 308 mg (0.77 mmol, 77%). Purification was performed by column chromatography (EtOAc/Hexane = 1:1). The titled compound was obtained as a yellowish amorphous solid, m.p. 68.8–71.1 °C, R*_f_* 0.29 (EtOAc/Hexane = 1:1). ^1^H NMR (400 MHz, DMSO-*d_6_*) δ 8.94 (s, 1H), 7.30 (m, 4H), 7.12 (d, *J* = 8.1 Hz, 2H), 7.01–6.93 (m, 3H), 6.87–6.74 (m, 4H), 6.49 (s, 1H), 3.70 (s, 3H), 3.63–3.49 (m, 2H), 2.55 (q, *J* = 7.6 Hz, 2H), 1.14 (t, *J* = 7.6 Hz, 3H);^13^C{^1^H} NMR (101 MHz, DMSO-*d_6_*) δ 171.2, 158.9, 156.5, 143.3, 137.7, 132.0, 130.4, 128.9 (2C), 128.7, 128.4 (2C), 127.7 (2C), 127.3 (2C), 127.2 (2C), 125.5, 113.4 (2C), 88.0, 55.1, 30.8, 28.0, 15.6; FTIR, *v_max_*: 1698, 1602, 1511, 1451, 1244, 1171, 1025, 964 cm^−1^; HRMS (ESI TOF) *m*/*z*: calculated for C_26_H_25_N_1_Na_1_O_3_ [M + Na]^+^: 422.1727, found 422.1730 (−0.9 ppm).

4-Benzyl-5-hydroxy-3-(4-ethylphenyl)-5-(5,6,7,8-tetrahydronaphthalen-2-yl)-1H-pyrrol-2(5H)-one (**4de-a**): this compound was prepared employing 2-(4-ethylphenyl)-4-oxo-4-(5,6,7,8-tetrahydronaphthalen-2-yl)butanenitrile **2de** (317 mg, 1.00 mmol) and benzaldehyde **3a** (111 mg, 1.05 mmol) in a yield of 262 mg (0.62 mmol, 62%). Purification was performed by column chromatography (EtOAc/Hexane = 1:1). The titled compound was obtained as a yellowish amorphous solid, m.p. 79.1–80.6 °C, R*_f_* 0.4 (EtOAc/Hexane = 1:1). ^1^H NMR (400 MHz, DMSO-*d_6_*) δ 8.90 (s, 1H), 7.31 (d, *J* = 8.2 Hz, 2H), 7.13 (d, *J* = 8.4 Hz, 2H), 7.08 (dd, *J* = 7.9, 2.1 Hz, 1H), 7.02 (s, 1H), 6.98–6.90 (m, 4H), 6.79 (dd, *J* = 7.0, 2.6 Hz, 2H), 6.45 (s, 1H), 3.56 (s, 2H), 2.65–2.51 (m, 6H), 1.67 (s, 4H), 1.14 (t, *J* = 7.6 Hz, 3H);^13^C{^1^H} NMR (101 MHz, DMSO-*d_6_*) δ 171.7, 156.9, 143.7, 138.1, 137.5, 136.4 (2C), 130.9, 129.6 (2C), 129.2, 129.0, 128.8 (2C), 128.0 (2C), 127.7 (2C), 127.1, 125.8, 123.5, 88.5, 31.4, 29.4, 29.0, 28.4, 23.2 (2C), 16.0; FTIR, *v_max_*: 1698, 1505, 1457, 1244, 1155, 1057, 968 cm^−1^; HRMS (ESI TOF) *m*/*z*: calculated for C_29_H_29_N_1_Na_1_O_2_ [M + Na]^+^: 446.2090, found 446.2097 (−1.4 ppm).

4-Benzyl-5-hydroxy-3-(4-methoxyphenyl)-5-(5,6,7,8-tetrahydronaphthalen-2-yl)-1H-pyrrol-2(5H)-one (**4df-a**): this compound was prepared employing 2-(4-methoxyphenyl)-4-oxo-4-(5,6,7,8-tetrahydronaphthalen-2-yl)butanenitrile **2df** (319 mg, 1.00 mmol) and benzaldehyde **3a** (111 mg, 1.05 mmol) in a yield of 249 mg (0.59 mmol, 59%). Purification was performed by column chromatography (EtOAc/Hexane = 1:1). The titled compound was obtained as a yellowish amorphous solid, m.p. 76.2–78.0 °C, R*_f_* 0.23 (EtOAc/Hexane = 1:1). ^1^H NMR (400 MHz, DMSO-*d_6_*) δ 8.90 (s, 1H), 7.36 (d, *J* = 8.9 Hz, 2H), 7.08 (dd, *J* = 7.9, 2.1 Hz, 1H), 7.02 (s, 1H), 7.00–6.95 (m, 3H), 6.92 (d, *J* = 8.1 Hz, 1H), 6.85 (d, *J* = 8.9 Hz, 2H), 6.83–6.80 (m, 2H), 6.42 (s, 1H), 3.72 (s, 3H), 3.57 (s, 2H), 2.61 (d, *J* = 14.5 Hz, 4H), 1.67 (t, *J* = 3.2 Hz, 4H);^13^C{^1^H} NMR (101 MHz, DMSO-*d_6_*) δ 171.4, 158.7, 155.6, 137.7, 137.2, 136.0, 135.9, 130.2 (2C), 129.8, 128.6, 128.4 (2C), 127.6 (2C), 126.7, 125.4, 123.6, 123.1, 113.3 (2C), 88.0, 55.1, 31.0, 28.9, 28.5, 22.8 (2C); FTIR, *v_max_*: 1698, 1606, 1511, 1455, 1246, 1175, 1059, 966 cm^−1^; HRMS (ESI TOF) *m*/*z*: calculated for C_28_H_27_N_1_Na_1_O_3_ [M + Na]^+^: 448.1883, found 448.1891 (−1.8 ppm).

## 4. Conclusions

An easy, low-cost synthetic pathway to a variety of novel, fully substituted 5-hydroxy 3-pyrrolin-2-ones was developed. These compounds are of interest to medicinal and organic synthetic chemists as they are analogous (structurally similar) to many naturally occurring bioactive γ-hydroxy butyrolactams. On the other hand, the richness and diversity of functional groups make those 3-pyrrolin-2-one derivatives a convenient starting point for the synthesis of other, more complex heterocyclic systems possessing, potentially, interesting pharmacological properties as well.

## Figures and Tables

**Table 1:** Screening of reaction parameters for synthesis of the γ-hydroxy butyrolactams **4ac-i**.

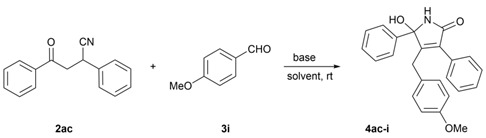				
#	Base, eq	Solvent, M/L	Time, h	Yield ^a^, %
1	MeONa, 4	MeOH, 0.5	4	89
2	MeONa, 2	MeOH, 0.5	8	85
3	MeONa, 1	MeOH, 0.5	28	81
4	MeONa, 4	MeOH, 1	1.5	84
5	Et_3_N, 4	MeOH, 1	24	NR
6	DBU, 4	MeOH, 1	24	71
7	KOH, 4	MeOH, 1	1.5	67
8	MeONa, 4	MeCN, 1	1.5	0
9	MeONa, 4	DMF, 1	2	0
10	MeONa, 4	EtOH 96%, 1	1.5	65
11	MeONa, 4	EtOH 96%, 0.5	4	75
12	MeONa, 4	DMSO, 1	2	0

^a^ The reactions were performed on 0.5 mmol scales and equimolar quantities of reactants. Isolated yields of purified materials are provided.

**Table 2:** The reaction scope and yields ^a^ of obtained 3,4,5-trisubstituted γ-hydroxy lactams **4**.

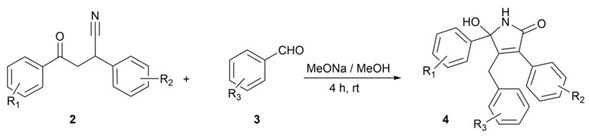								
3-cyanoketone 2			aldehyde 3		γ-lactams 4			
R_1_	R_2_	#	R_3_	#	#	%	#	% ^a^
H	4-Cl	**2ab**	H	**3a**	**4ab-a**	69	**4ak-a**	85
H	H	**2ac**	4-Et	**3b**	**4ac-b**	74	**4ce-a**	77
4-Me	H	**2bc**	4-Me	**3c**	**4bc-a**	82	**4df-a**	59
H	4-Me	**2ad**	3-Cl	**3d**	**4ad-a**	90	**4de-a**	62
H	4-Et	**2ae**	2-Cl	**3e**	**4ae-a**	92	**4ac-i**	92
H	4-OMe	**2af**	4-Cl	**3f**	**4af-a**	78	**4ac-j**	71
H	3-Cl	**2ag**	2-F	**3g**	**4ac-c**	86	**4ac-k**	79
H	2-F	**2ah**	4-F	**3h**	**4ac-d**	92	**4ac-l**	91
H	4-F	**2ai**	4-OMe	**3i**	**4ac-e**	77	**4ac-m**	84
H	4-(NMe)_2_	**2aj**	2-Py	**3j**	**4ag-a**	86	**4ac-a**	85
H	3,4-(OCH_2_O)	**2ak**	3-Py	**3k**	**4ah-a**	93	**4ac-n**	82
4-OMe	4-Et	**2ce**	4-Py	**3l**	**4ai-a**	92	**4ac-o**	79
tetralin	4-OMe	**2df**	2-thiophene	**3m**	**4aj-a**	72	**4ac-p**	85
tetralin	4-Et	**2de**	4-(*i*-Pr)	**3n**	**4ac-f**	88		
			2-Me	**3o**	**4ac-g**	92		
			3,4-(OMe)_2_	**3p**	**4ac-h**	91		

^a^ Isolated yields of purified materials are provided.

## Data Availability

The supporting information includes NMR spectral charts.

## Supplementary Materials

The following supporting information can be downloaded at: https://www.mdpi.com/article/10.3390/ijms241210213/s1.
