# Supplementary material for: A New, Convenient Way to Fully Substituted α,β-Unsaturated γ-Hydroxy Butyrolactams
Source: Int J Mol Sci. 2023 Jun 16;24(12):10213. doi: 10.3390/ijms241210213 (PMC10299564; doi:10.3390/ijms241210213)
Supplement: Supplementary file 1 [file ijms-24-10213-s001.zip › ijms-2454509-supplementary.pdf]

Electronic Supplementary Material (ESI)

A new, convenient way to fully substituted α,β-unsaturated γ-hydroxy butyrolactams

Alexander V. Aksenov *, Dmitrii A. Aksenov, Igor A. Kurenkov, Alexander V. Leontiev, and Nicolai A. Aksenov

Department of Chemistry, North Caucasus Federal University, 1a Pushkin St., 355009 Stavropol, Russia
***** *Correspondence: aaksenov@ncfu.ru (A.V.A.)*

Supporting Information

NMR Spectral Charts……………………………………………………………………………………………...…S2
^{1}H and ^{13}C NMR spectral charts for chalcones **5**……………………………………………………………………………….S2
^{1}H and ^{13}C NMR spectral charts for 2,4-diaryl-4-oxobutanenitriles **2**…...…………………………………………………….S8
^{1}H and ^{13}C NMR spectral charts for 4-benzyl-5-hydroxy-3,5-diaryl-1*H*-pyrrol-2(5*H*)-ones **4** ………………………………S14

^{1}H and ^{13}C NMR spectral charts for chalcones 5

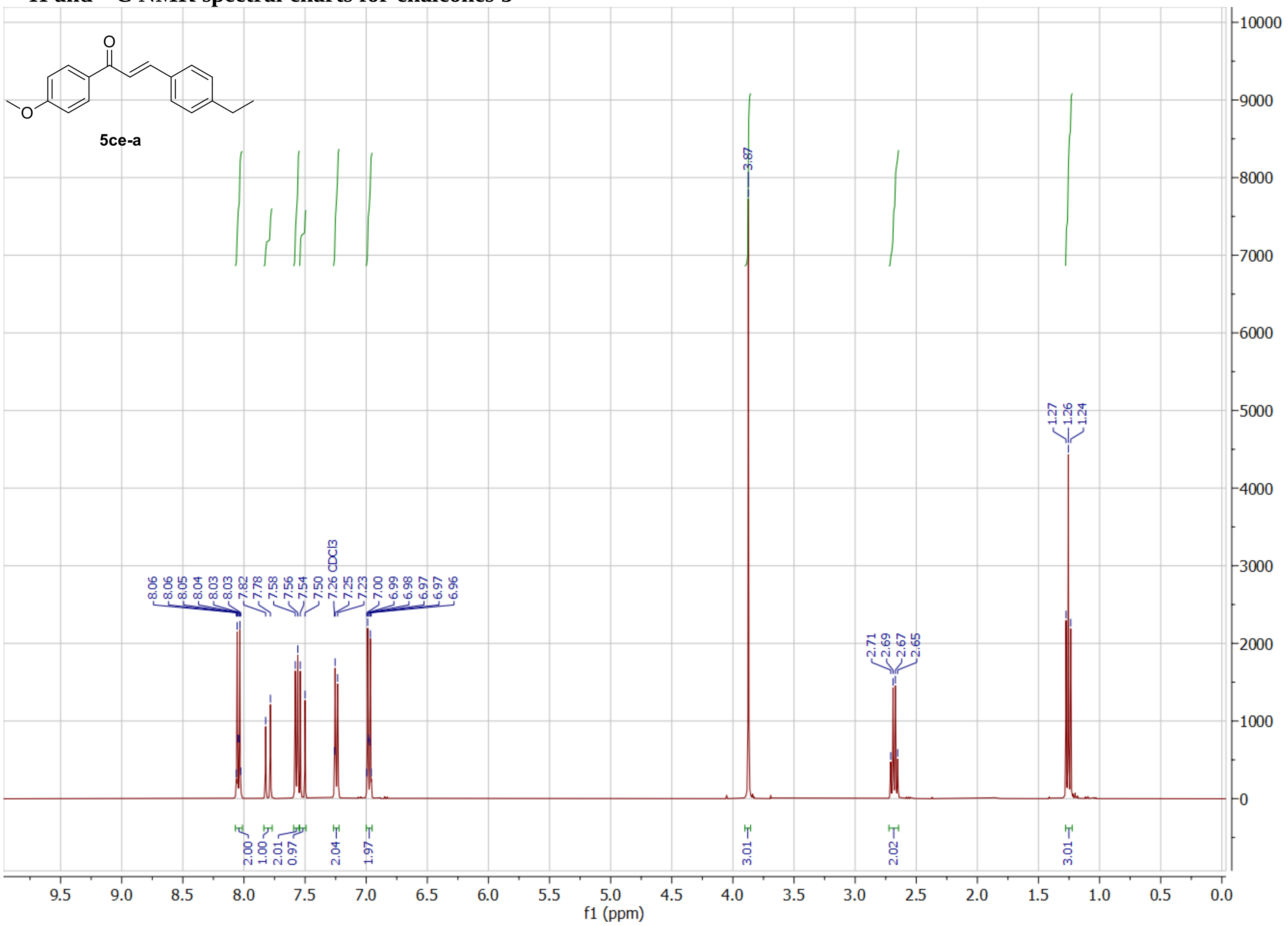

Figure S1. ^{1}H NMR spectrum of **5ce-a** in $CDCl_3$ (400 MHz)

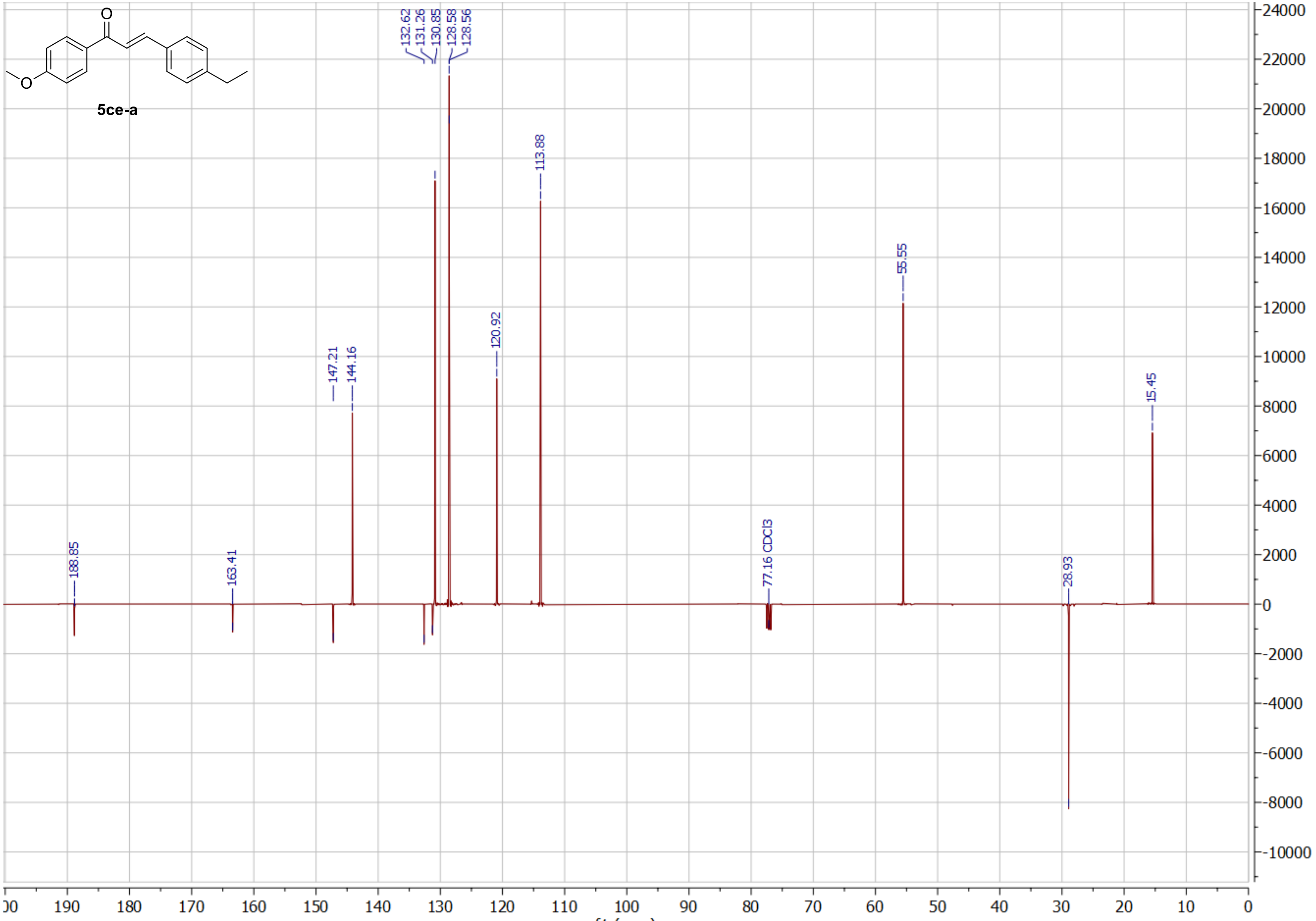

Figure S2. ^{13}C NMR spectrum of **5ce-a** in $CDCl_3$ (101 MHz)

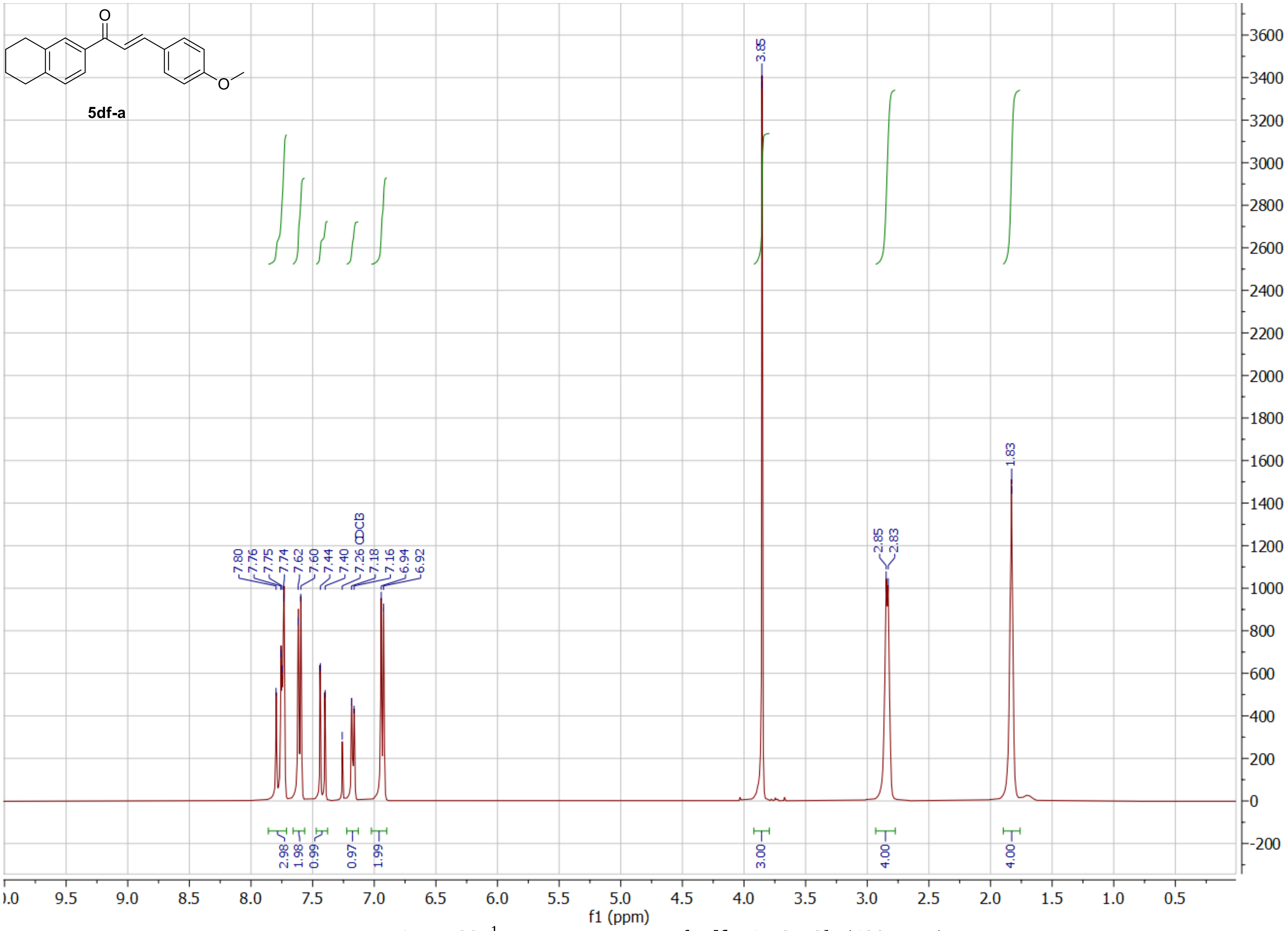

Figure S3. ^{1}H NMR spectrum of **5df-a** in $CDCl_3$ (400 MHz)

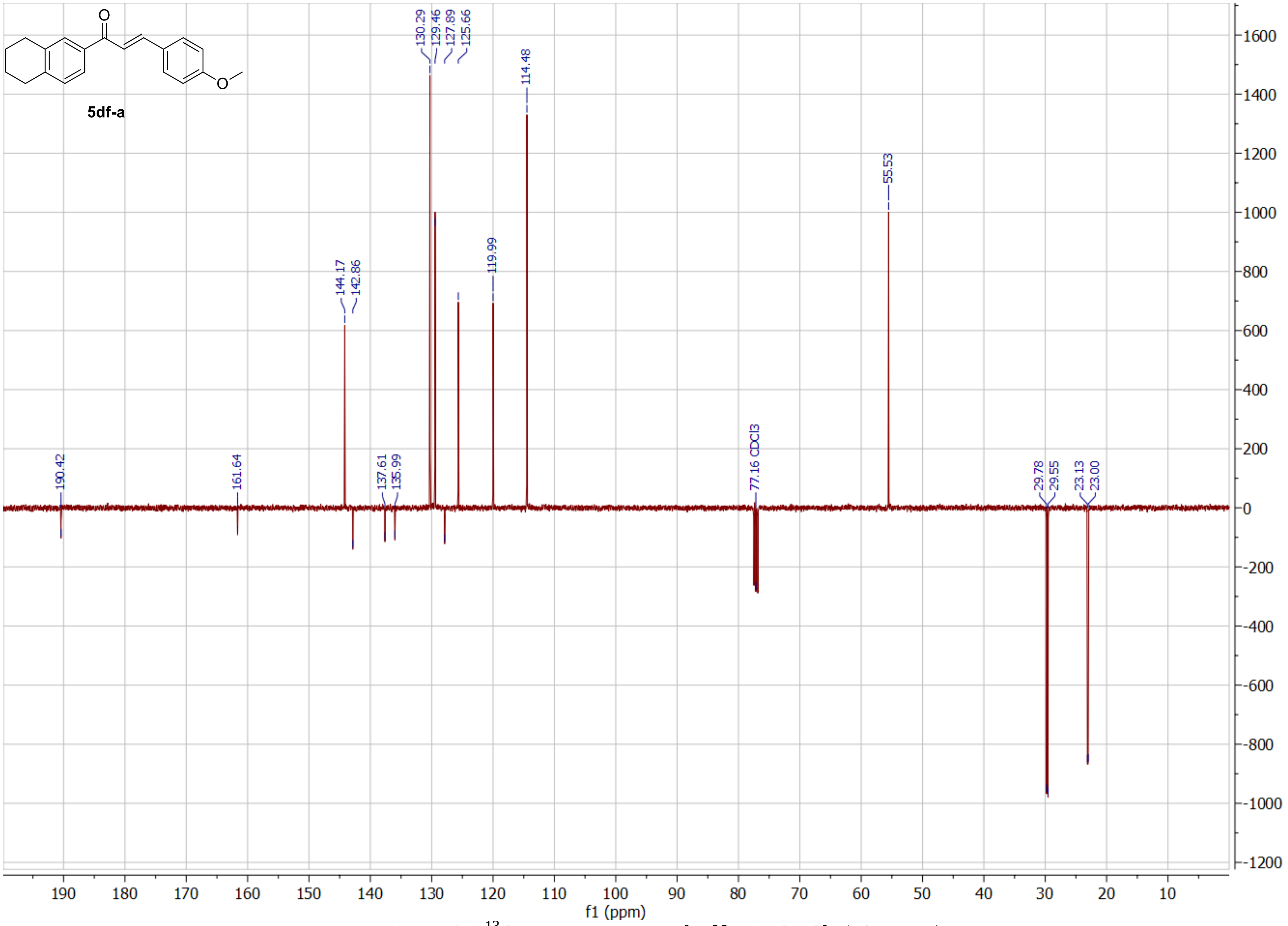

Figure S4. ^{13}C NMR spectrum of **5df-a** in $CDCl_3$ (101 MHz)

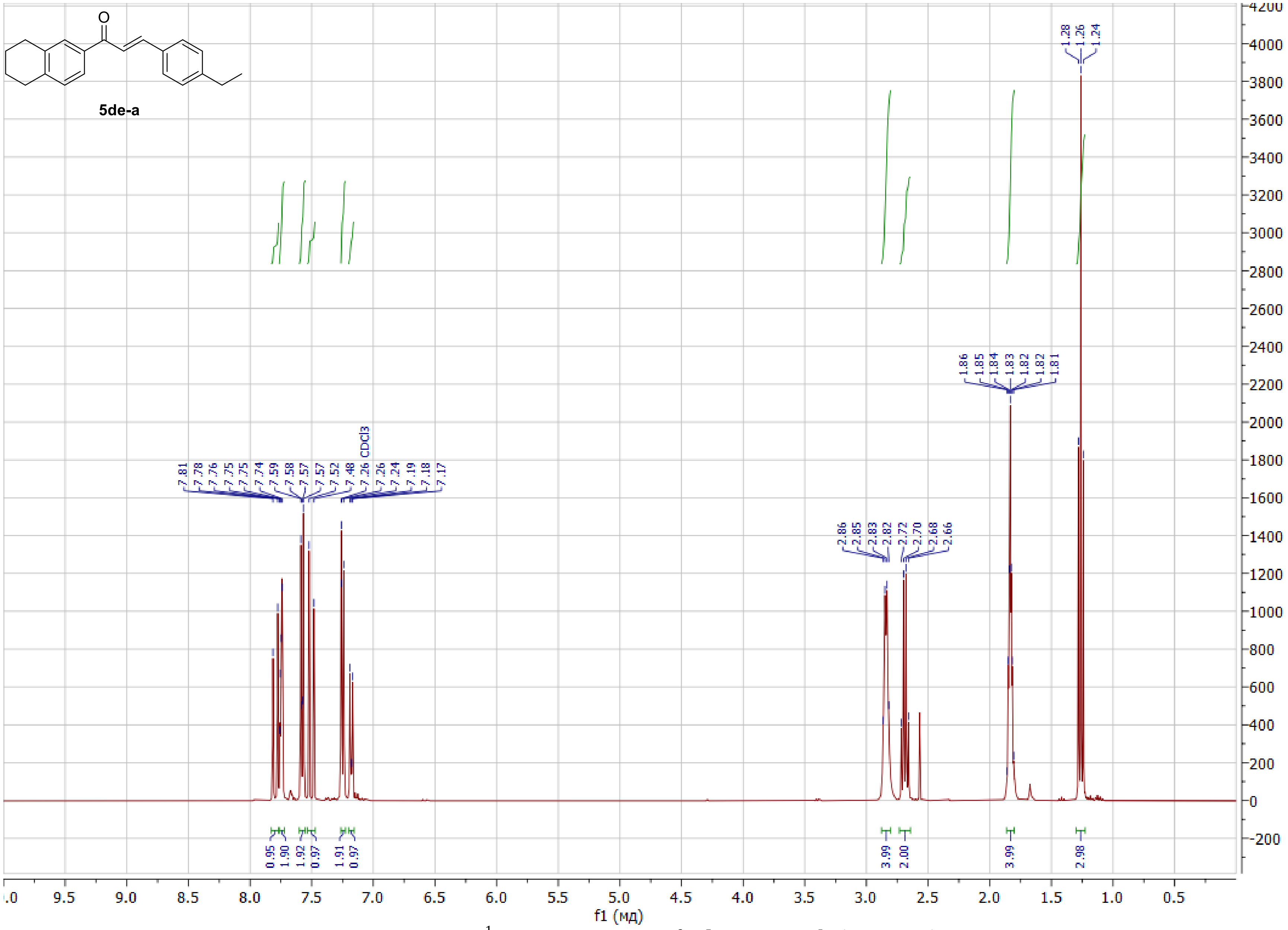

Figure S5. ^{1}H NMR spectrum of **5de-a** in $CDCl_3$ (400 MHz)

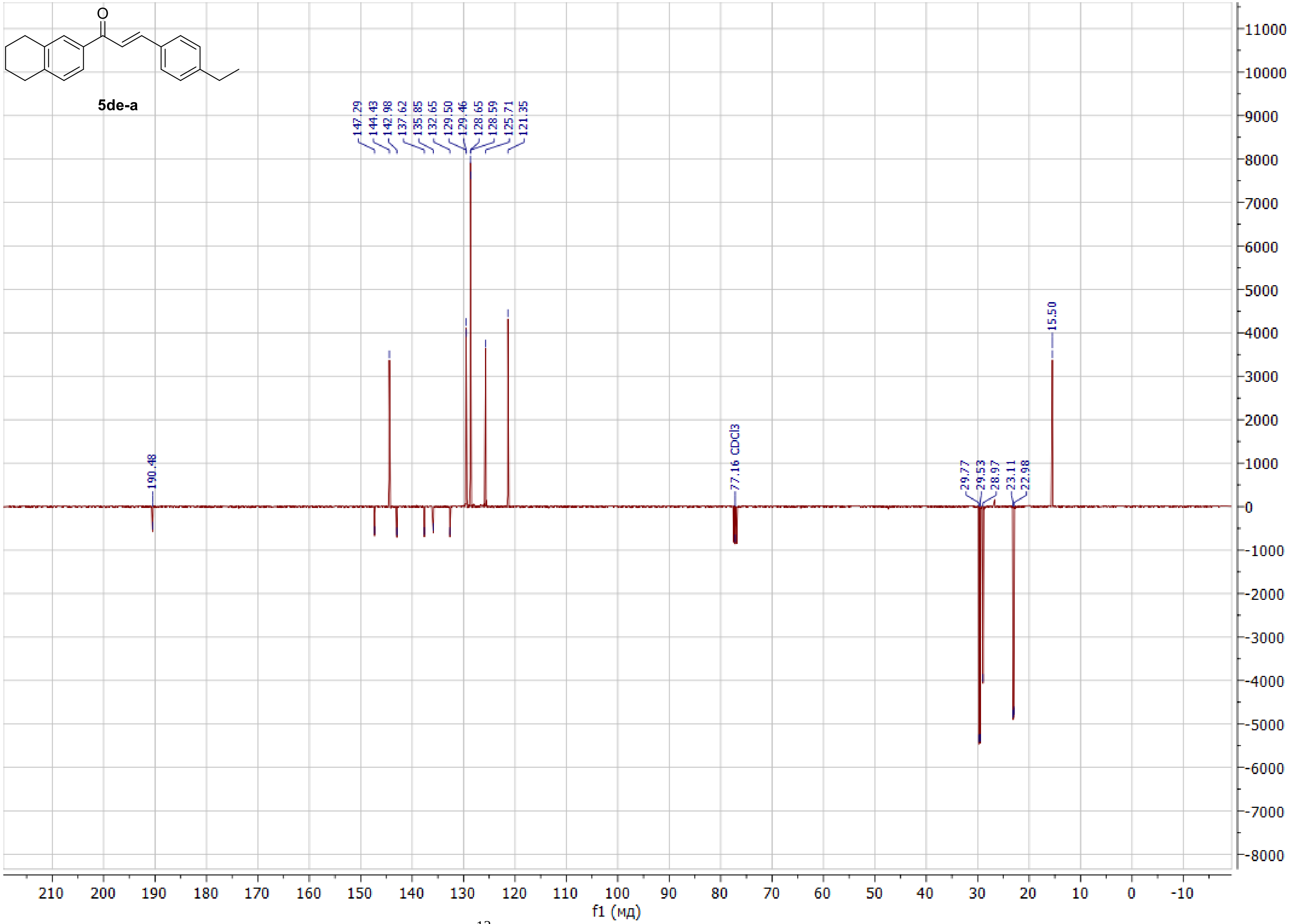

Figure S6. ^{13}C NMR spectrum of **5de-a** in $CDCl_3$ (101 MHz)

^{1}H and ^{13}C NMR spectral charts for 2,4-diaryl-4-oxobutanenitriles 2

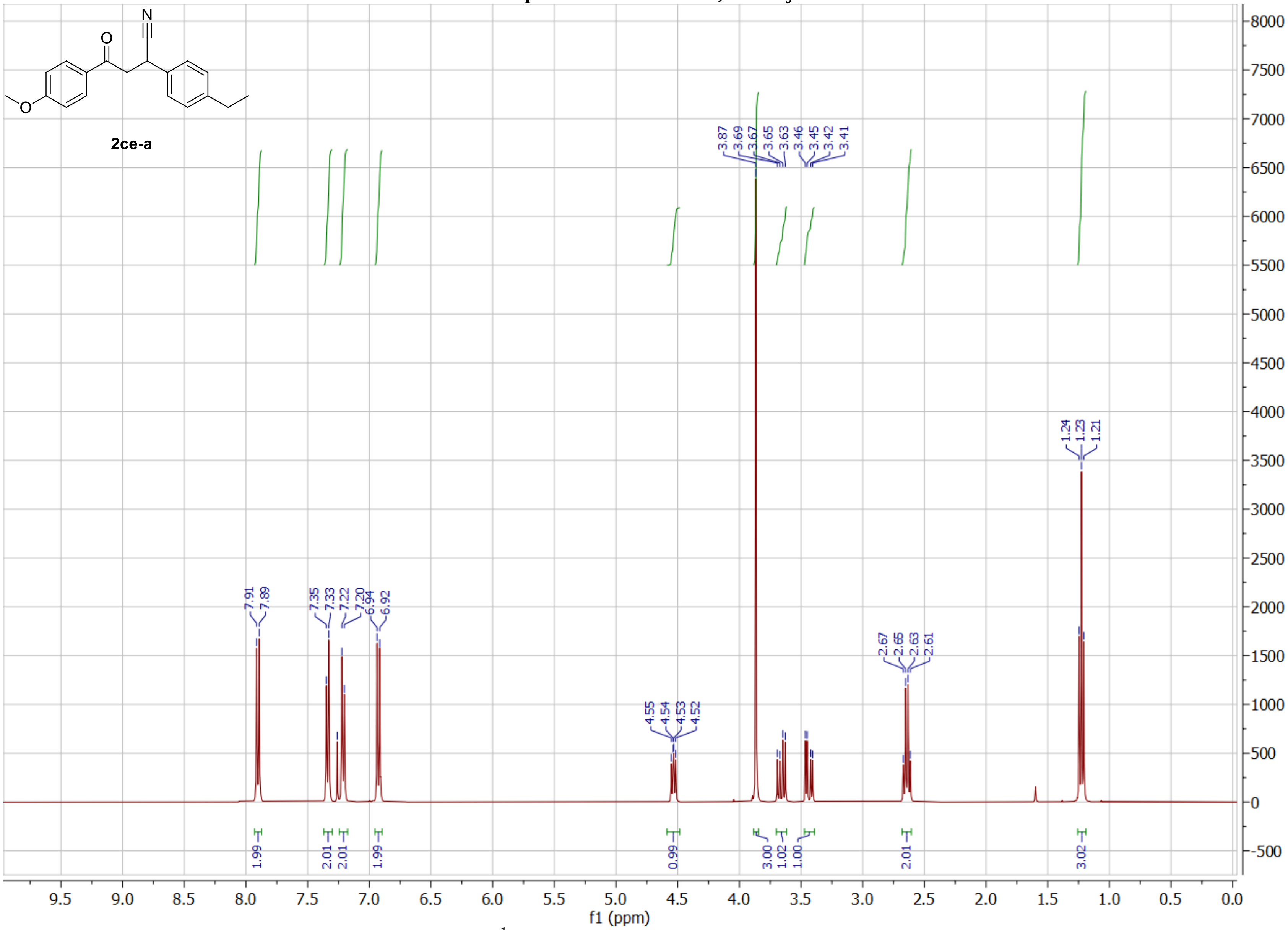

Figure S7. ^{1}H NMR spectrum of **2ce-a** in $CDCl_3$ (400 MHz)

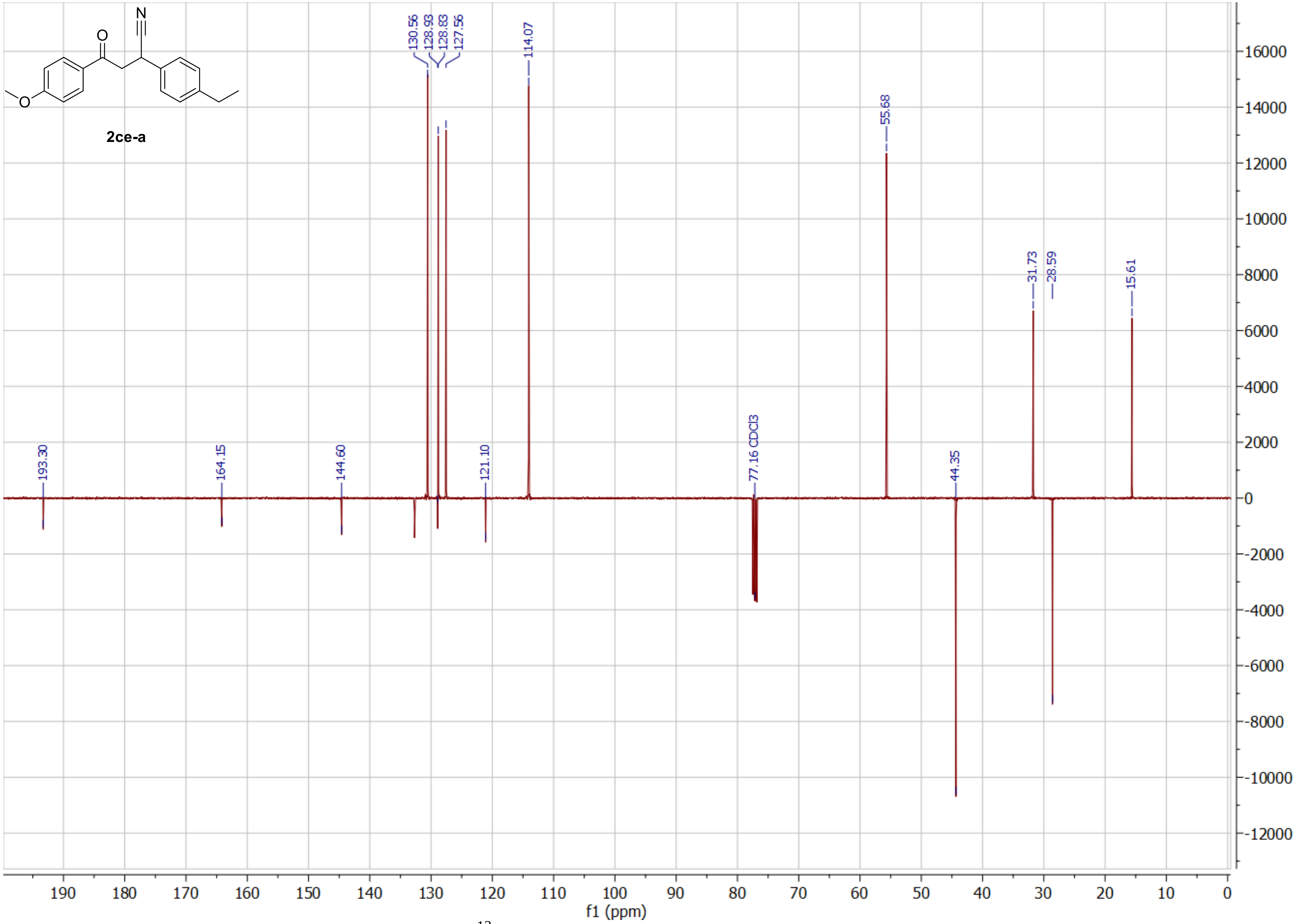

Figure S8. ^{13}C NMR spectrum of **2ce-a** in $CDCl_3$ (101 MHz)

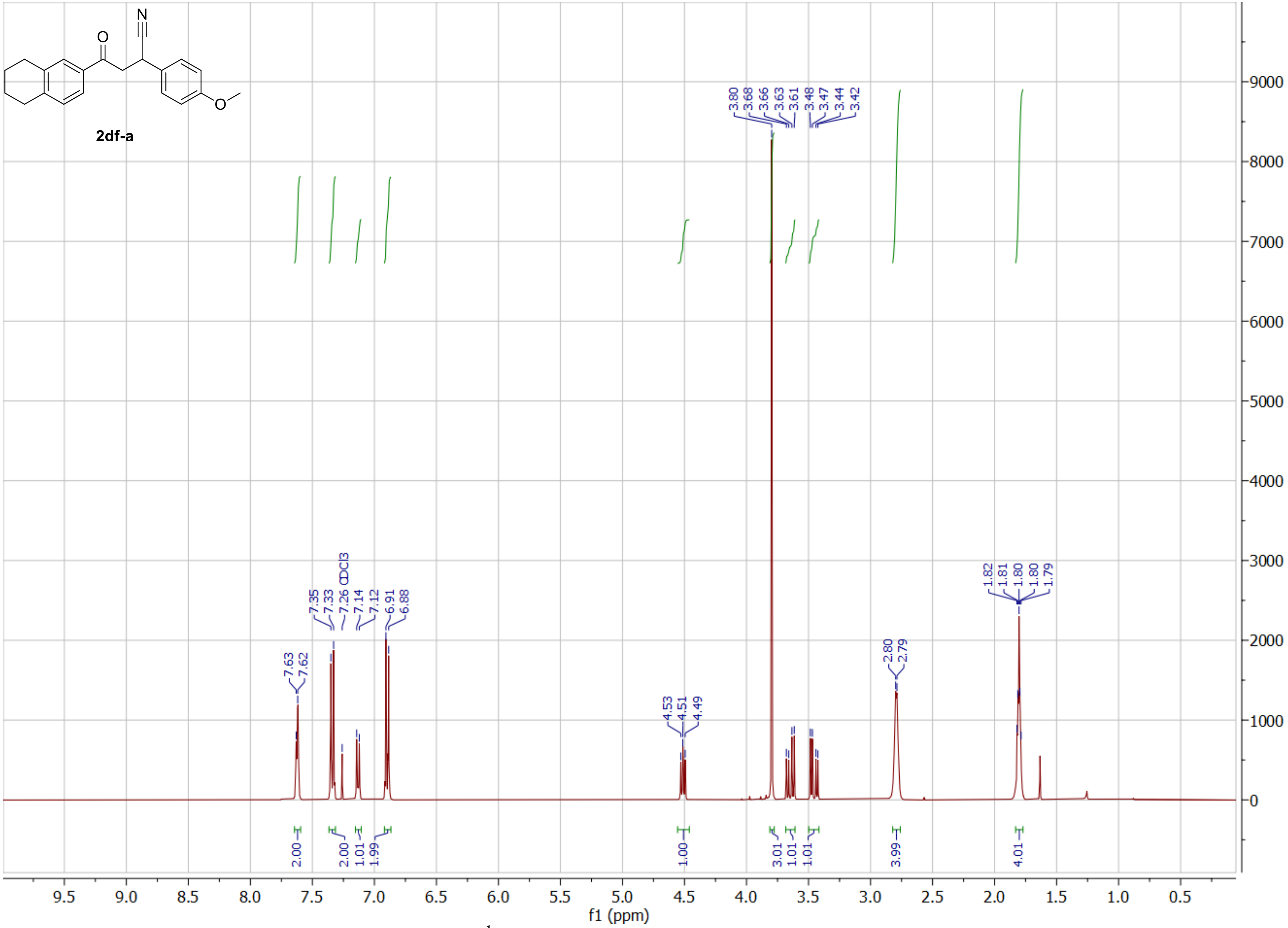

Figure S9. ^{1}H NMR spectrum of **2df-a** in $CDCl_3$ (400 MHz)

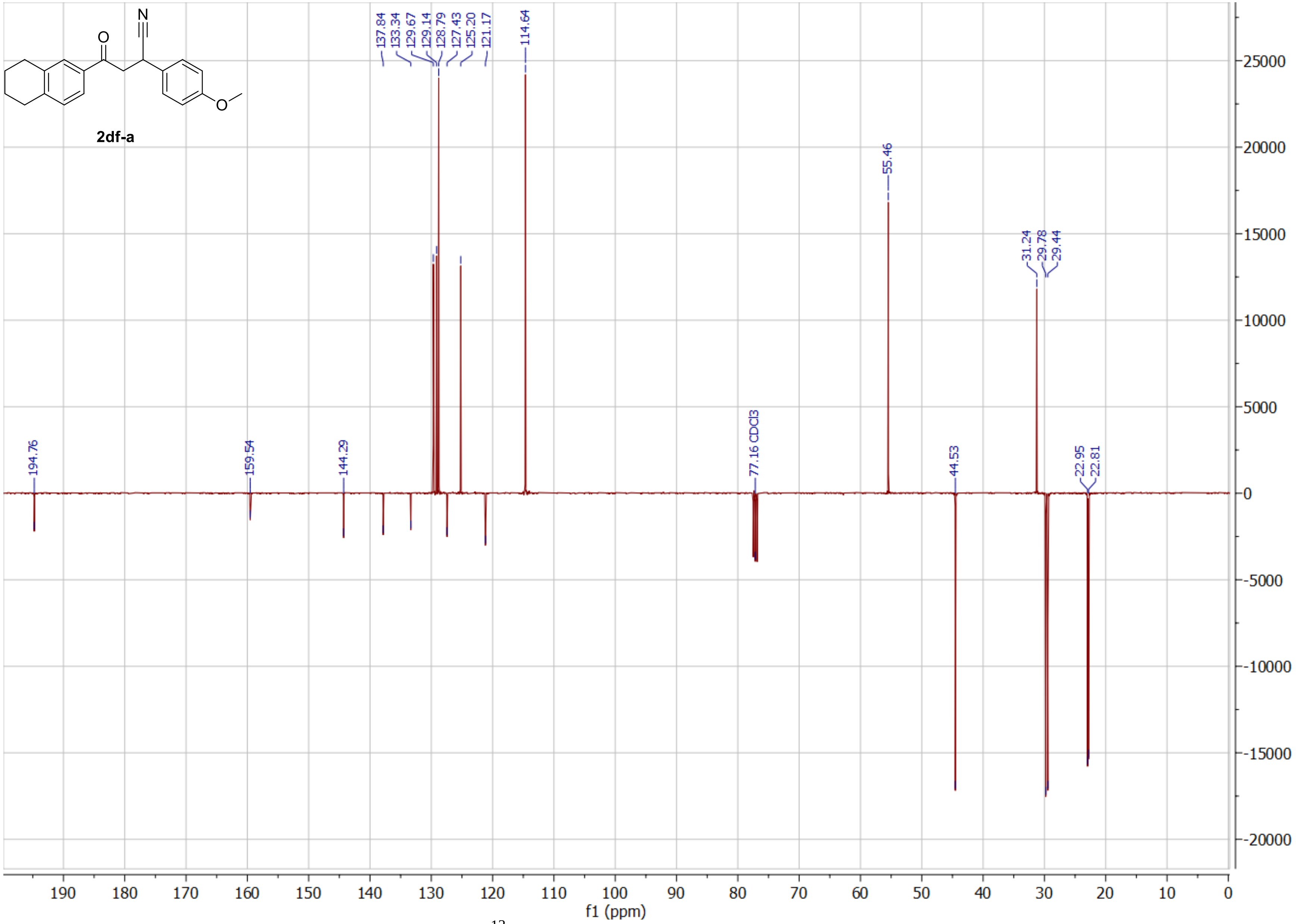

Figure S10. ^{13}C NMR spectrum of **2df-a** in $CDCl_3$ (101 MHz)

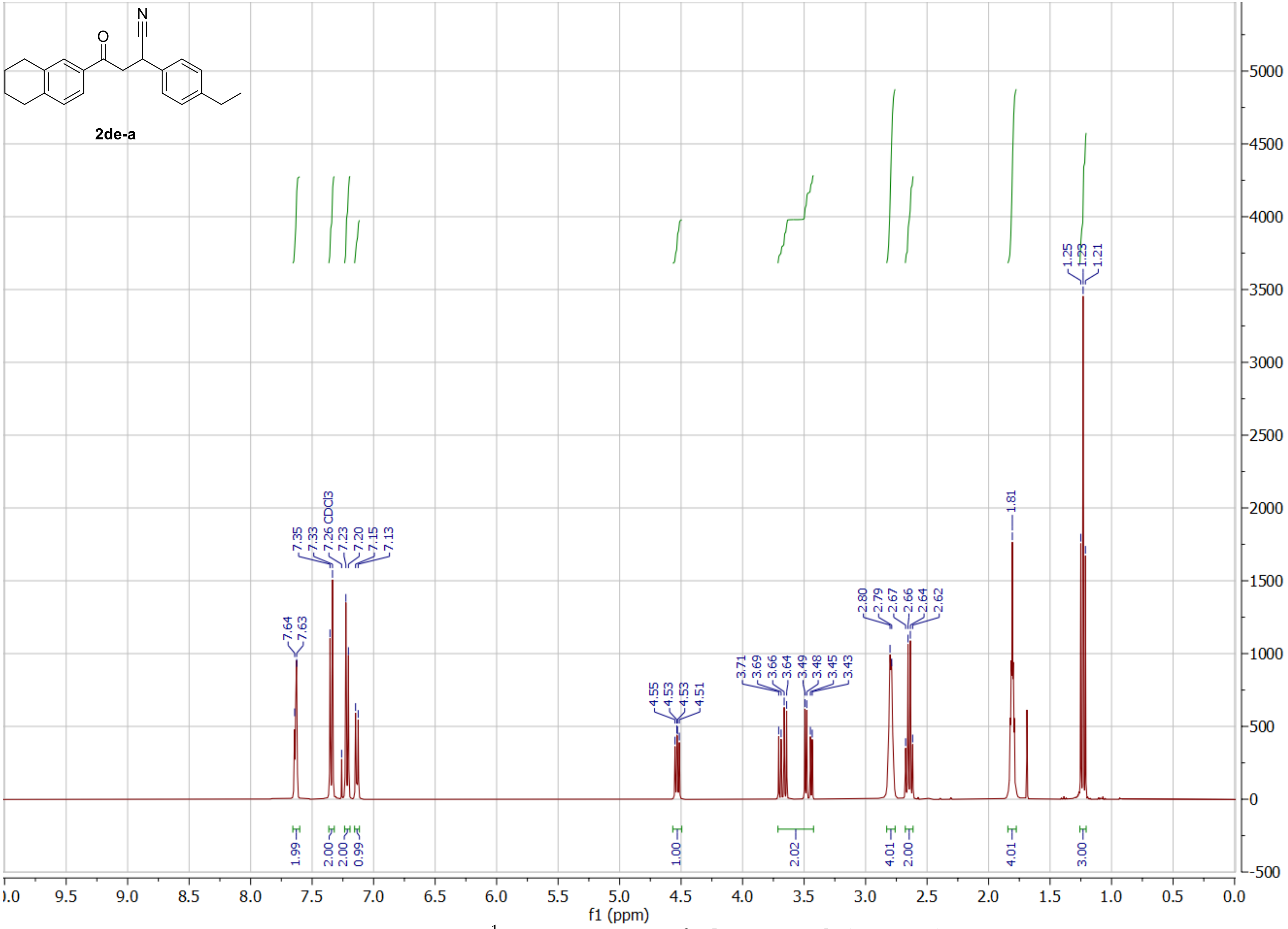

Figure S11. ^{1}H NMR spectrum of **2de-a** in $CDCl_3$ (400 MHz)

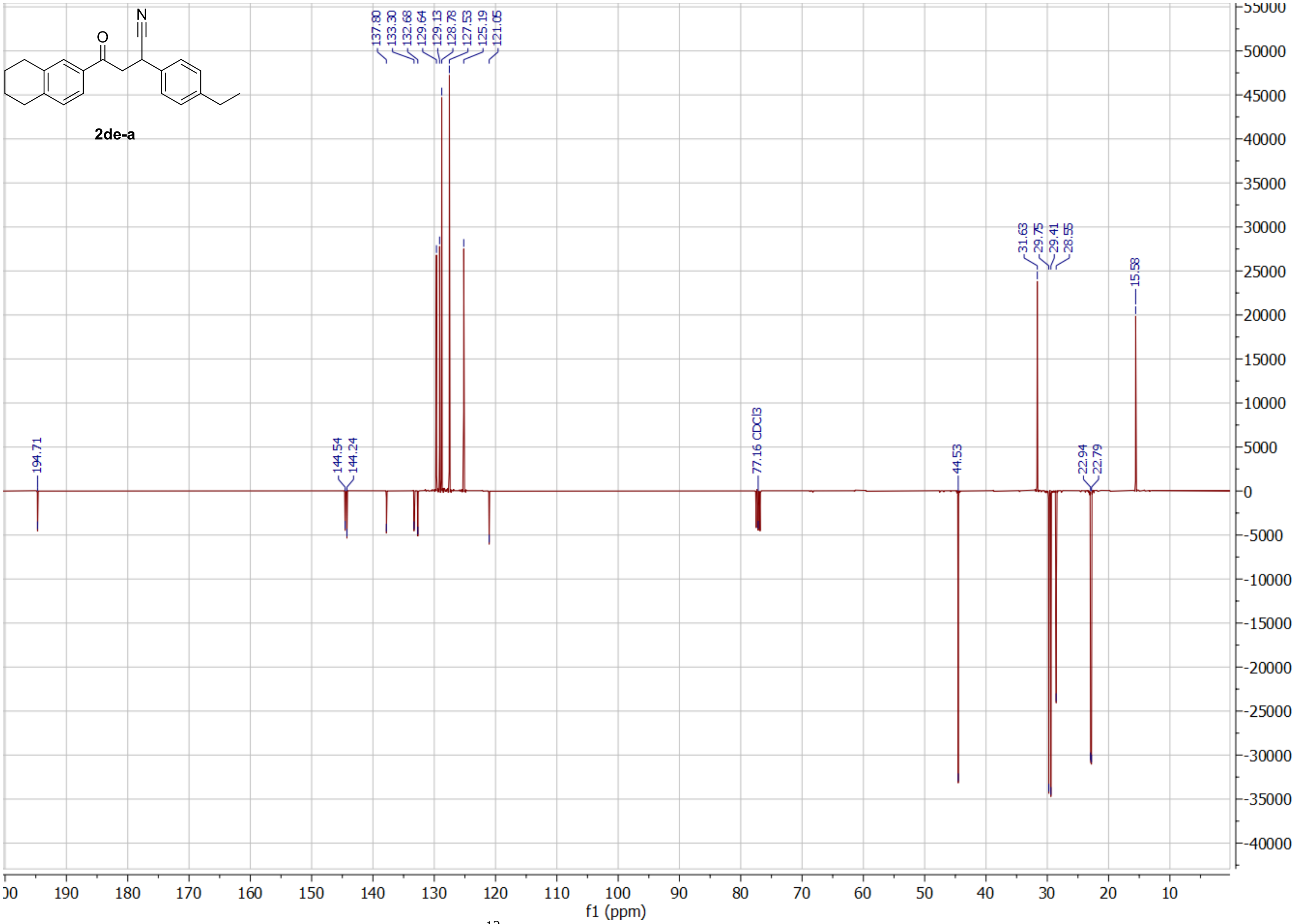

Figure S12. ^{13}C NMR spectrum of **2de-a** in $CDCl_3$ (101 MHz)

^{1}H and ^{13}C NMR spectral charts for 4-benzyl-5-hydroxy-3,5-diaryl-1*H*-pyrrol-2(5*H*)-ones 4

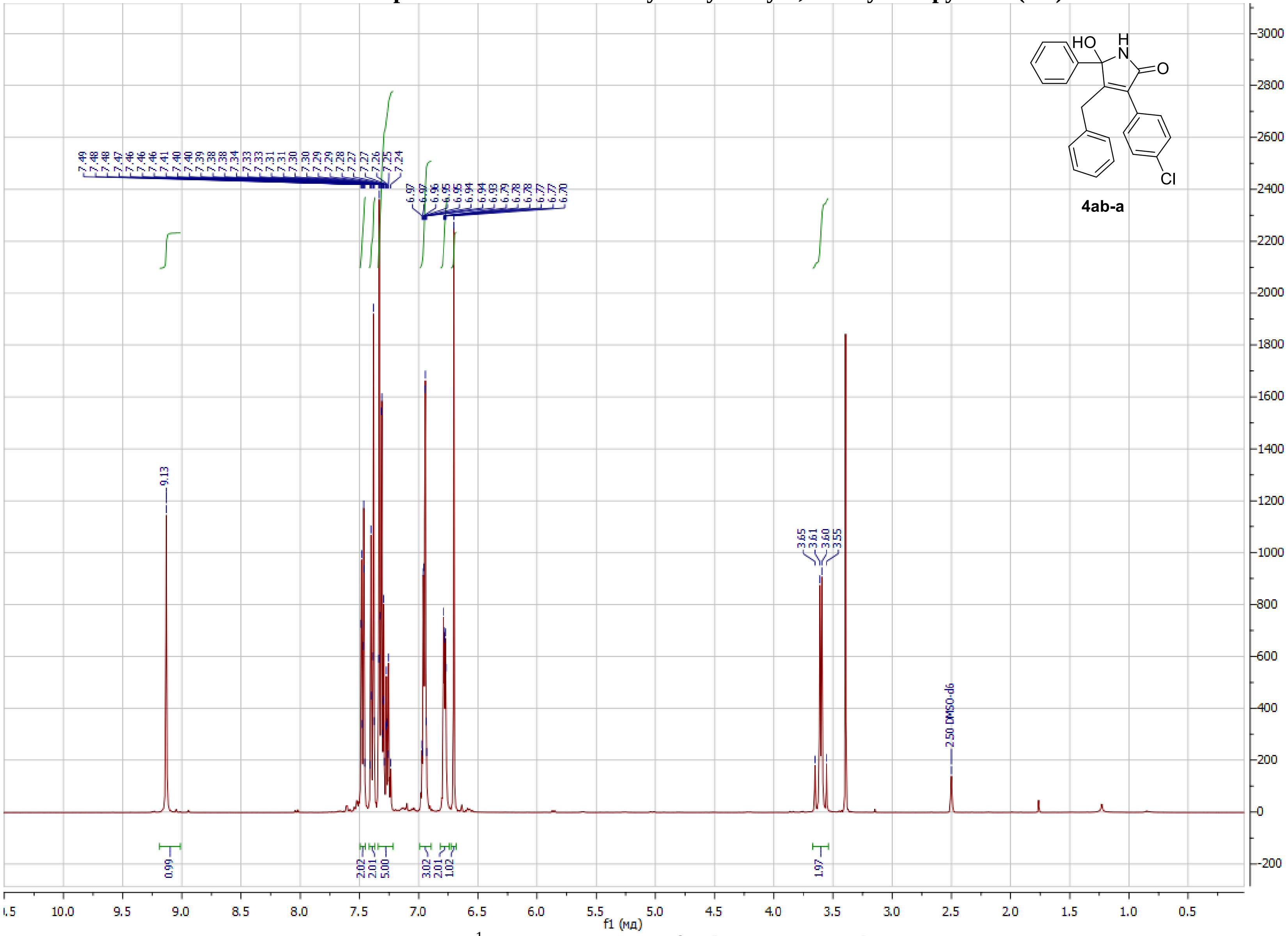

Figure S13. ^{1}H NMR spectrum of **4ab-a** in DMSO-d_6 (400 MHz)

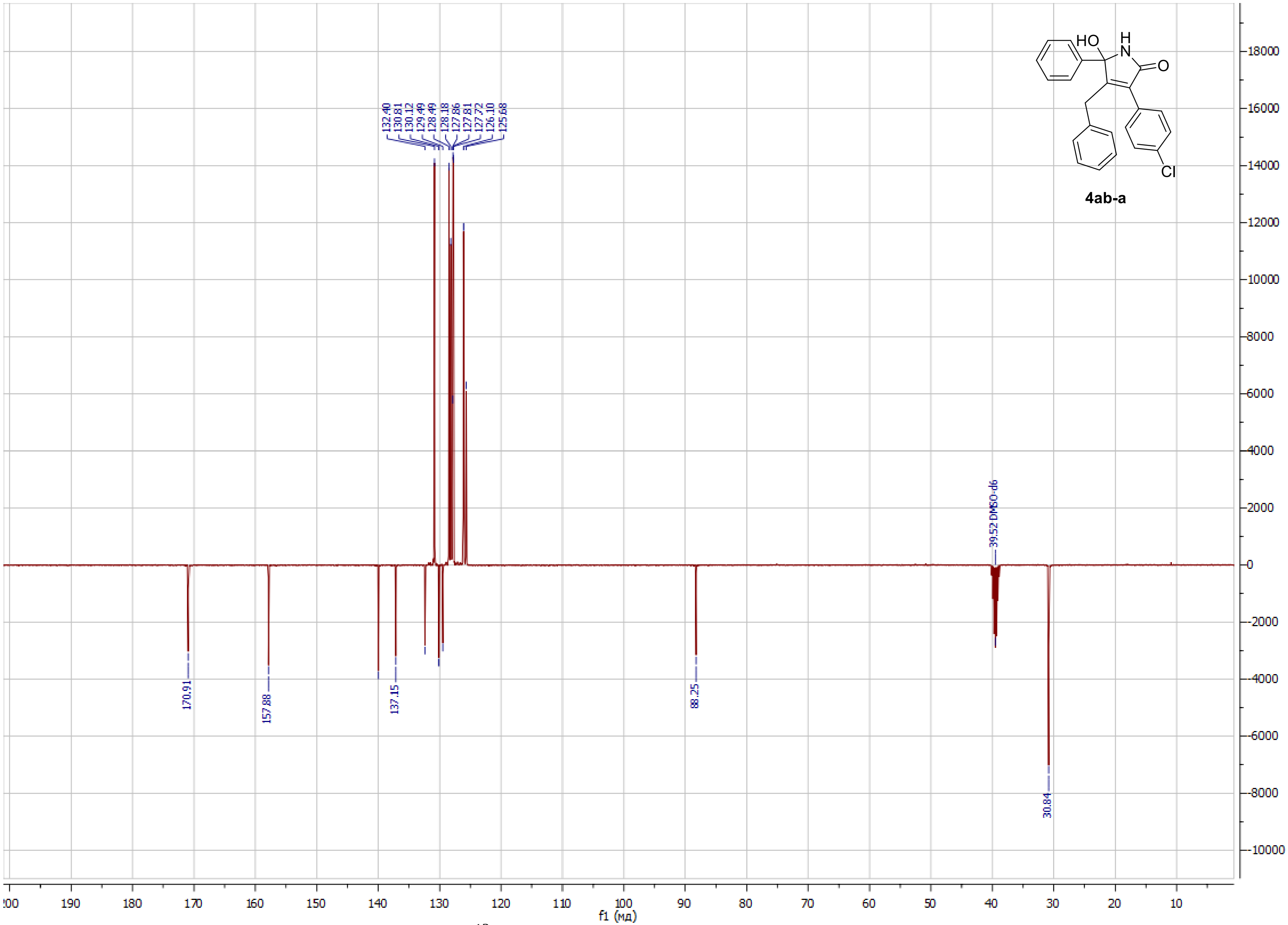

Figure S14. ^{13}C NMR spectrum of **4ab-a** in DMSO-d_6 (101 MHz)

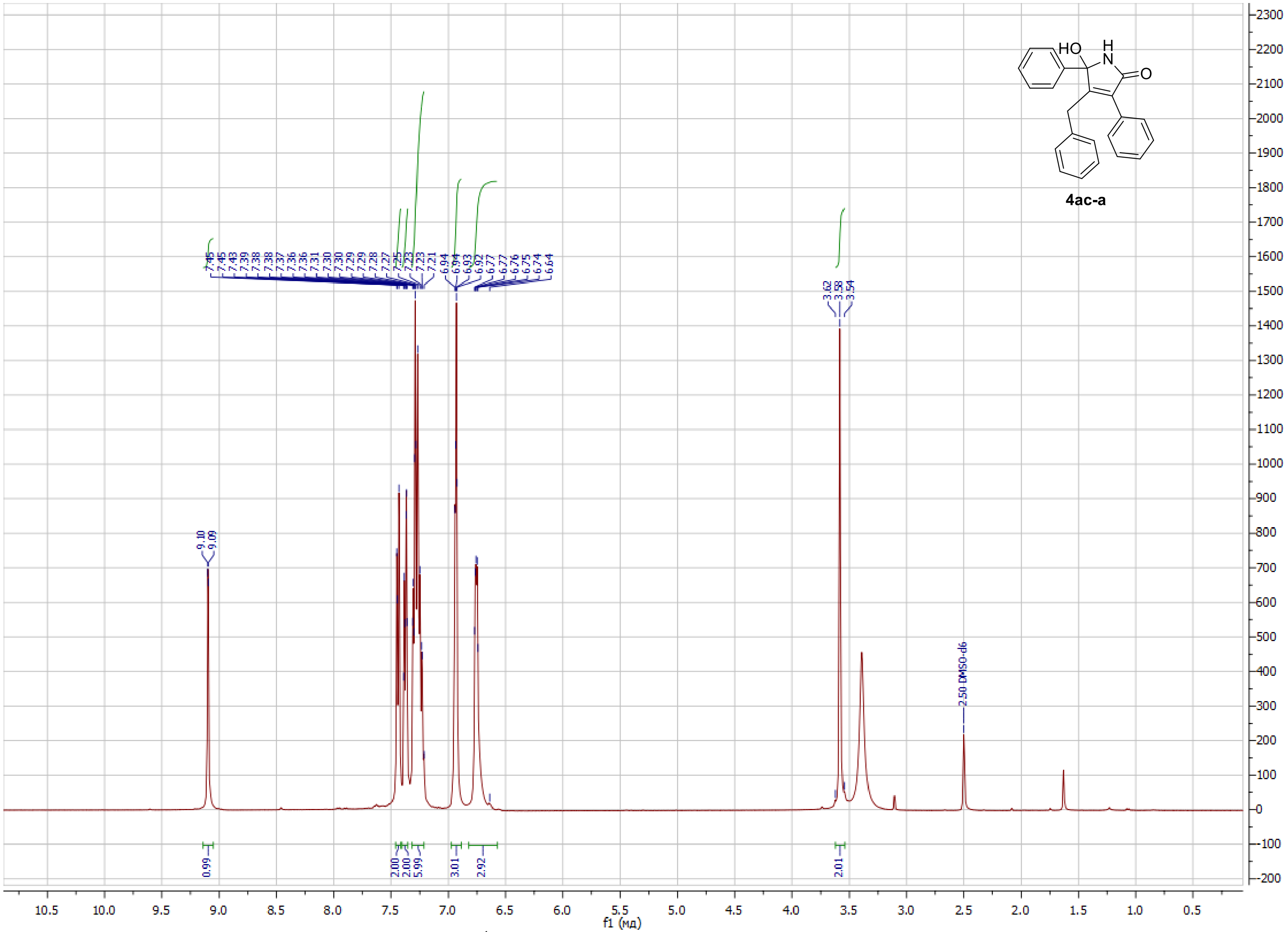

Figure S15. ^{1}H NMR spectrum of **4ac-a** in DMSO-d_6 (400 MHz)

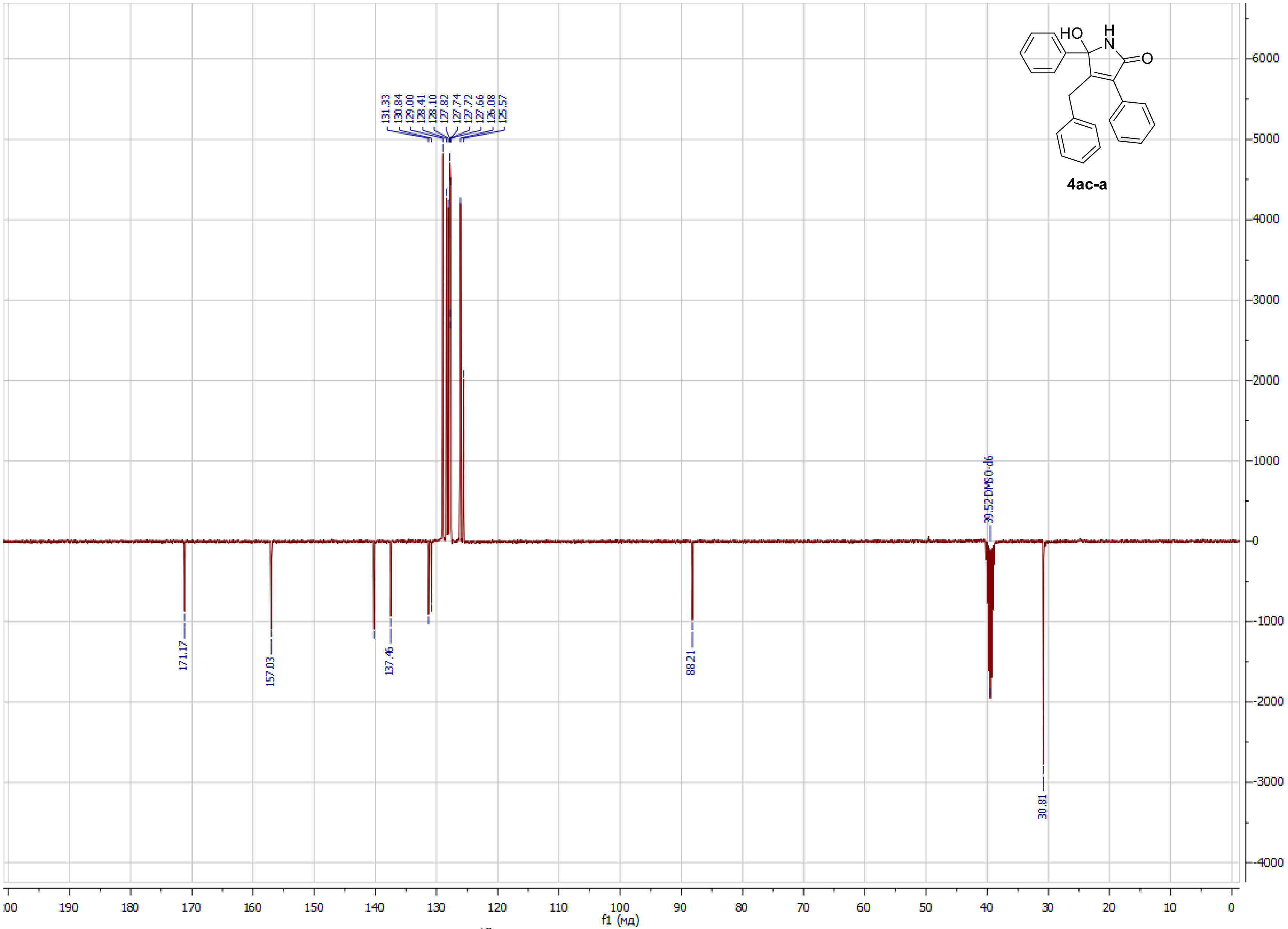

Figure S16. ^{13}C NMR spectrum of **4ac-a** in DMSO-d_6 (101 MHz)

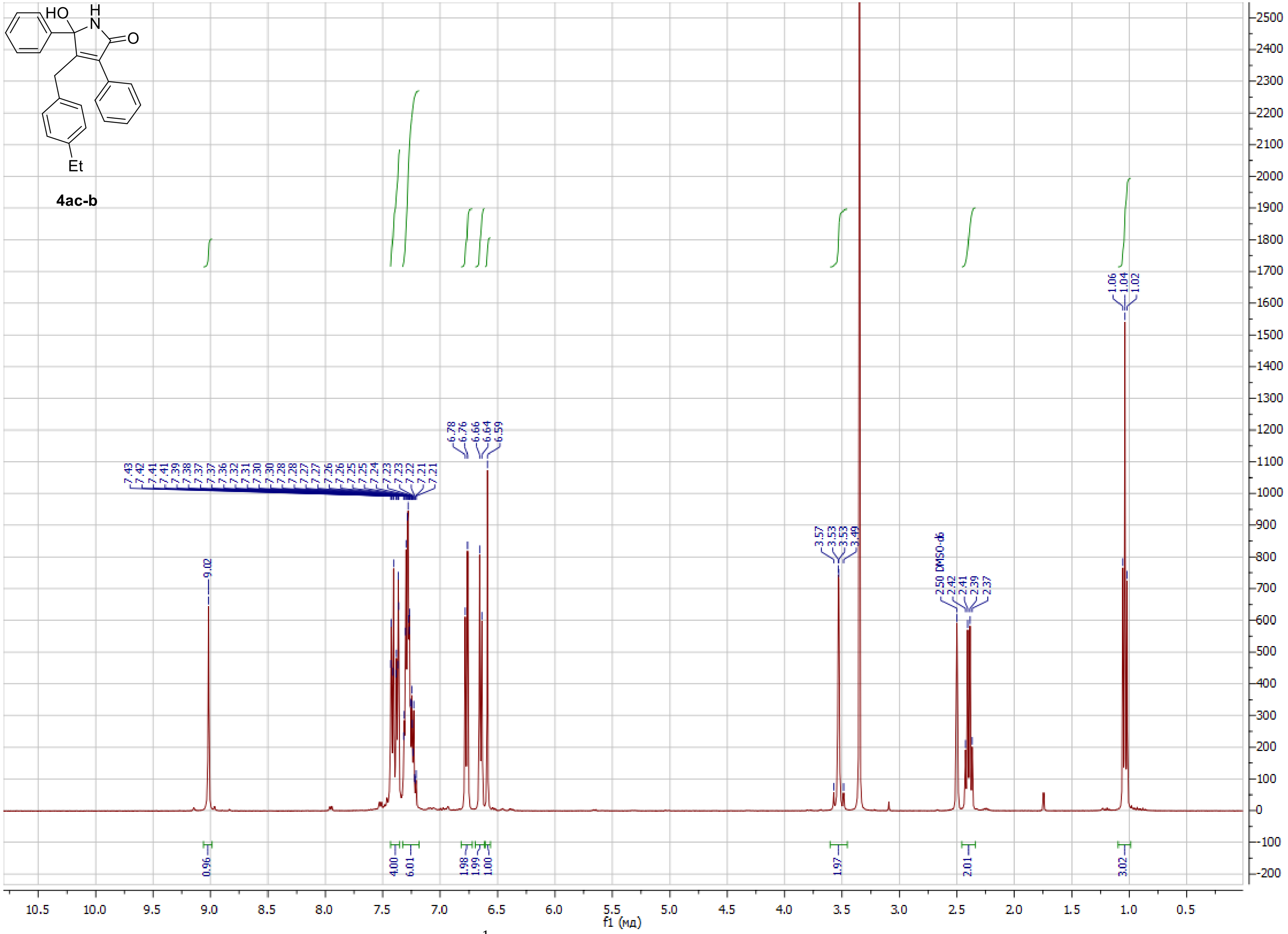

Figure S17. ^{1}H NMR spectrum of **4ac-b** in DMSO-d_6 (400 MHz)

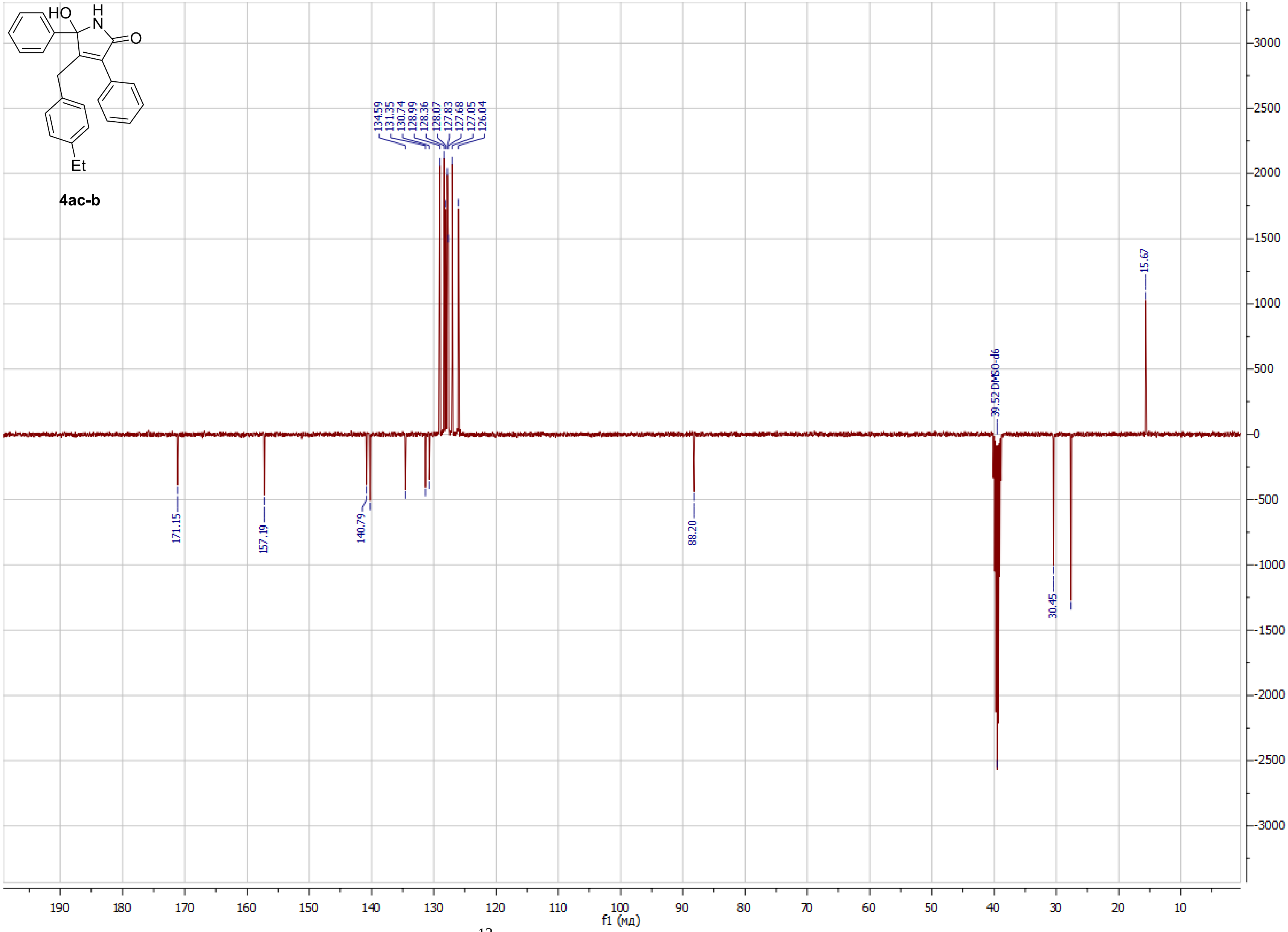

Figure S18. ^{13}C NMR spectrum of **4ac-b** in DMSO-d_6 (101 MHz)

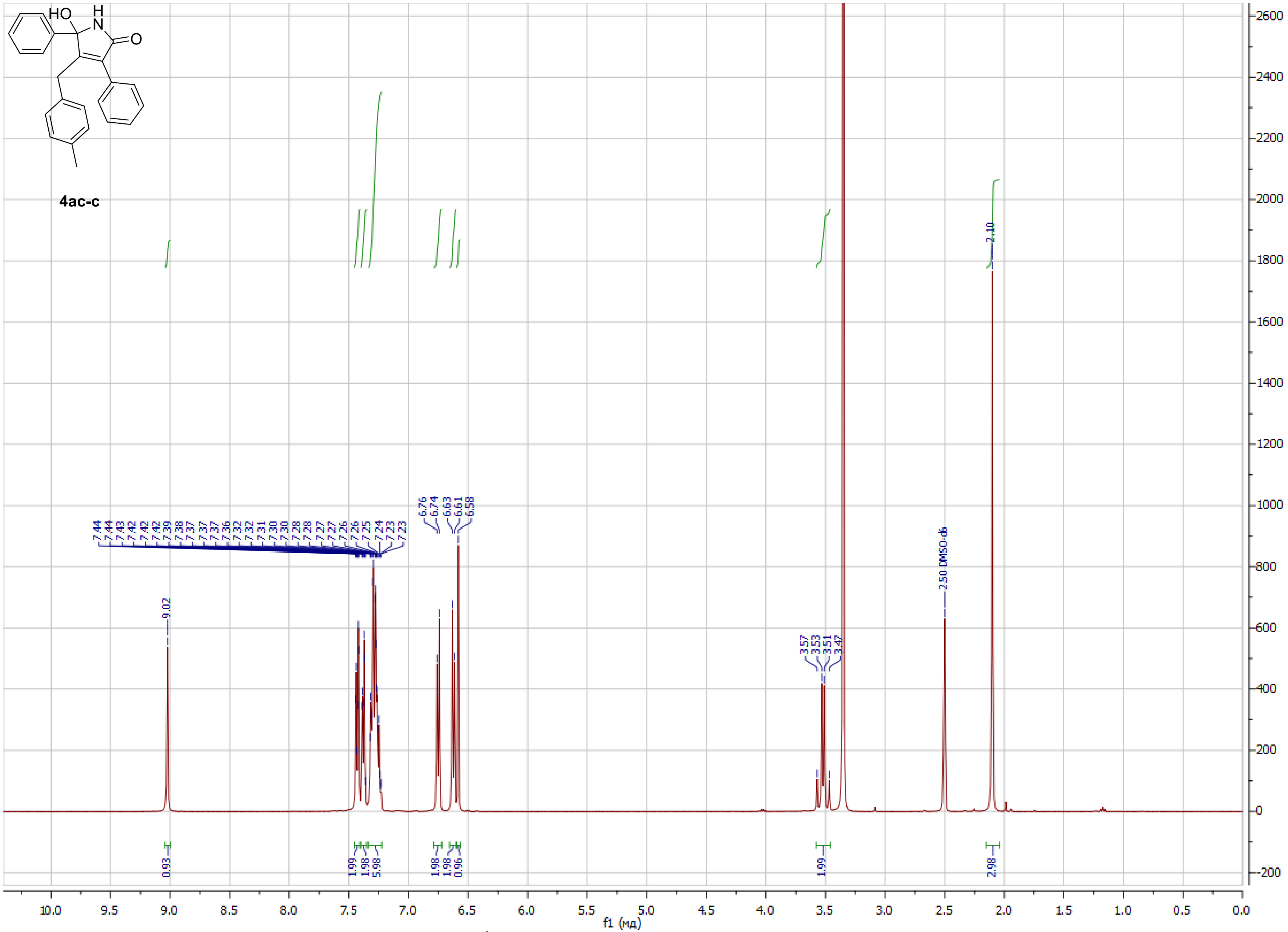

Figure S19. ^{1}H NMR spectrum of **4ac-c** in DMSO-d_6 (400 MHz)

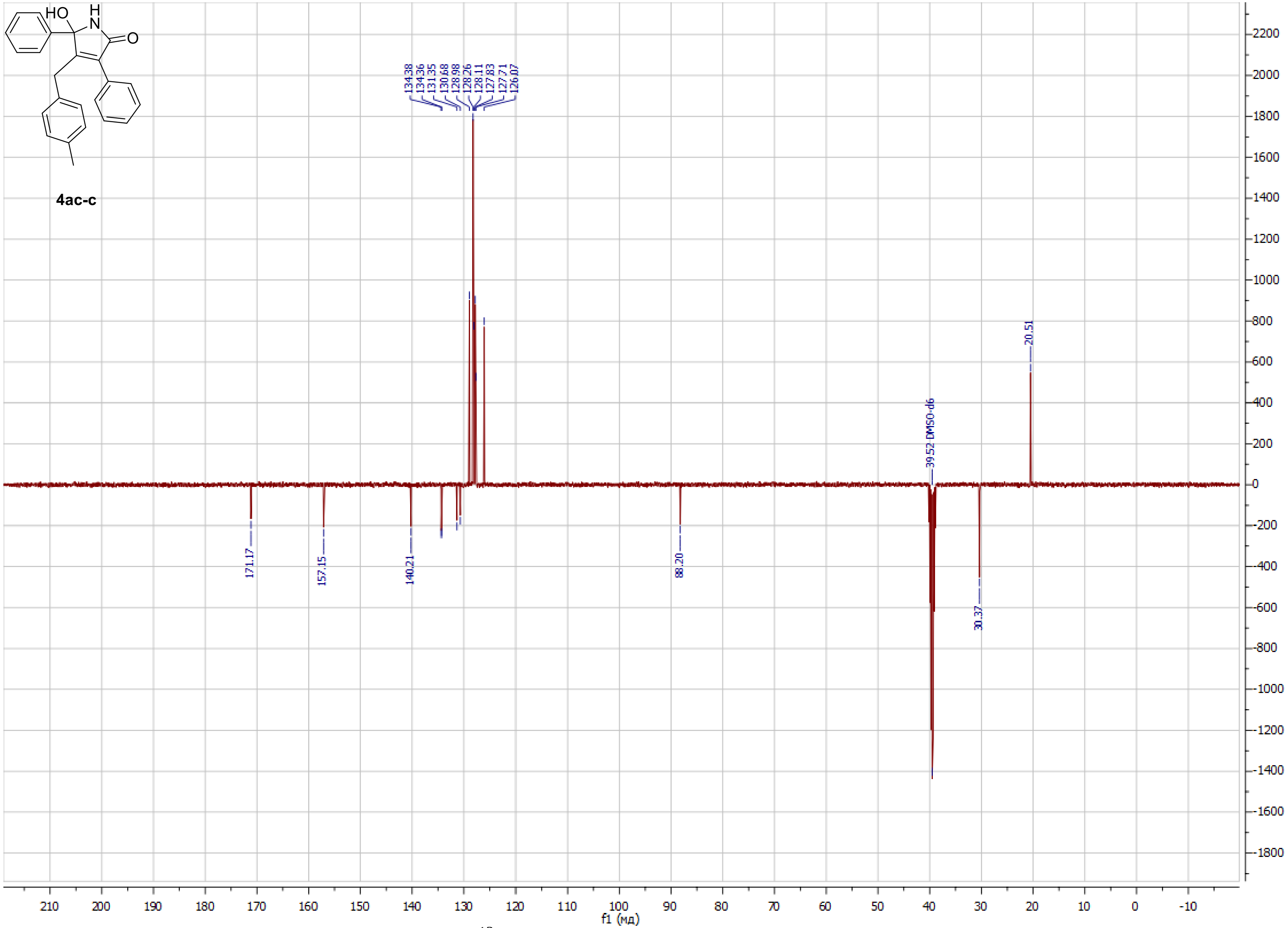

Figure S20. ^{13}C NMR spectrum of **4ac-c** in DMSO-d_6 (101 MHz)

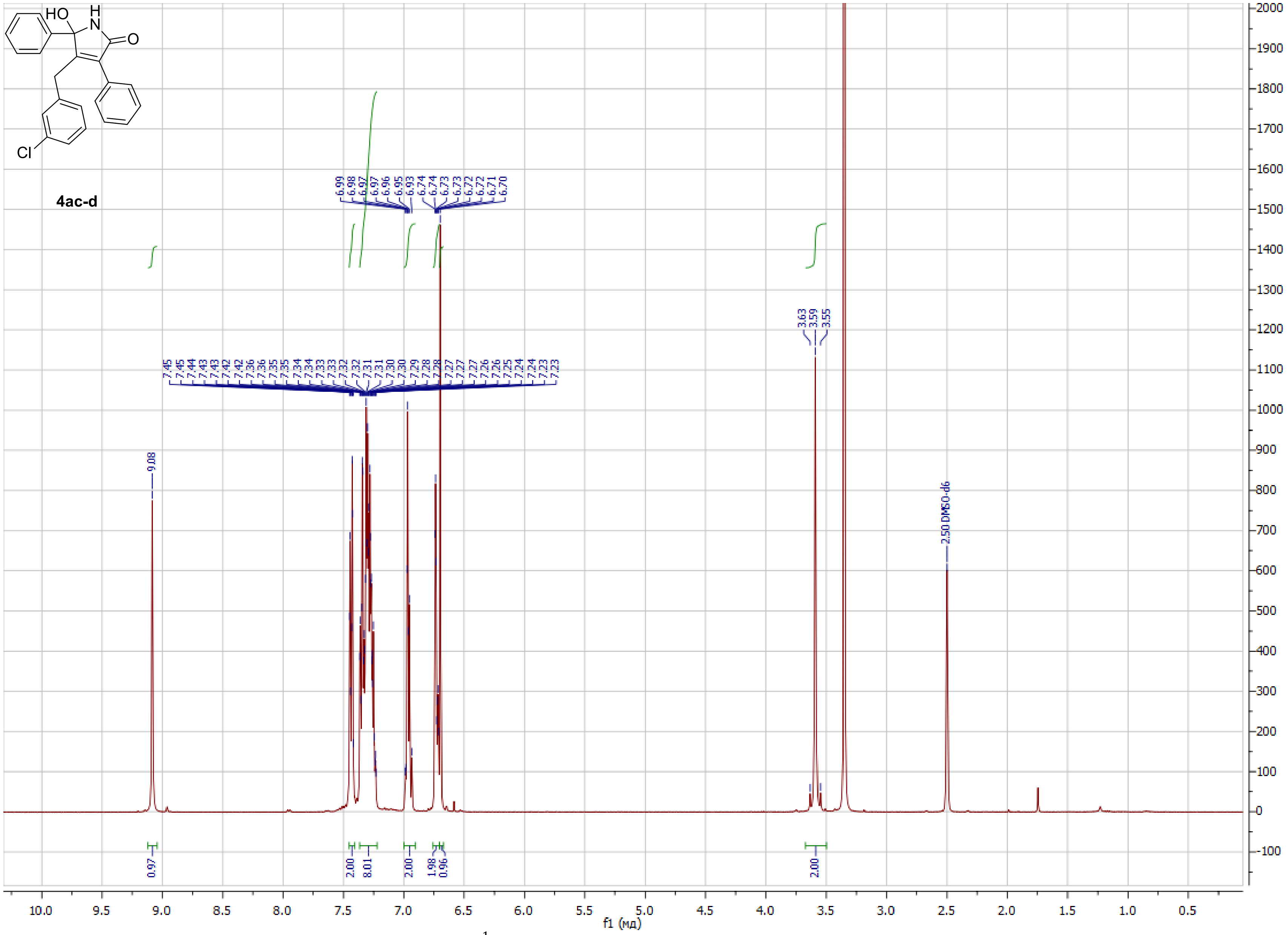

Figure S21. ^{1}H NMR spectrum of **4ac-d** in DMSO-d_6 (400 MHz)

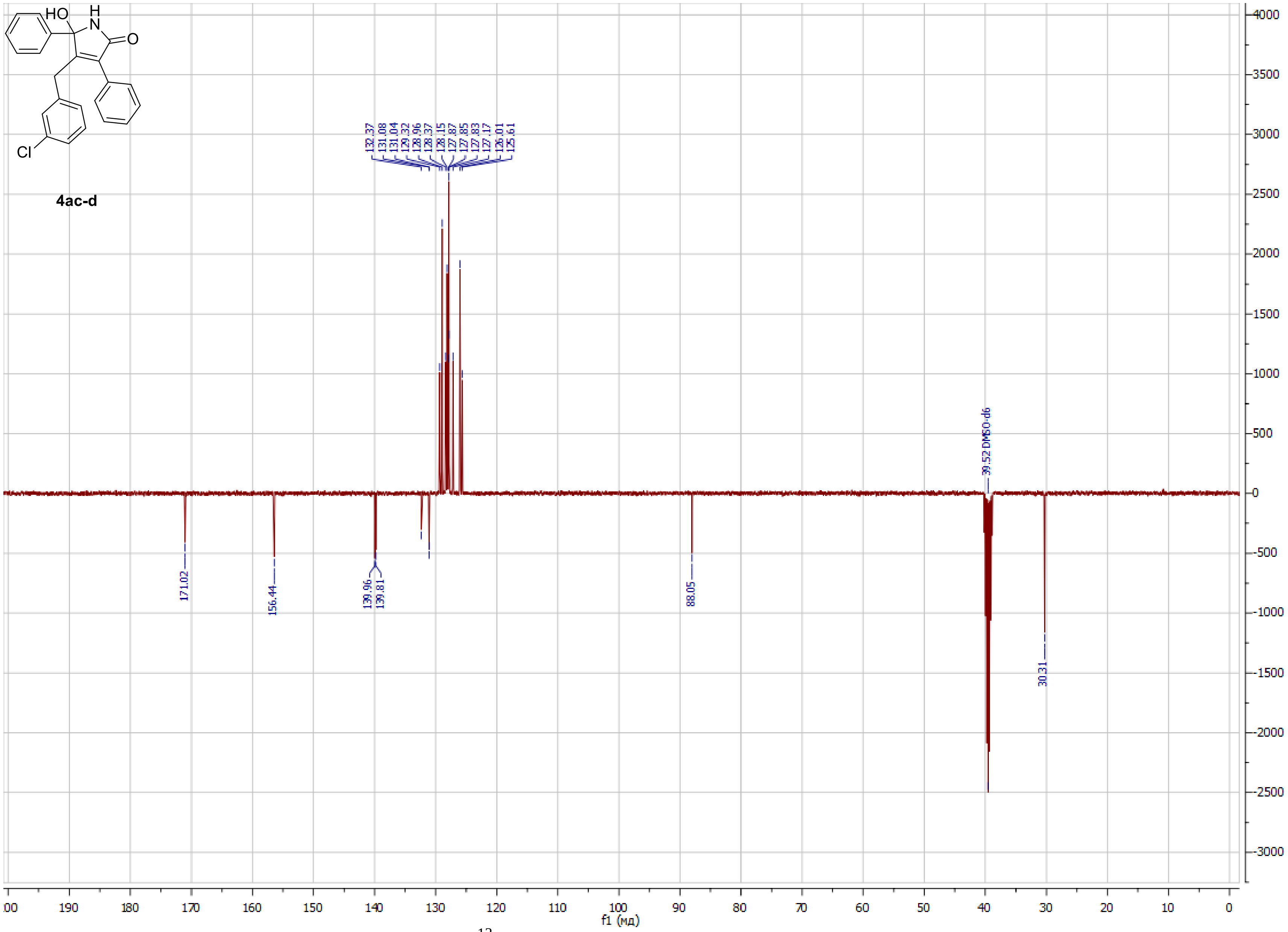

Figure S22. ^{13}C NMR spectrum of **4ac-d** in DMSO-d_6 (101 MHz)

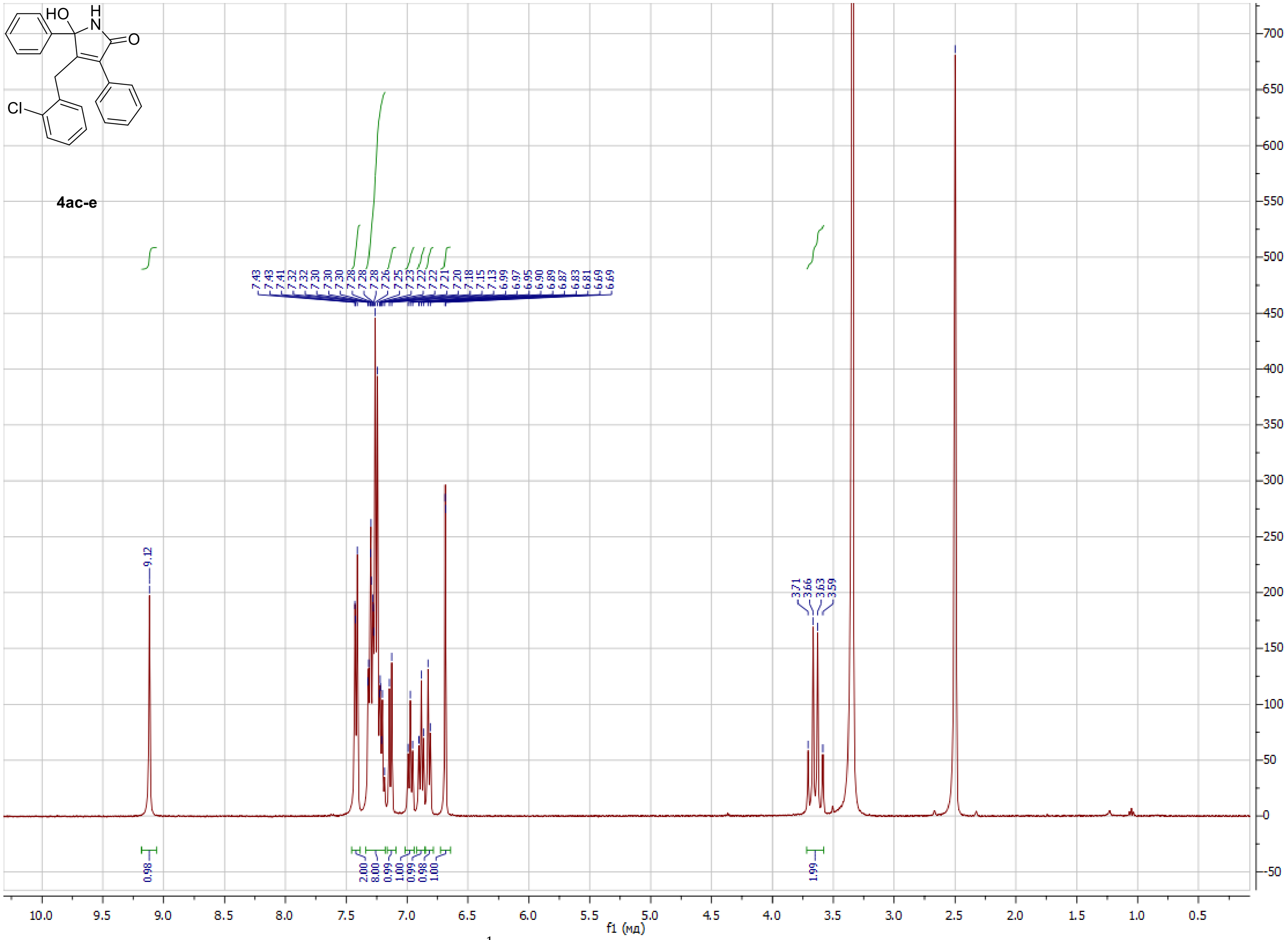

Figure S23. ^{1}H NMR spectrum of **4ac-e** in DMSO-d_6 (400 MHz)

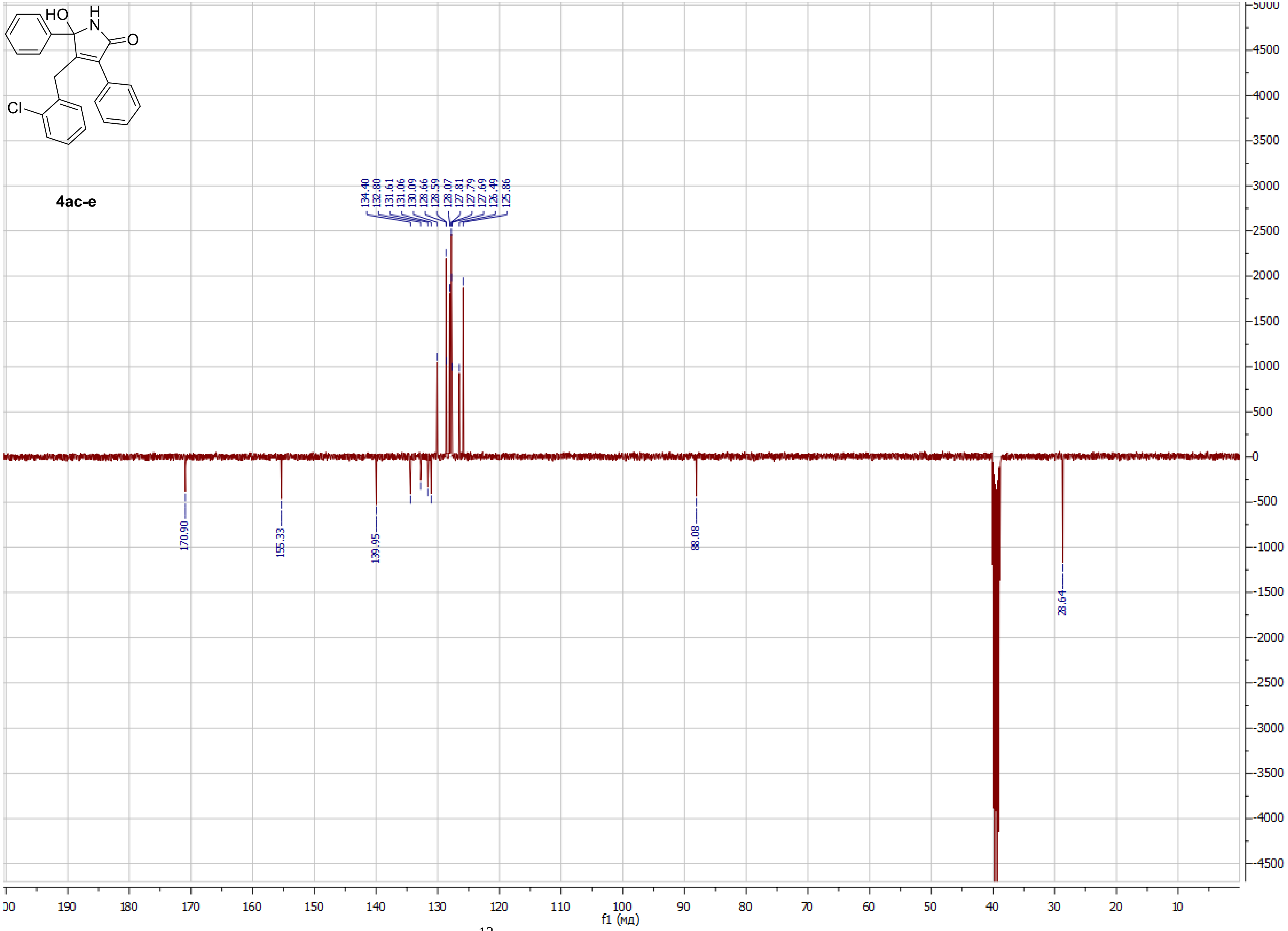

Figure S24. ^{13}C NMR spectrum of **4ac-e** in DMSO-d_6 (101 MHz)

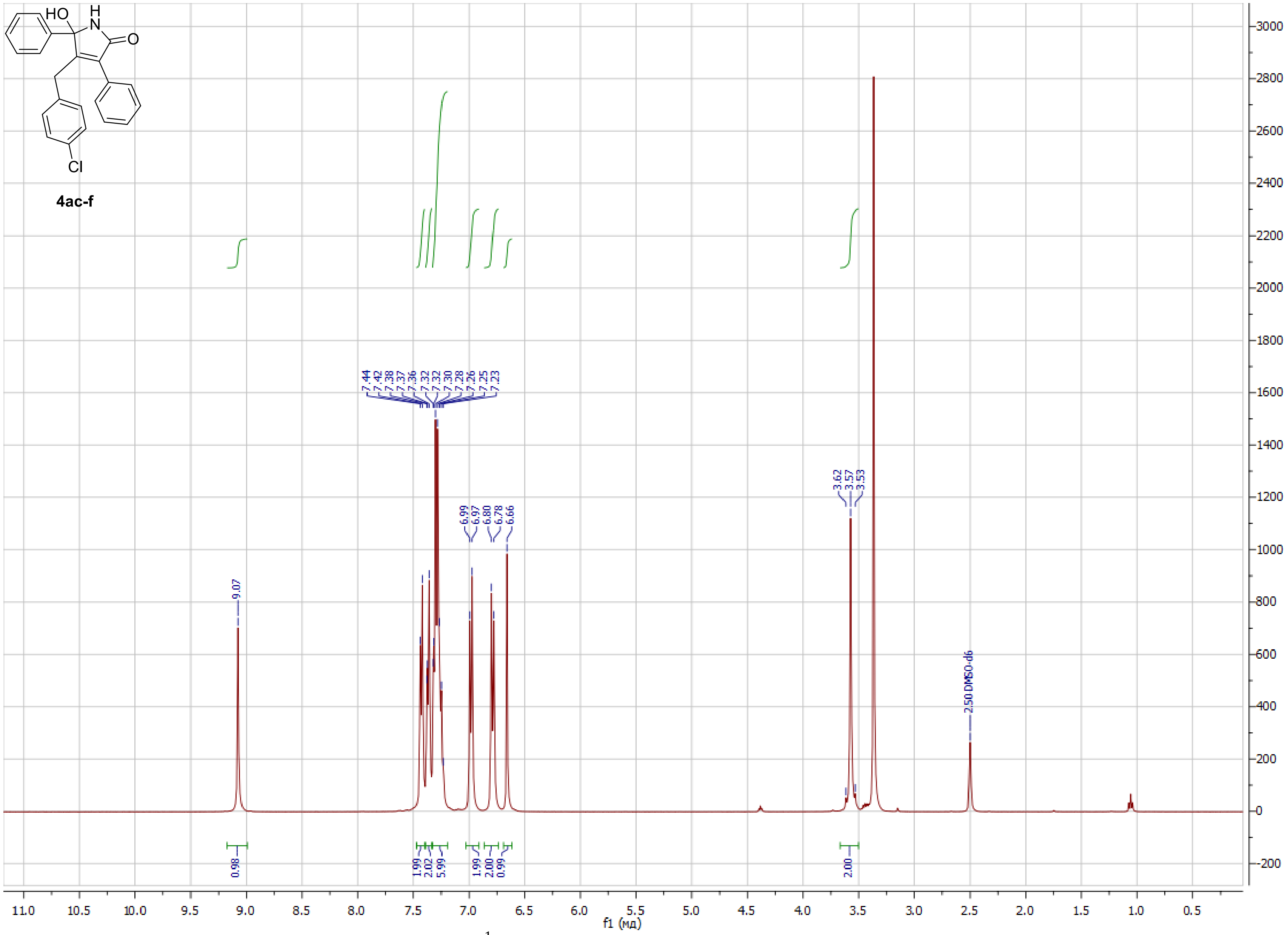

Figure S25. ^{1}H NMR spectrum of **4ac-f** in DMSO-d_6 (400 MHz)

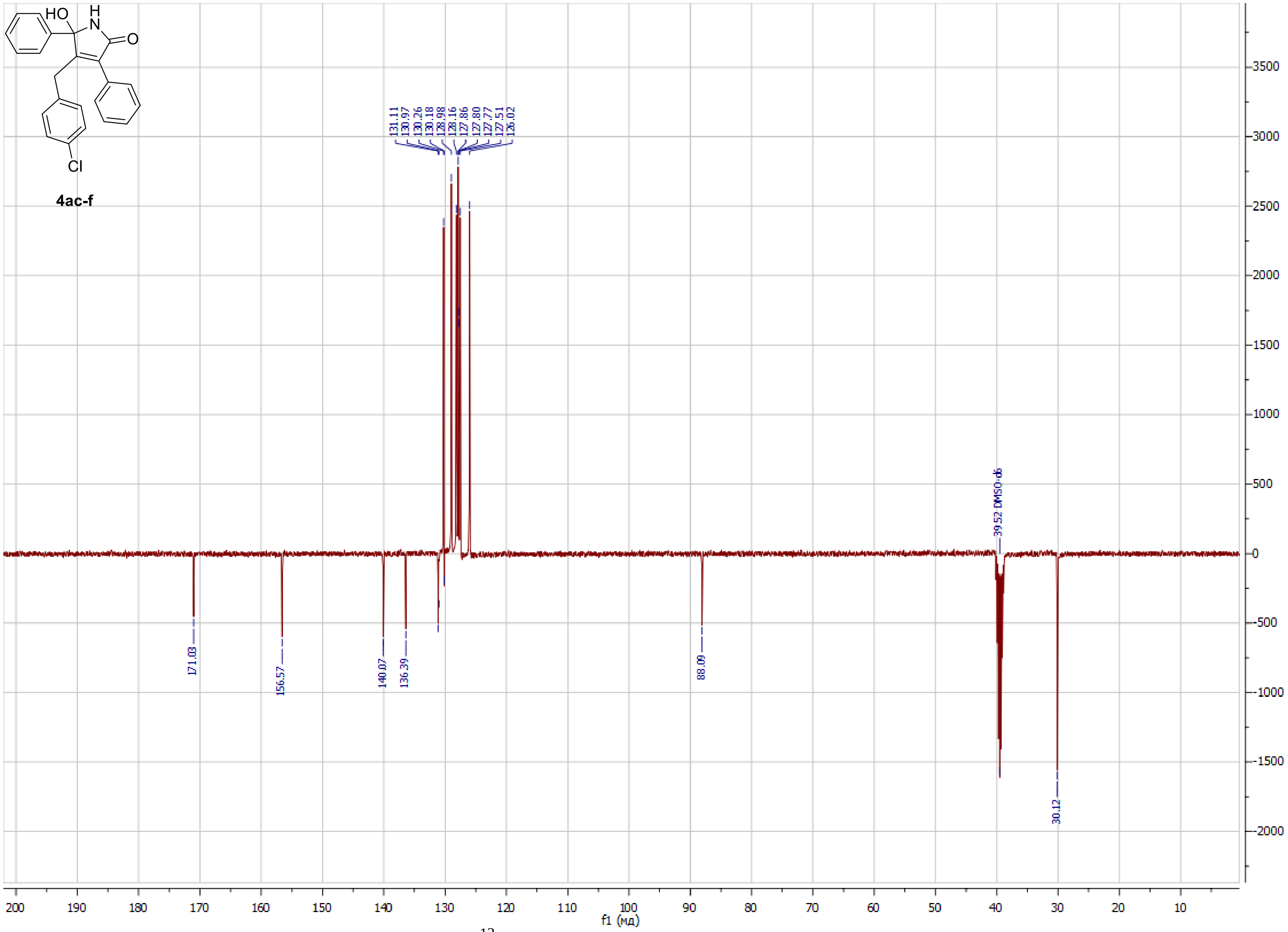

Figure S26. ^{13}C NMR spectrum of **4ac-f** in DMSO-d_6 (101 MHz)

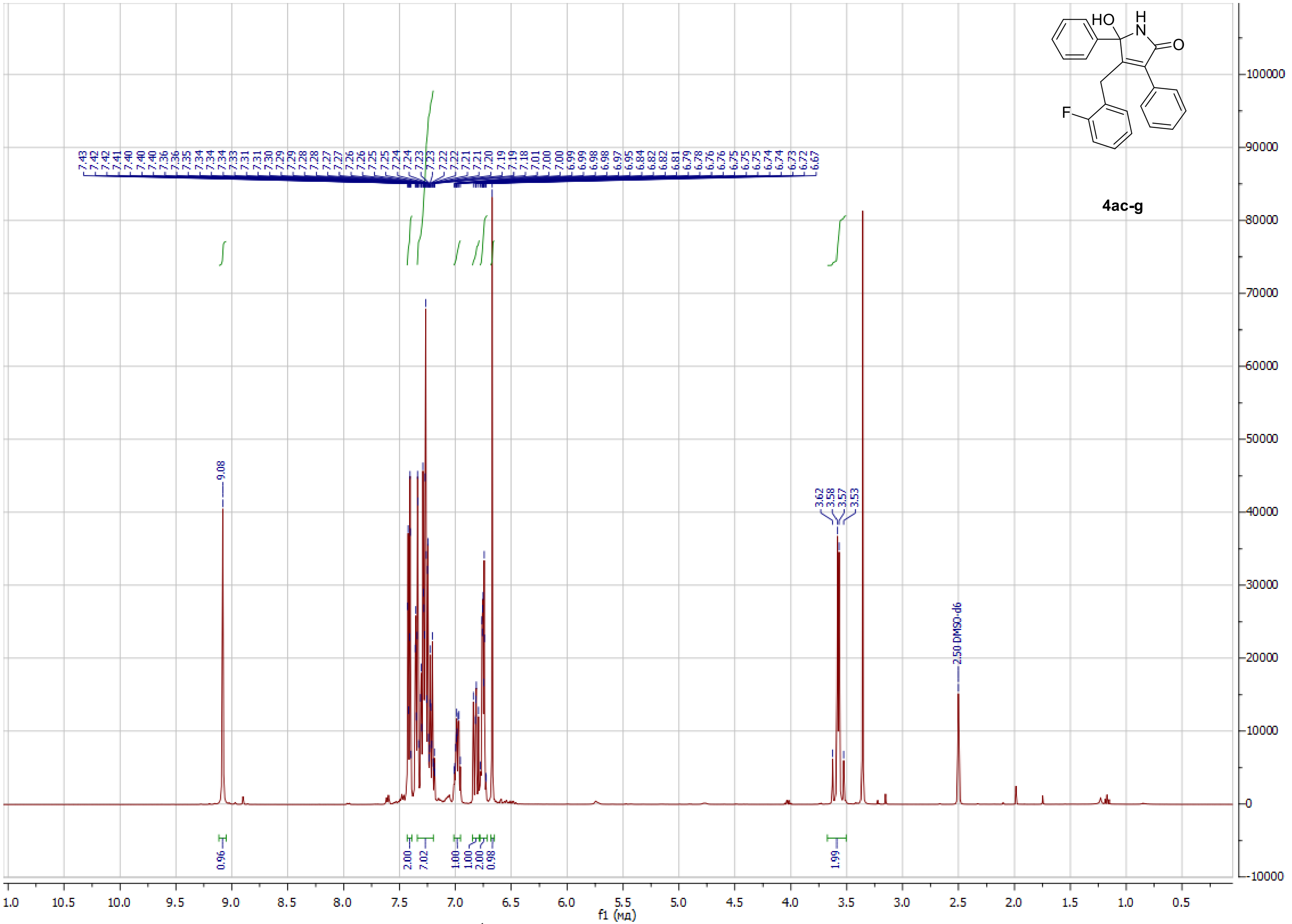

Figure S27. ^{1}H NMR spectrum of **4ac-g** in DMSO-d_6 (400 MHz)

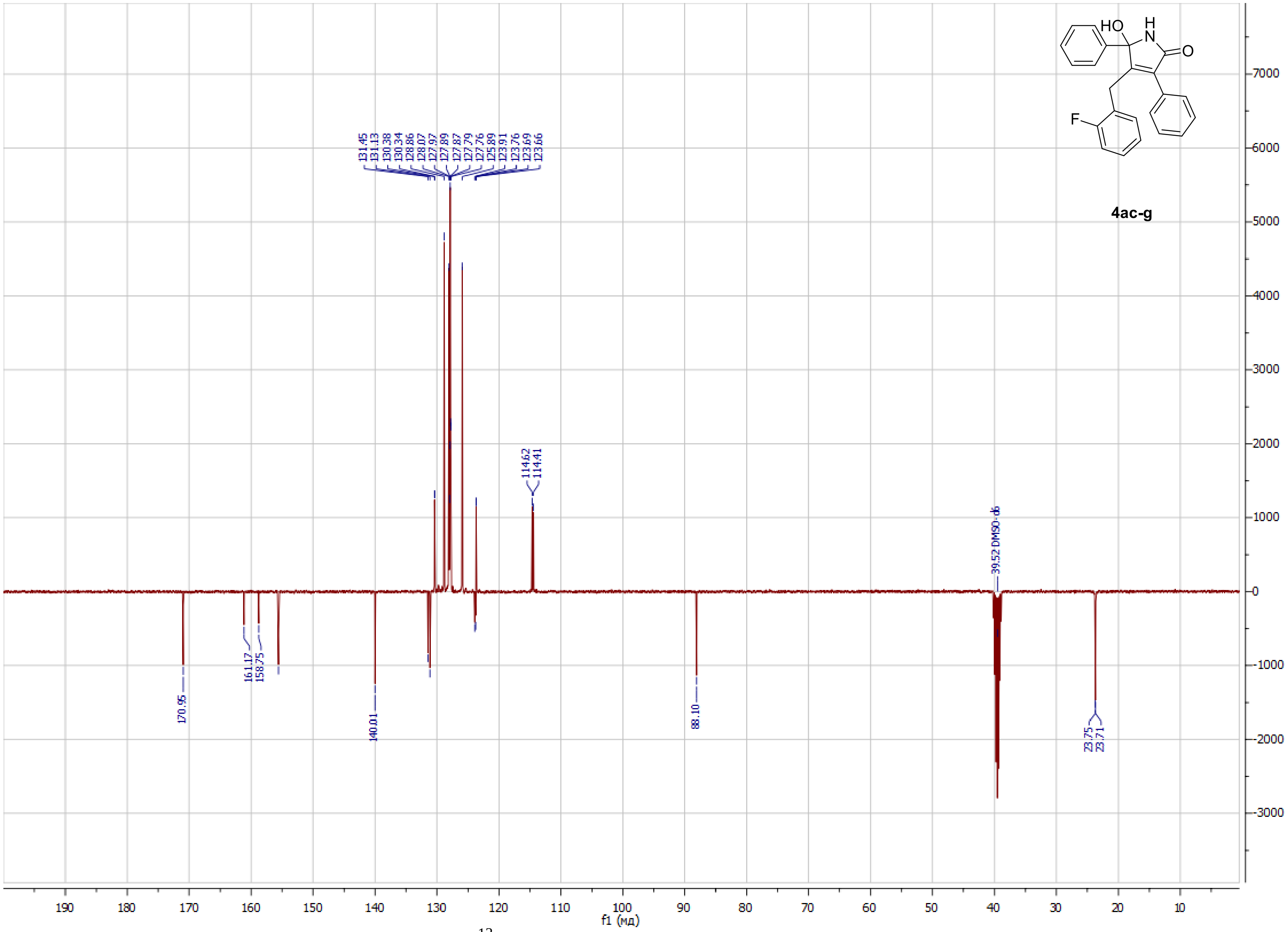

Figure S28. ^{13}C NMR spectrum of **4ac-g** in DMSO-d_6 (101 MHz)

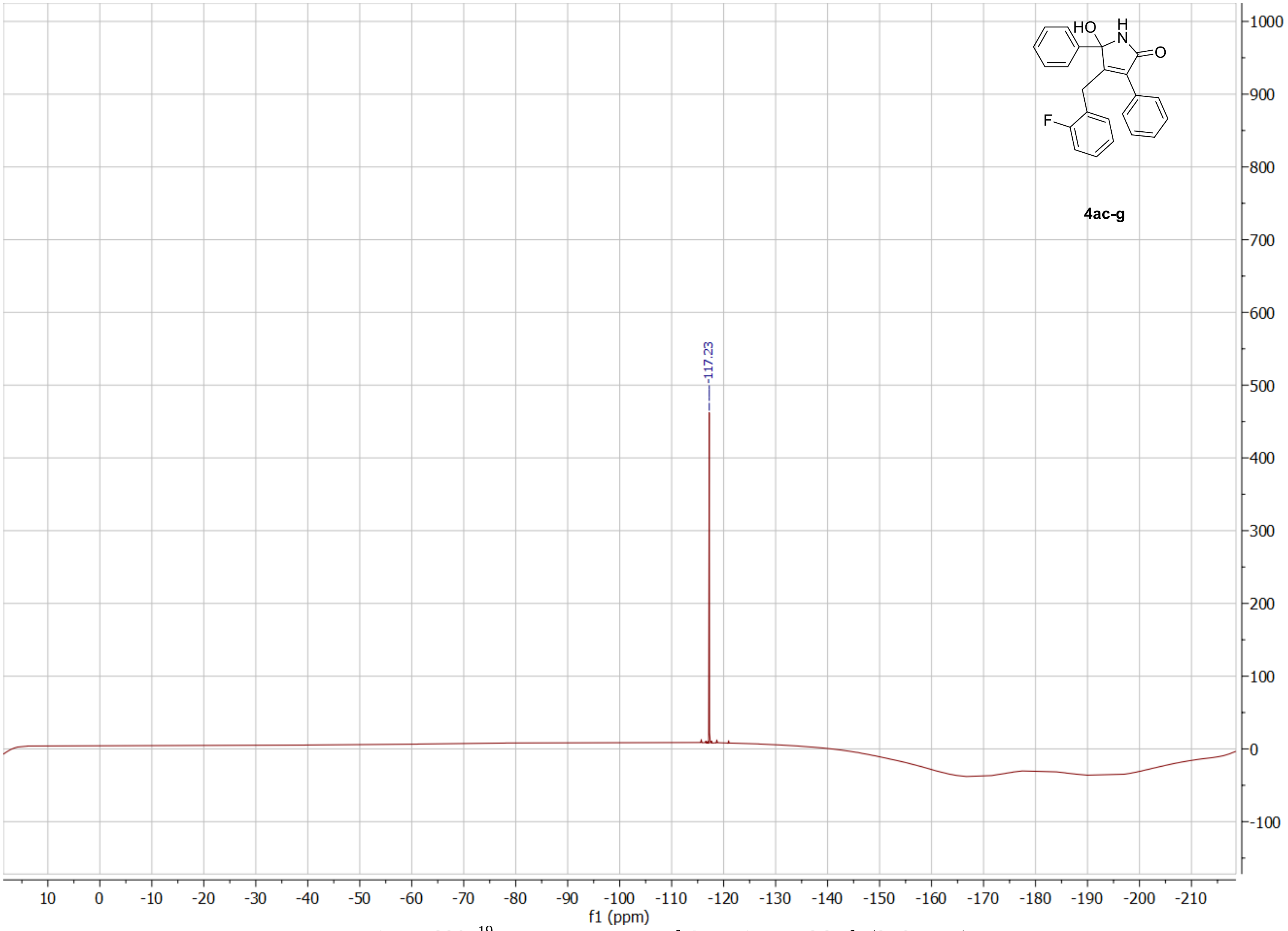

Figure S29. ^{19}F NMR spectrum of **4ac-g** in DMSO-d_6 (376 MHz)

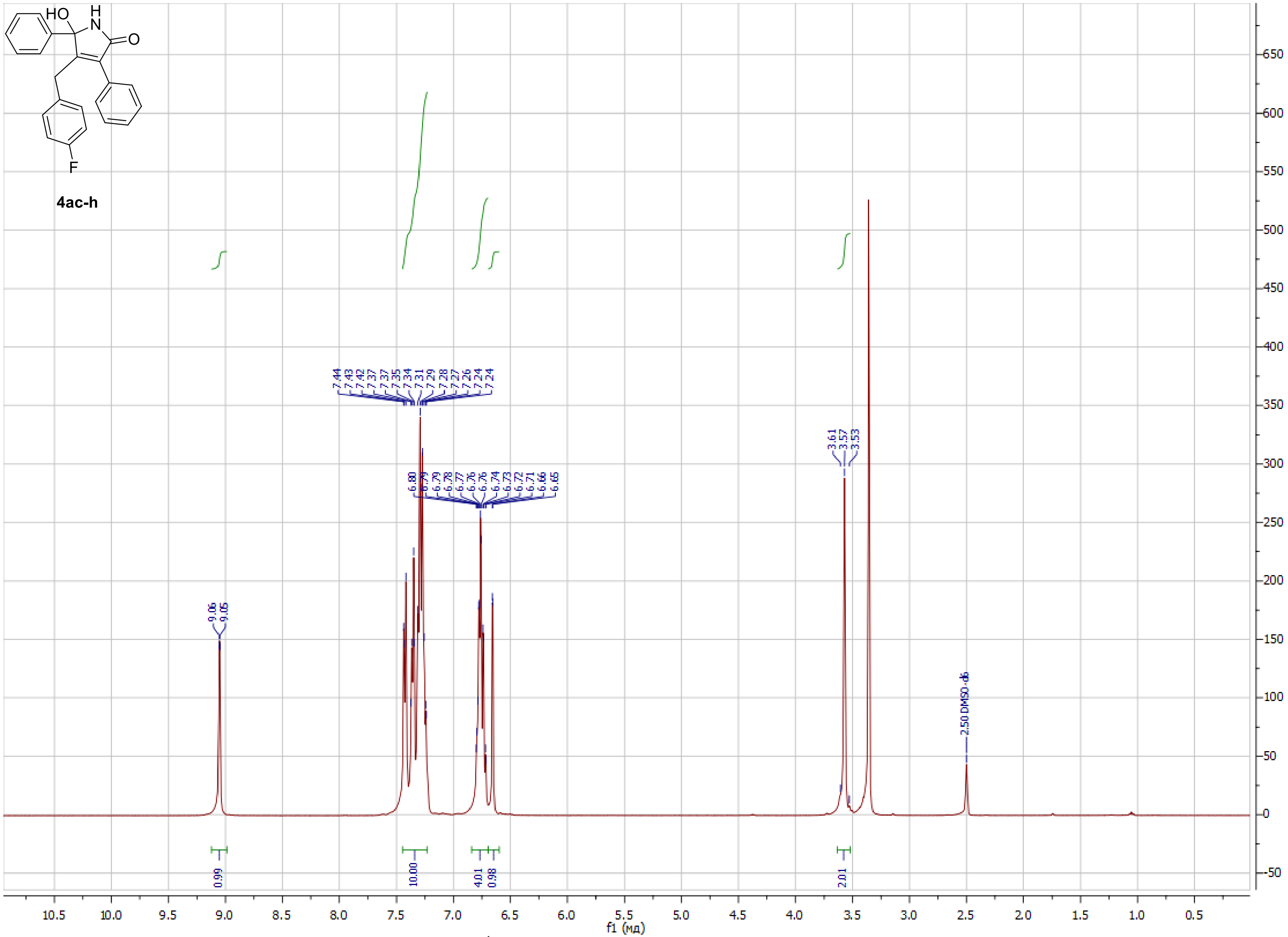

Figure S30. ^{1}H NMR spectrum of **4ac-h** in DMSO-d_6 (400 MHz)

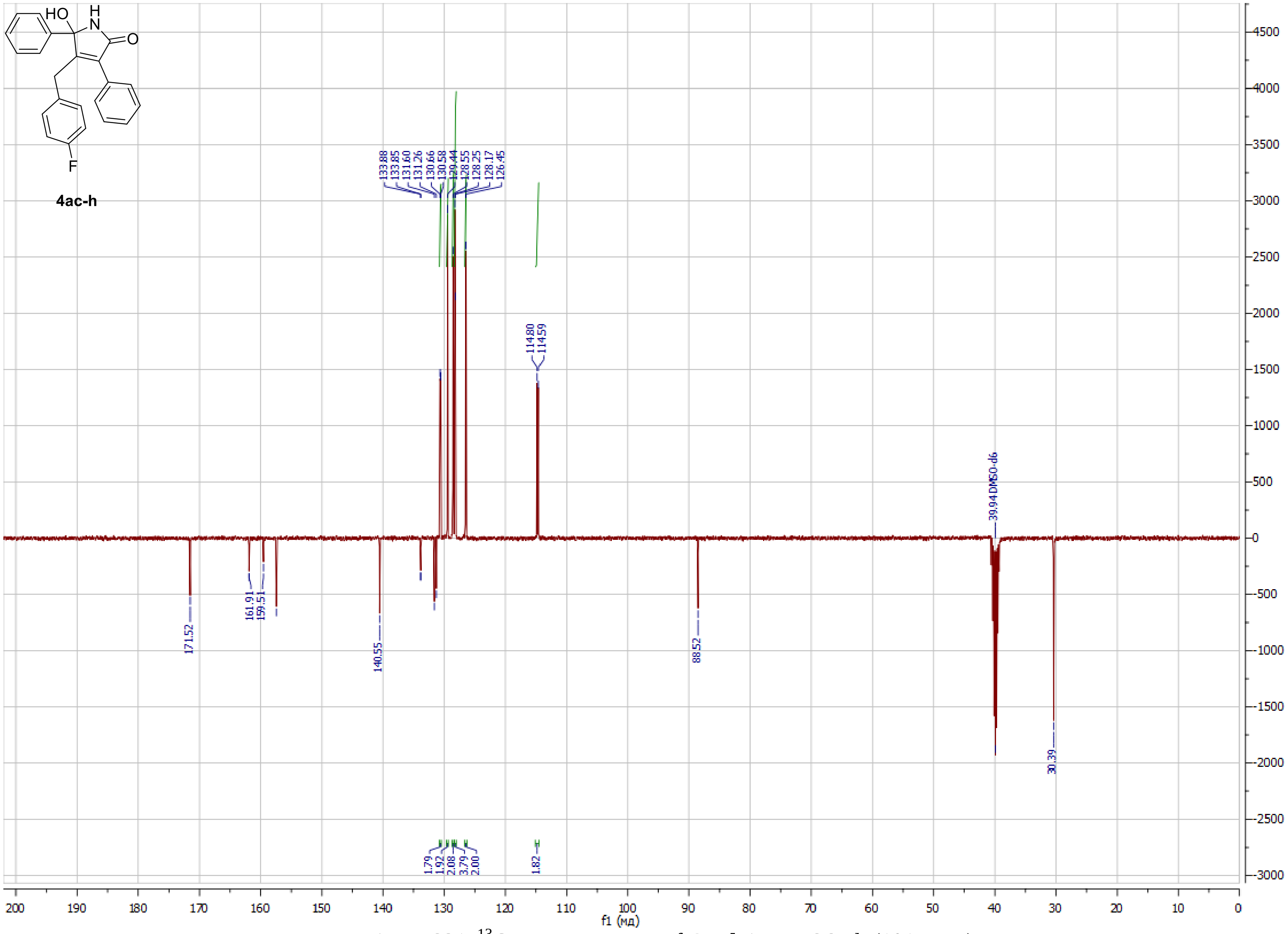

Figure S31. ^{13}C NMR spectrum of **4ac-h** in DMSO-d_6 (101 MHz)

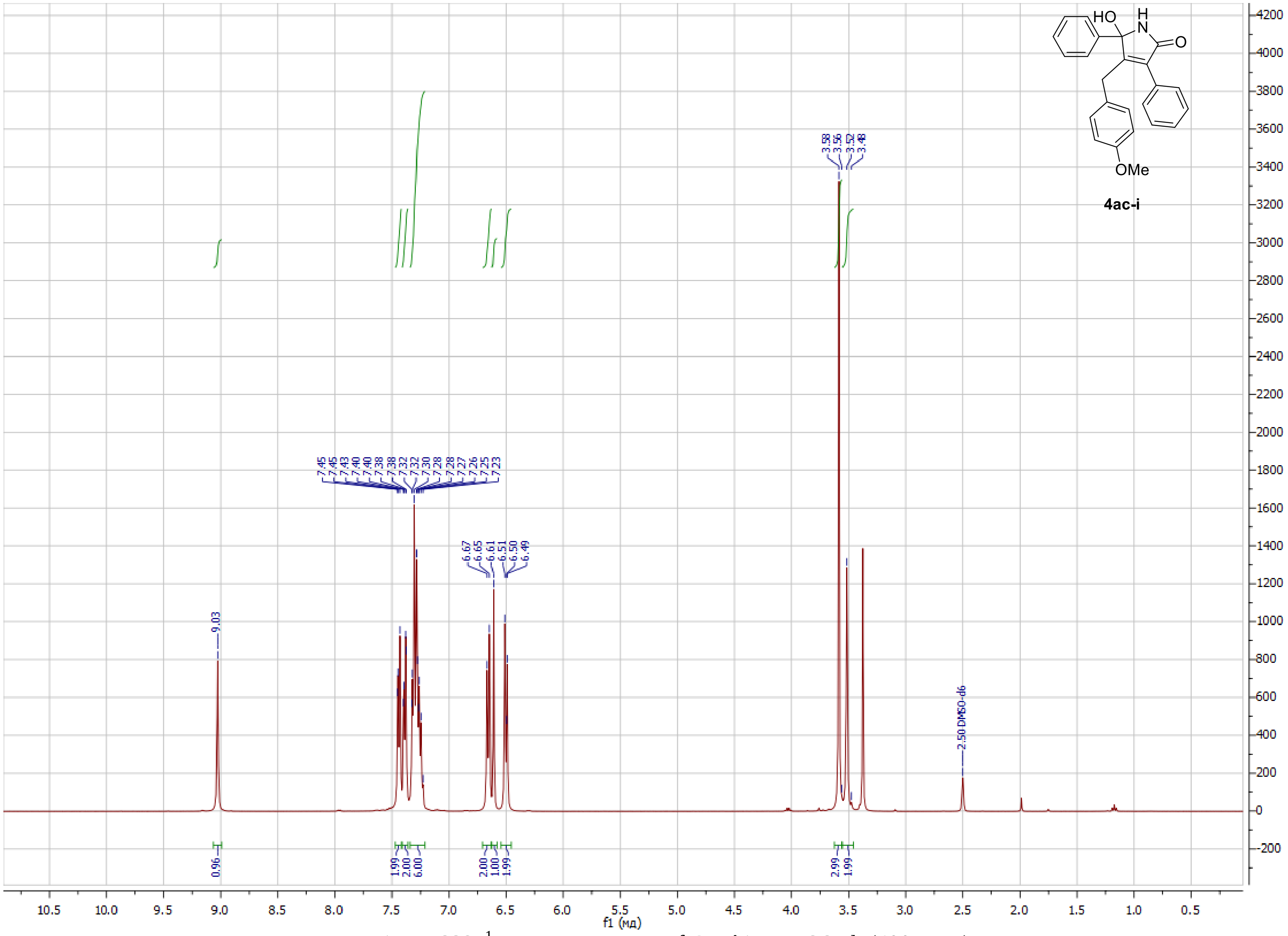

Figure S32. ^{1}H NMR spectrum of **4ac-i** in DMSO-d_6 (400 MHz)

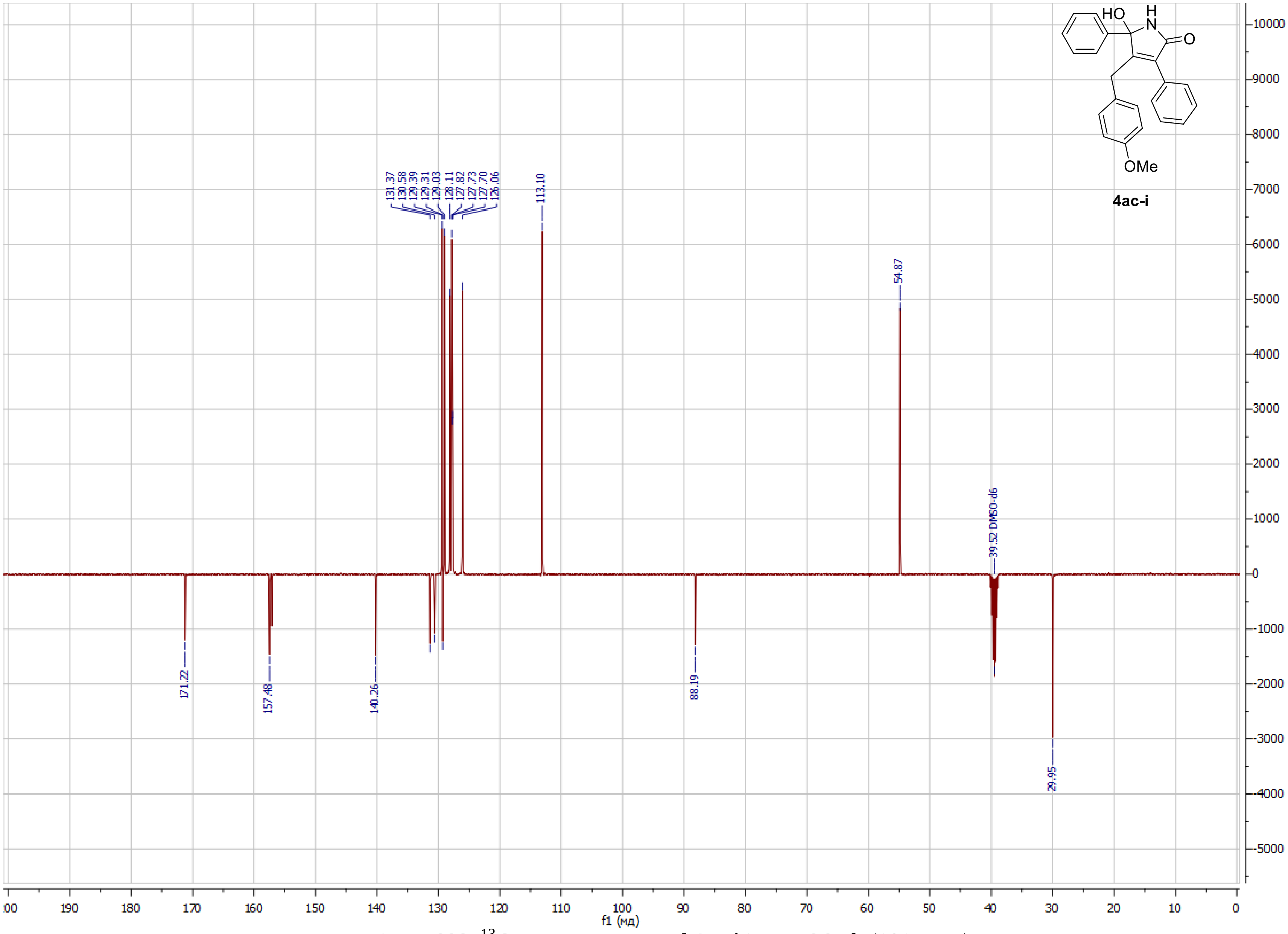

Figure S33. ^{13}C NMR spectrum of **4ac-i** in DMSO-d_6 (101 MHz)

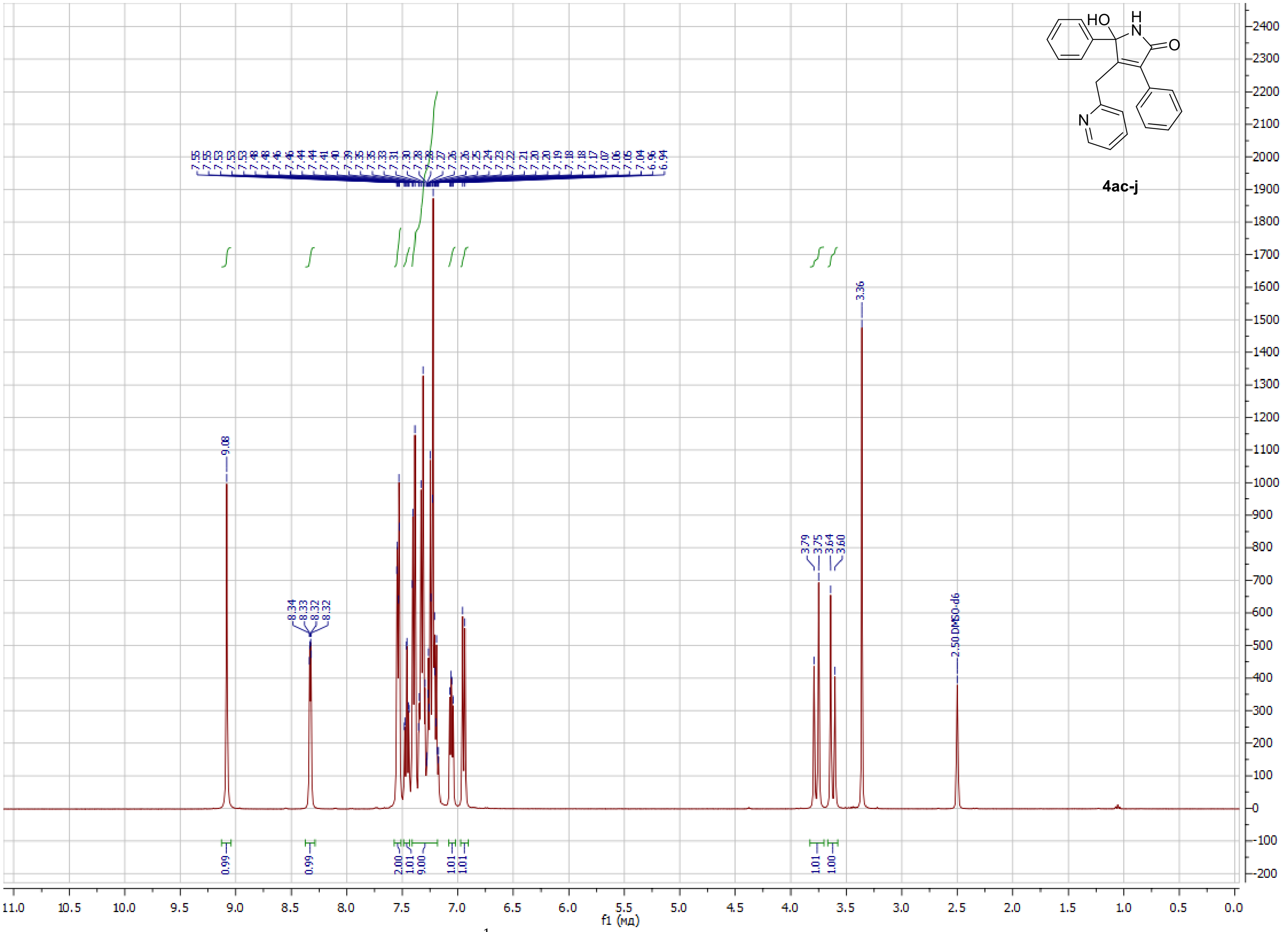

Figure S34. ^{1}H NMR spectrum of **4ac-j** in DMSO-d_6 (400 MHz)

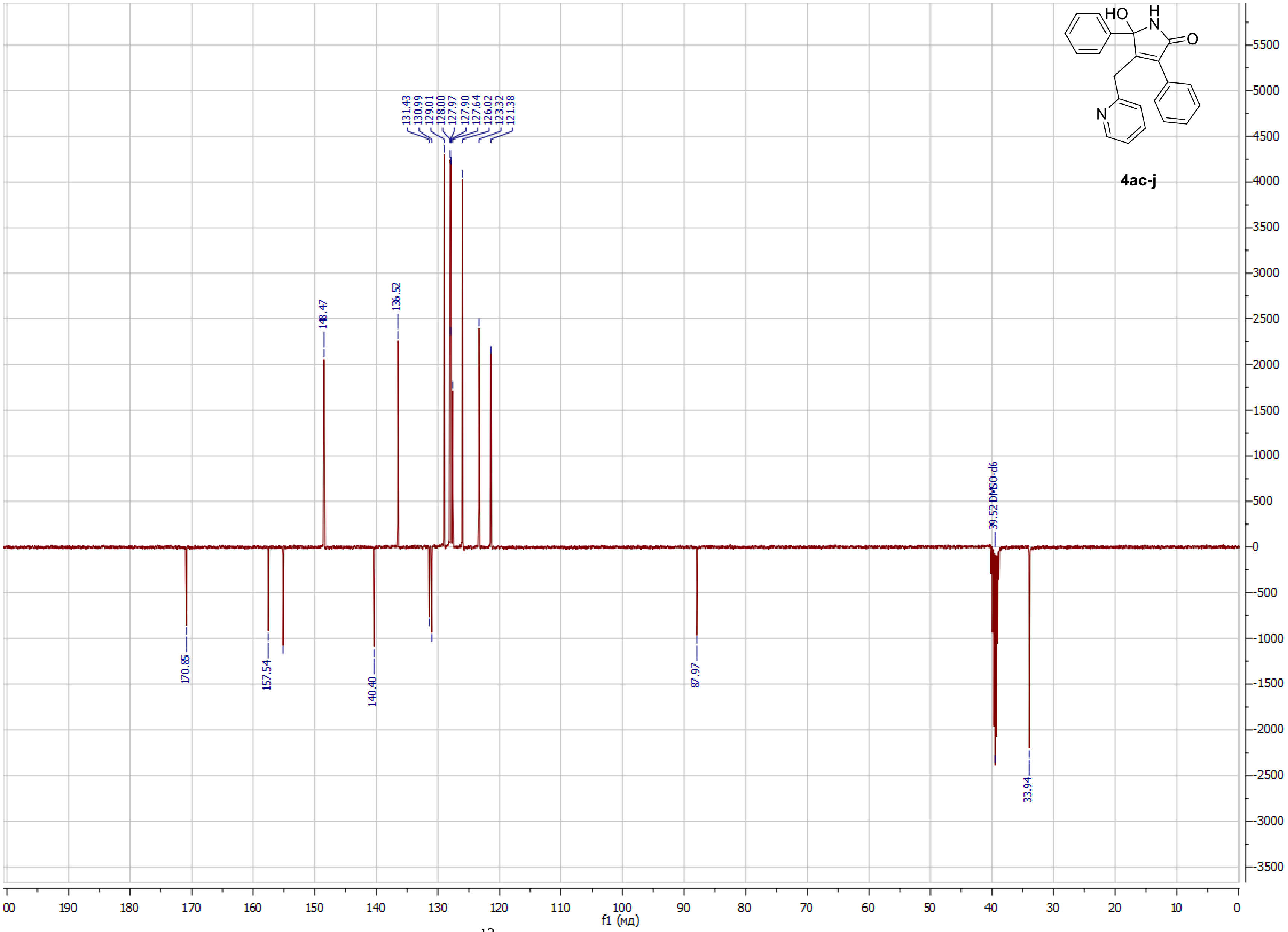

Figure S35. ^{13}C NMR spectrum of **4ac-j** in DMSO-d_6 (101 MHz)

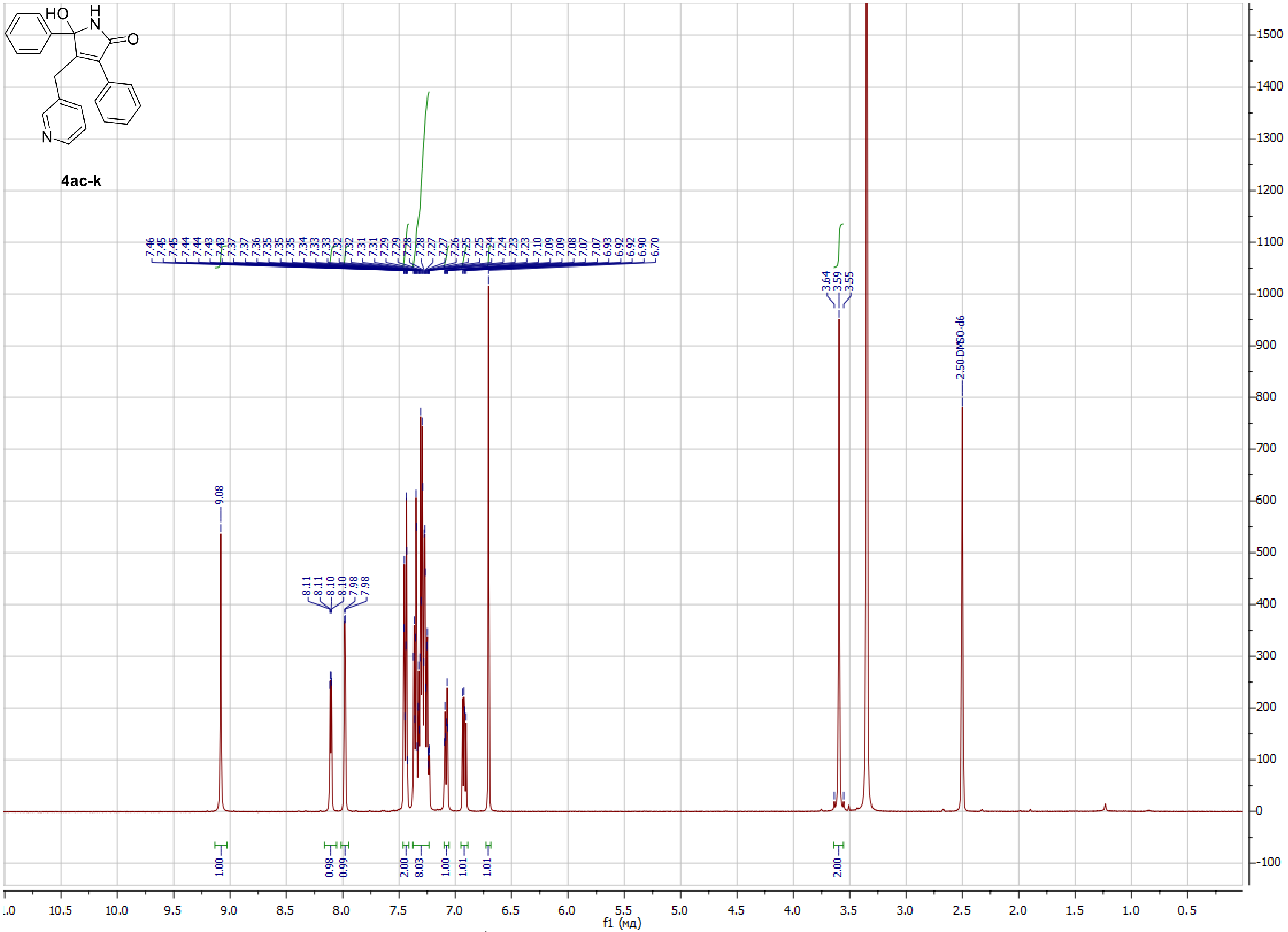

Figure S36. ^{1}H NMR spectrum of **4ac-k** in DMSO-d_6 (400 MHz)

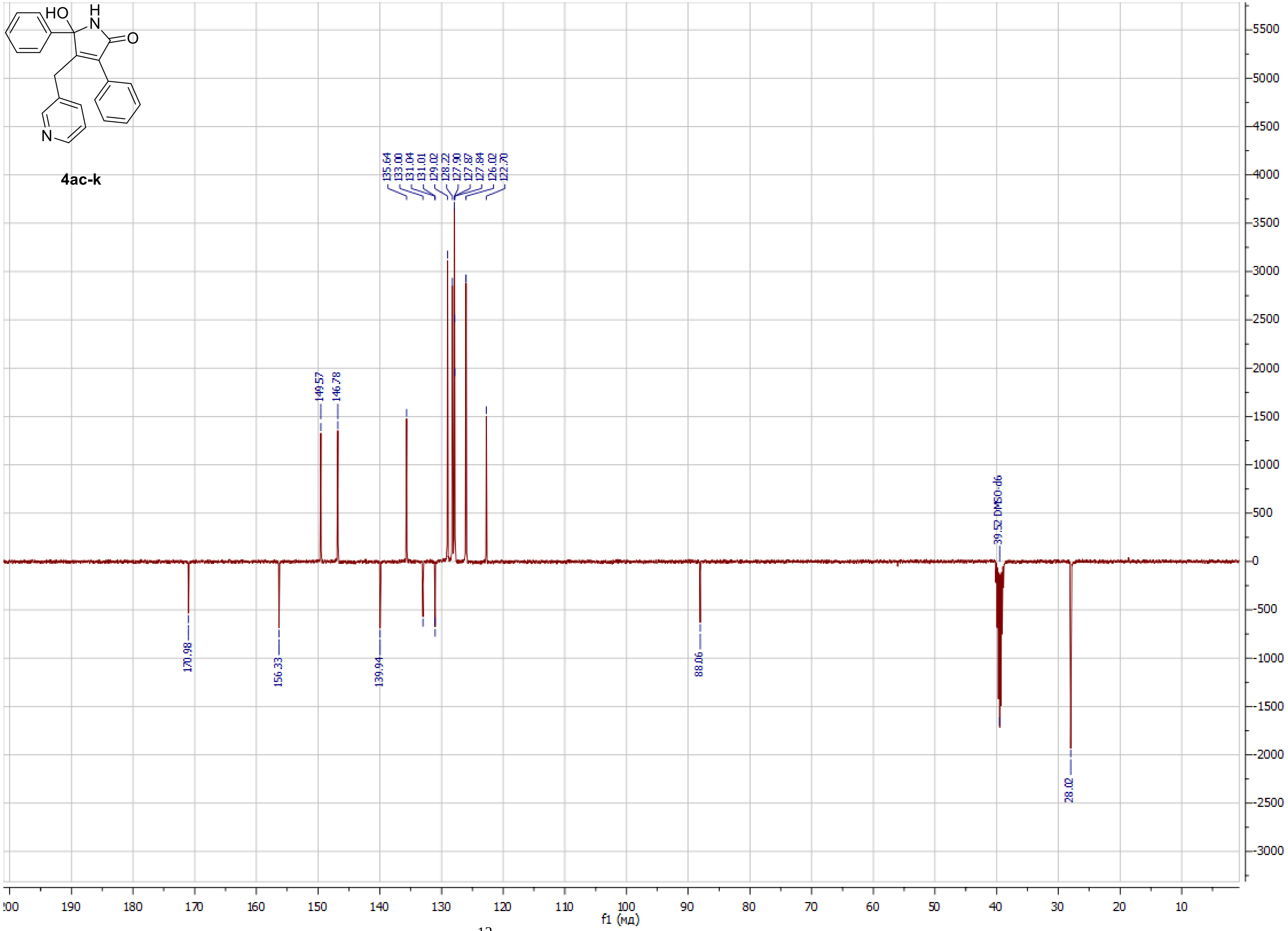

Figure S37. ^{13}C NMR spectrum of **4ac-k** in DMSO-d_6 (101 MHz)

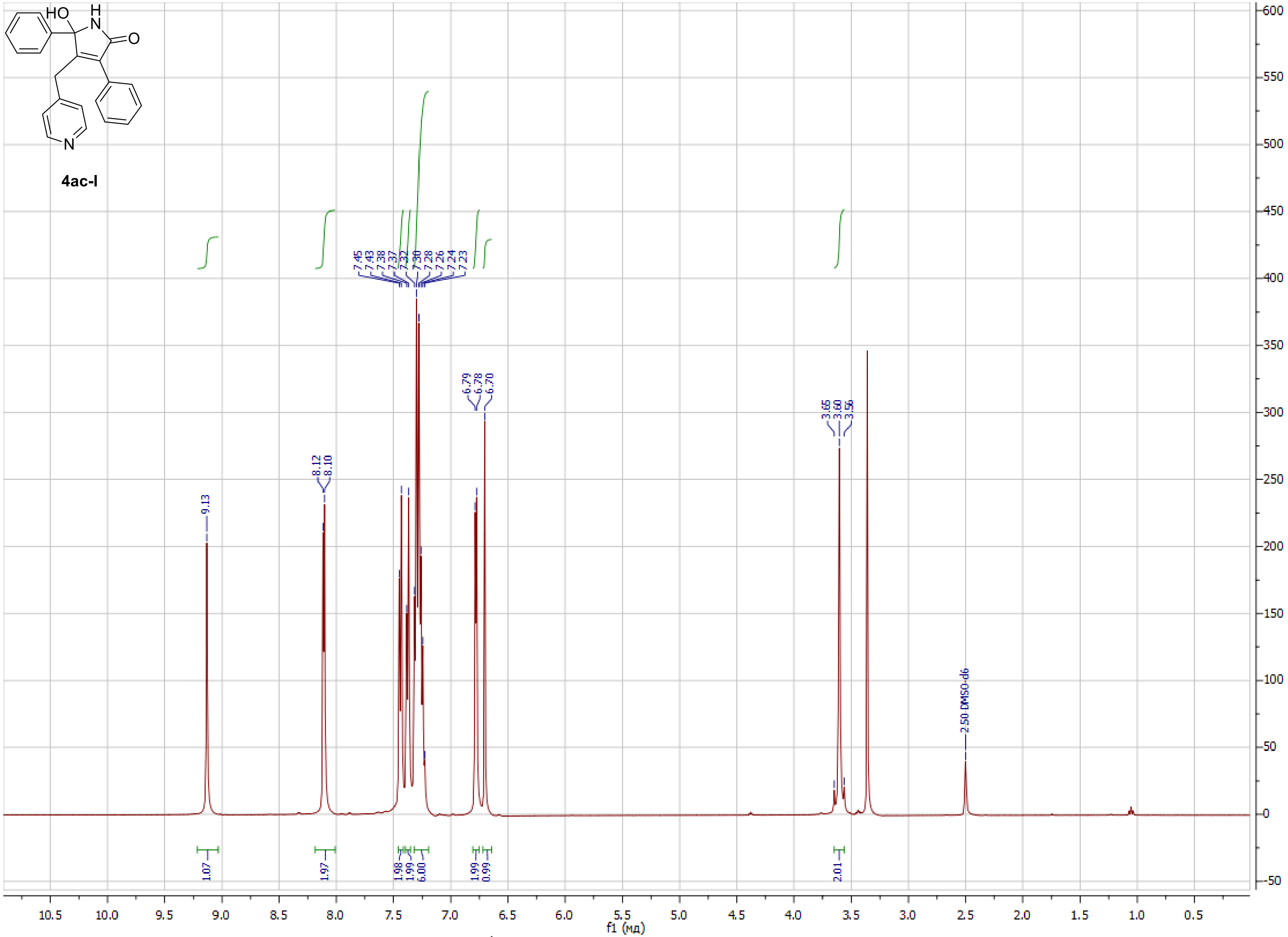

Figure S38. ^{1}H NMR spectrum of **4ac-l** in DMSO-d_6 (400 MHz)

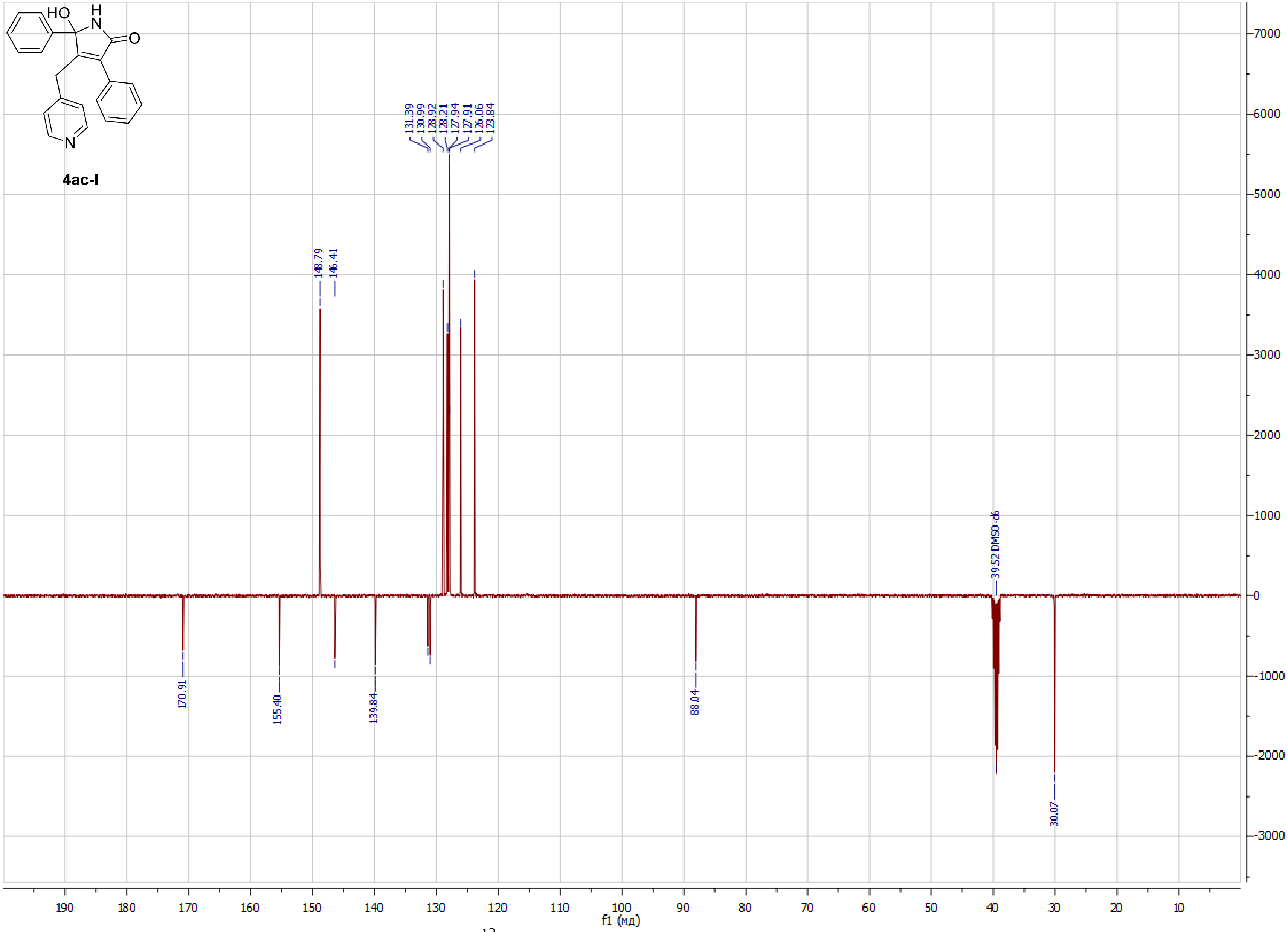

Figure S39. ^{13}C NMR spectrum of **4ac-l** in DMSO-d_6 (101 MHz)

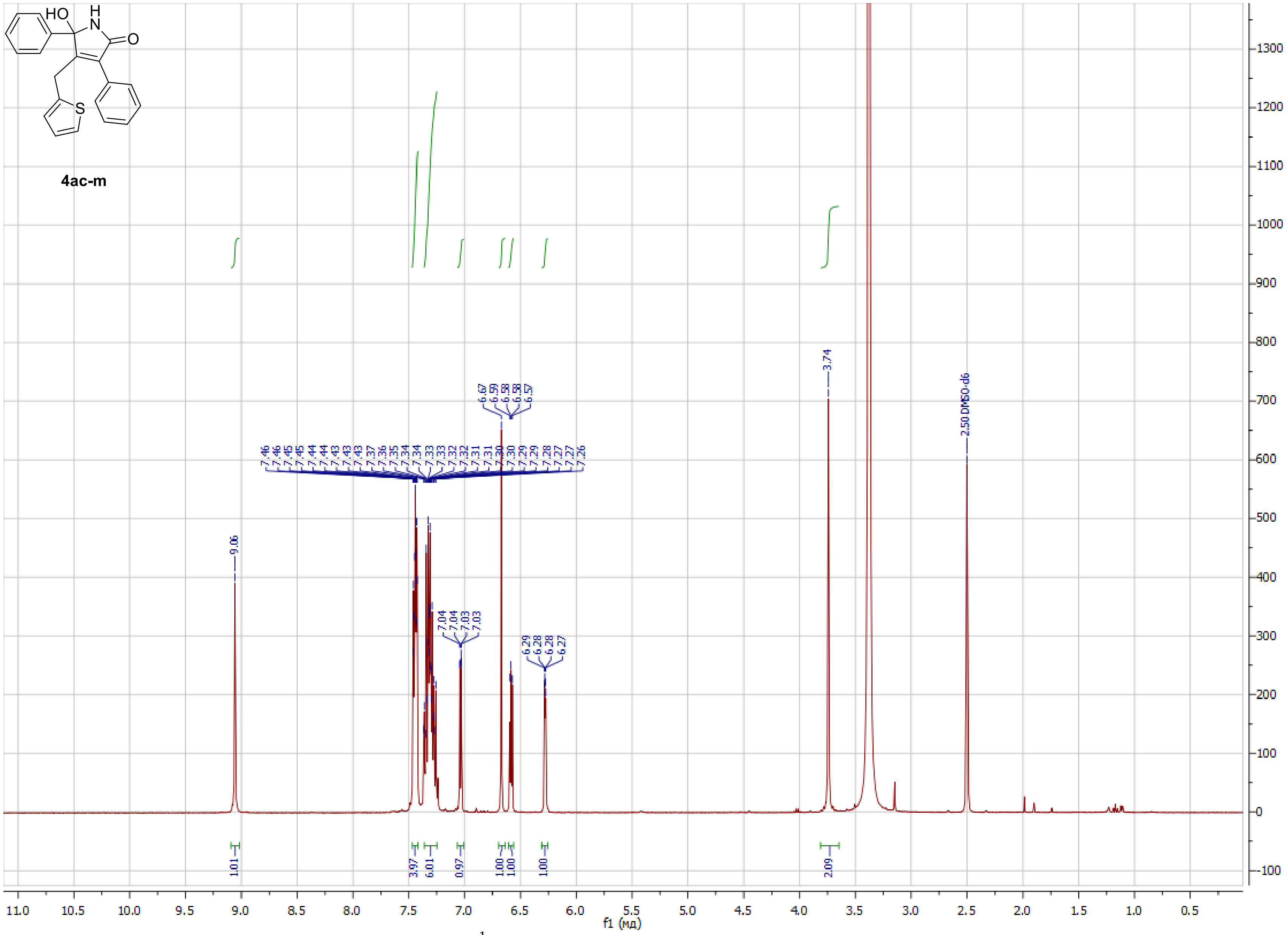

Figure S40. ^{1}H NMR spectrum of **4ac-m** in DMSO-d_6 (400 MHz)

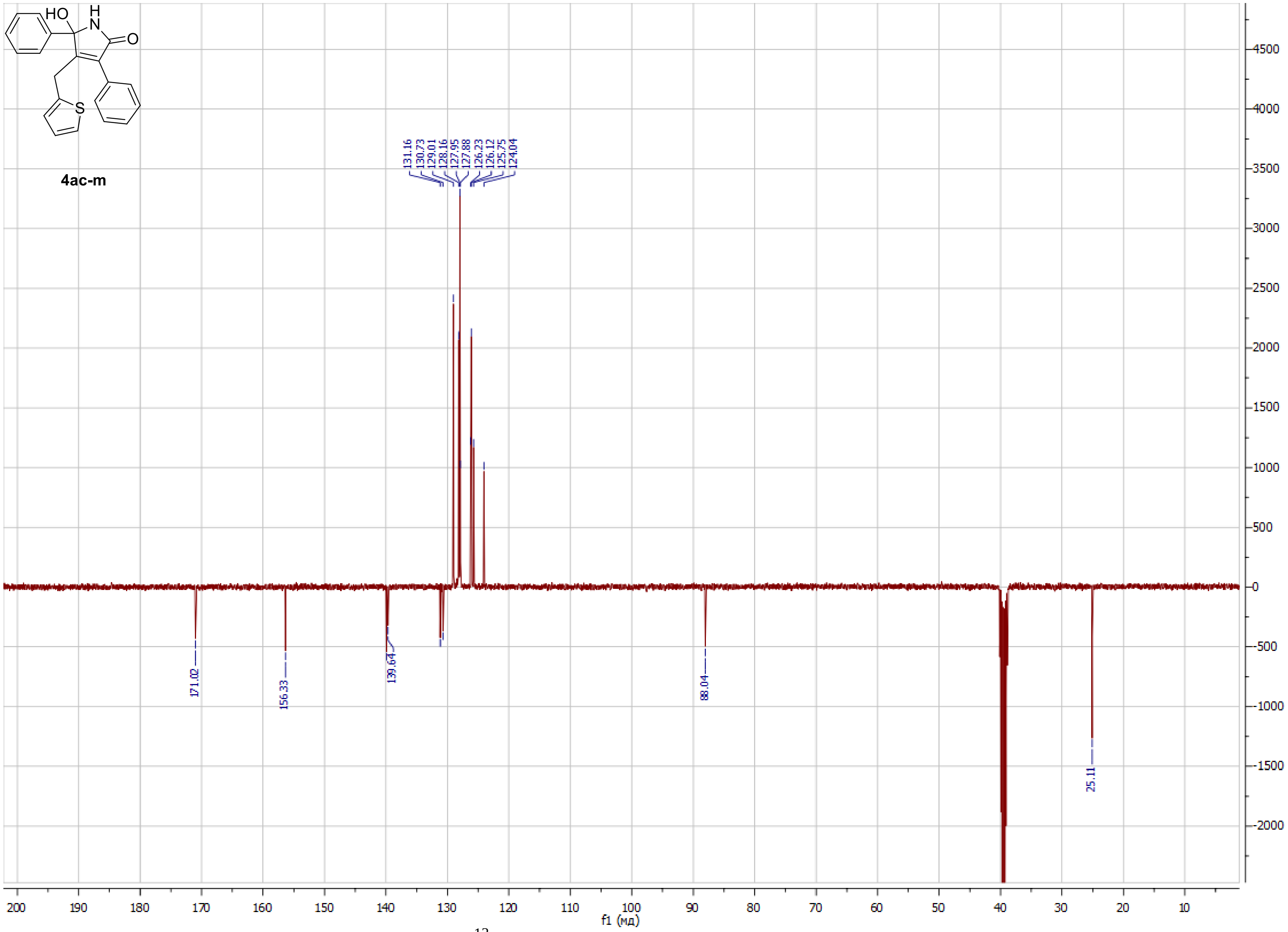

Figure S41. ^{13}C NMR spectrum of **4ac-m** in DMSO-d_6 (101 MHz)

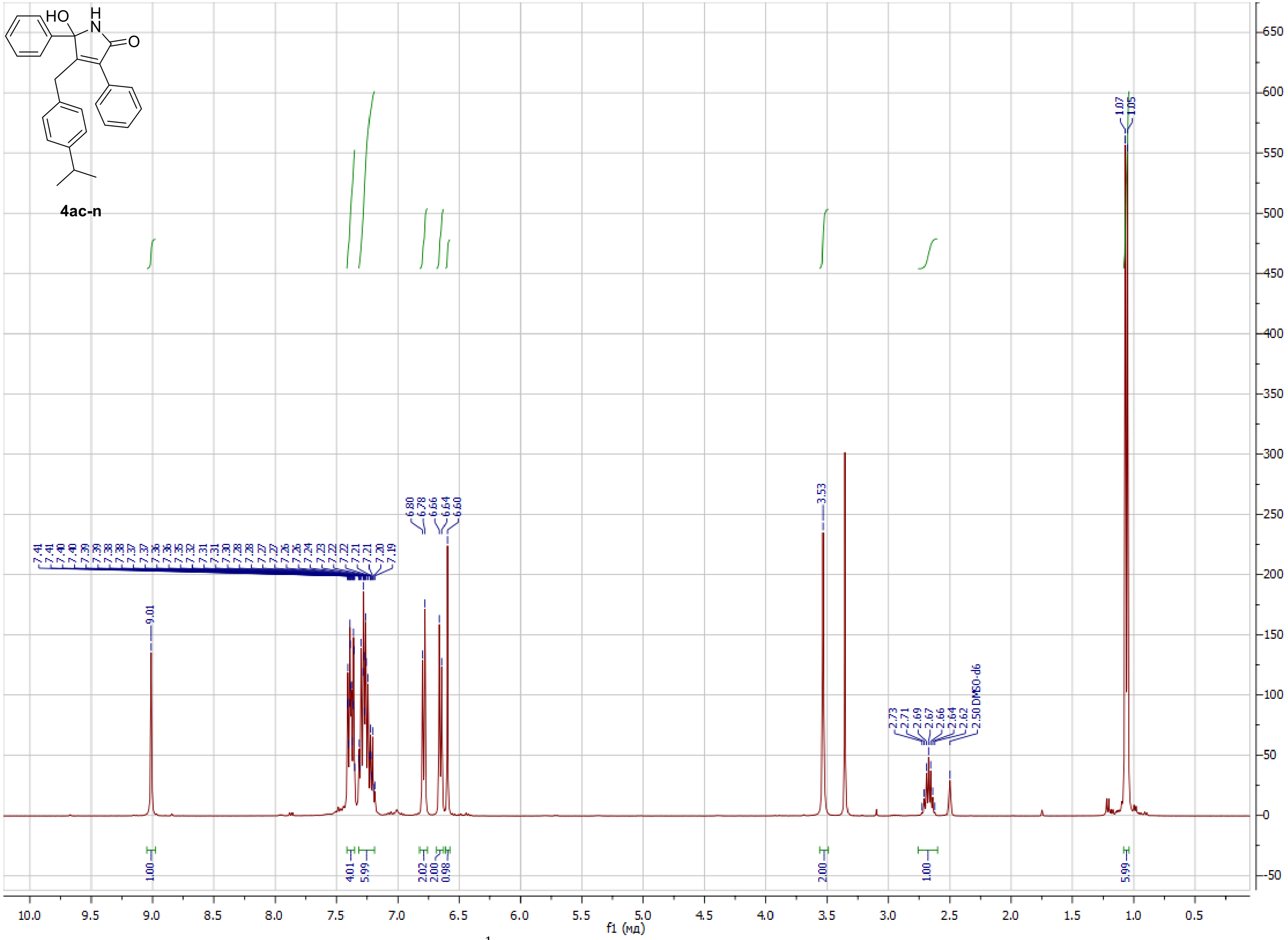

Figure S42. ^{1}H NMR spectrum of **4ac-n** in DMSO-d_6 (400 MHz)

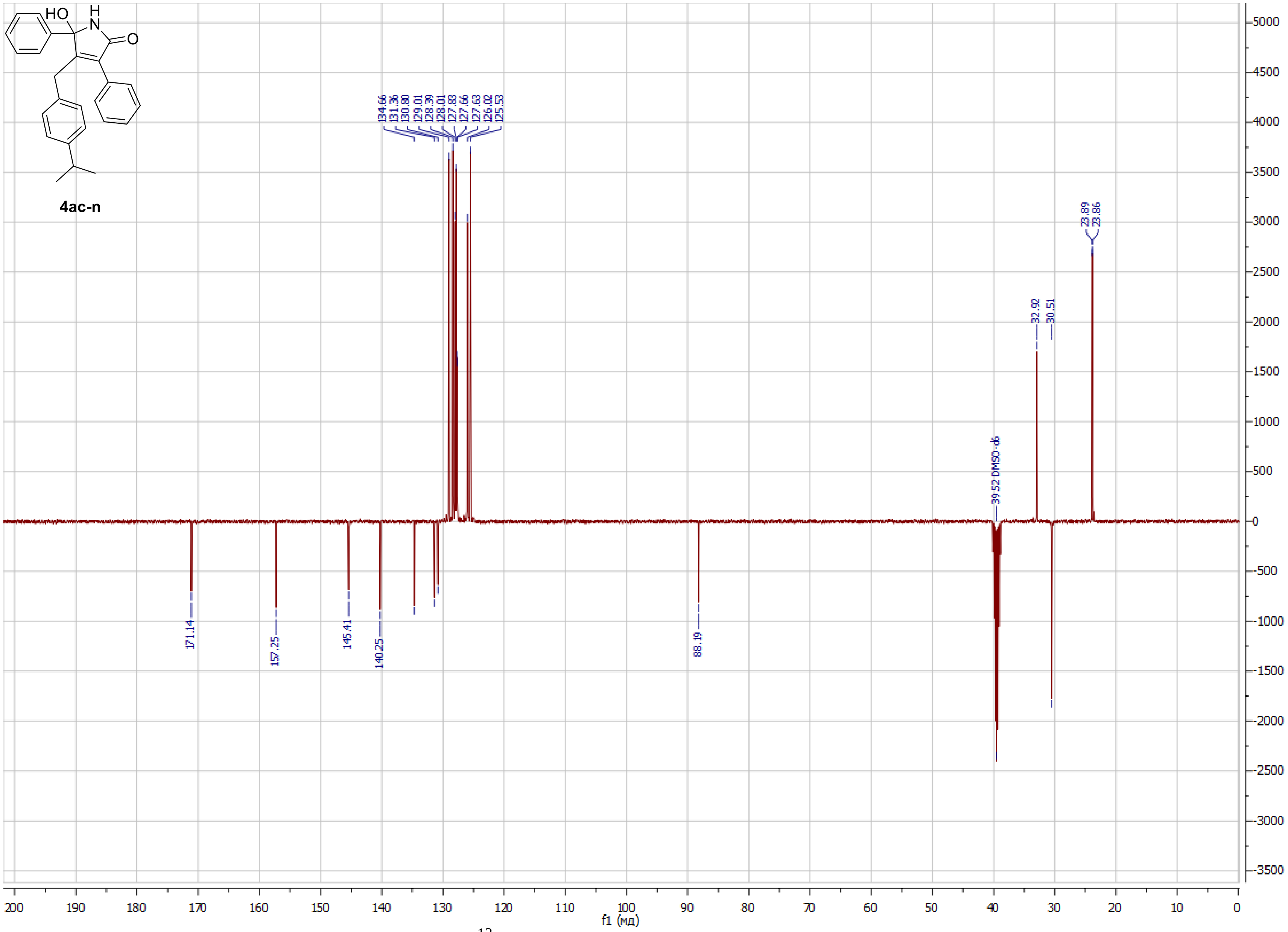

Figure S43. ^{13}C NMR spectrum of **4ac-n** in DMSO-d_6 (101 MHz)

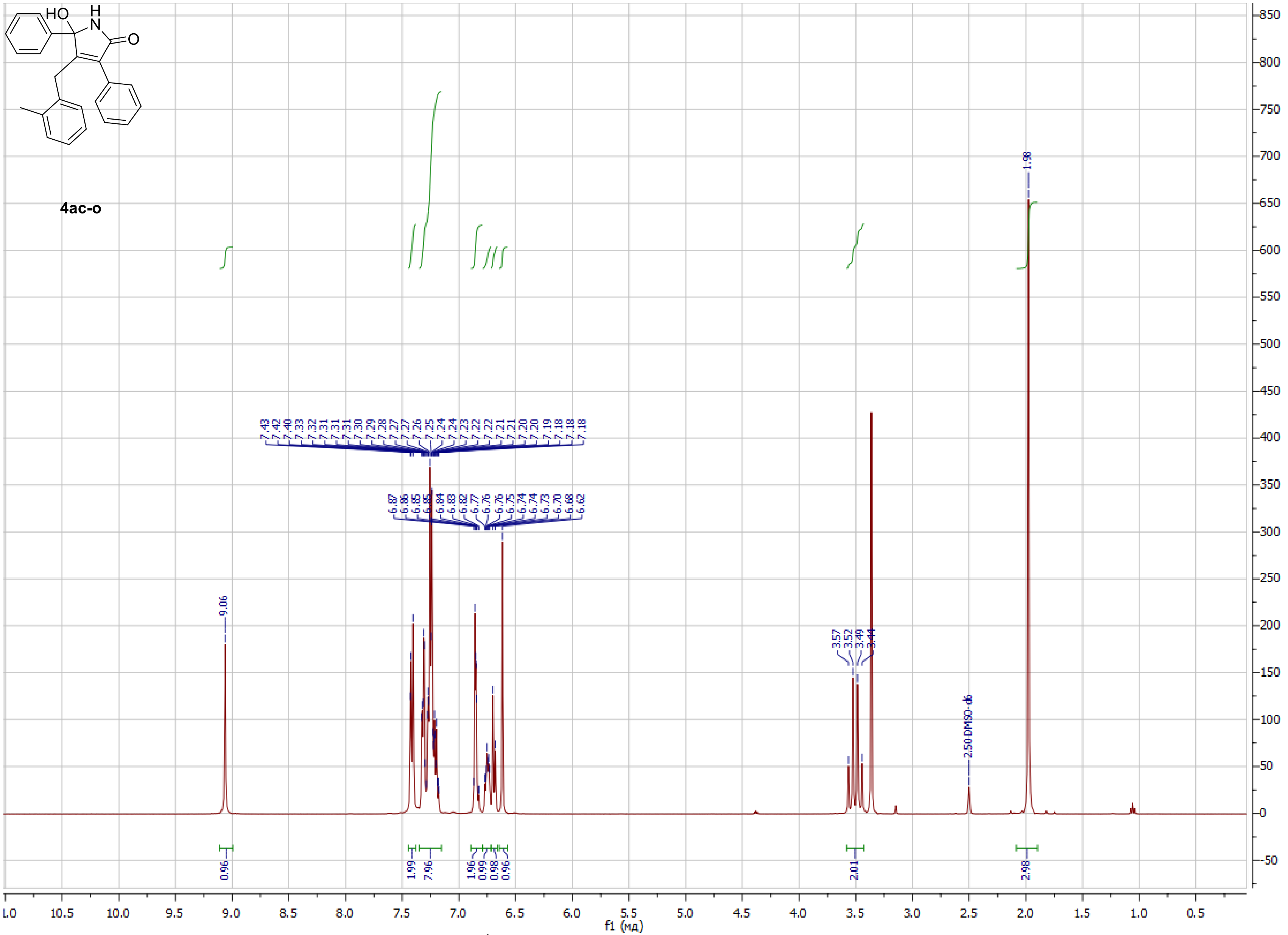

Figure S44. ^{1}H NMR spectrum of **4ac-o** in DMSO-d_6 (400 MHz)

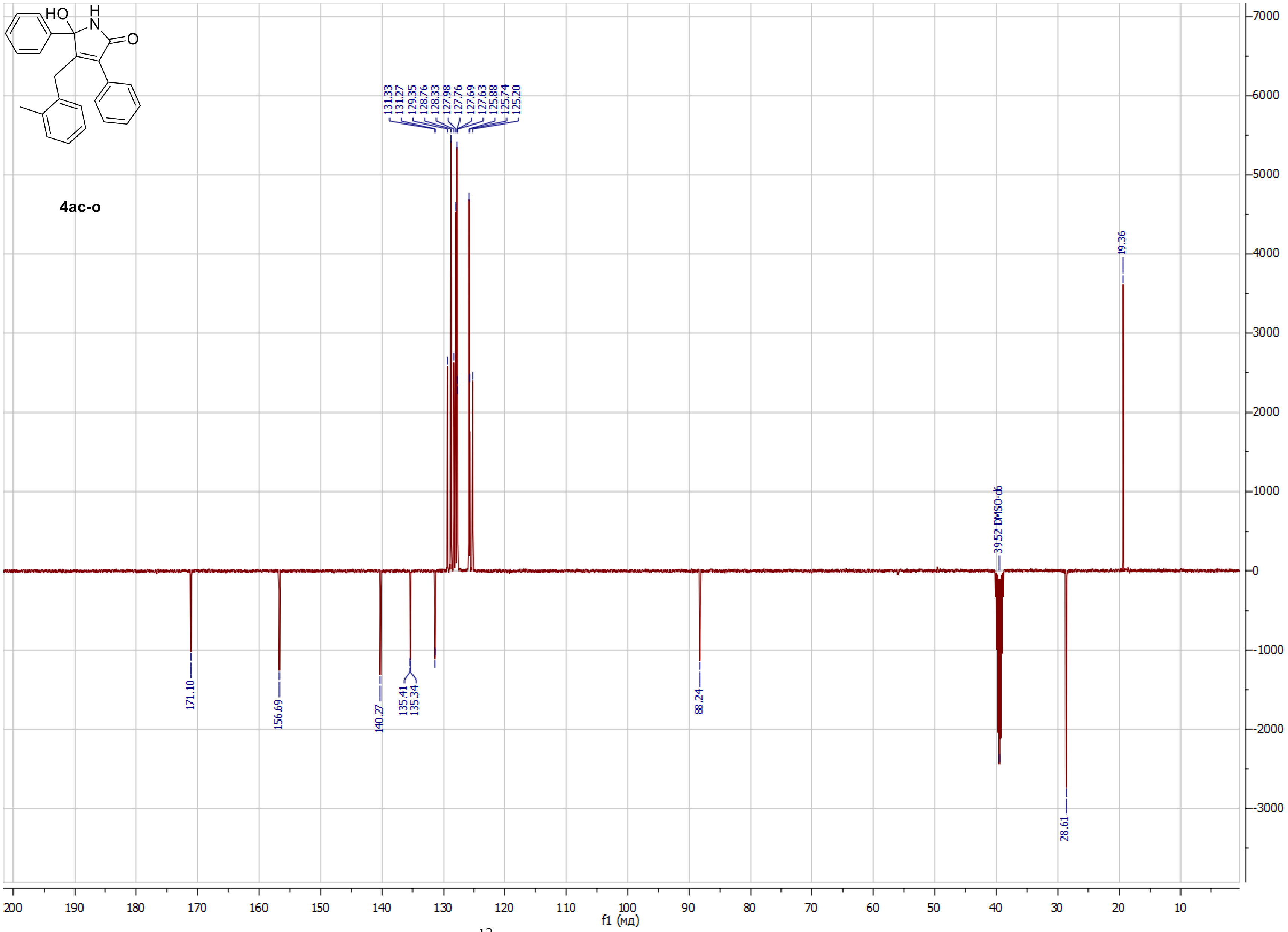

Figure S45. ^{13}C NMR spectrum of **4ac-o** in DMSO-d_6 (101 MHz)

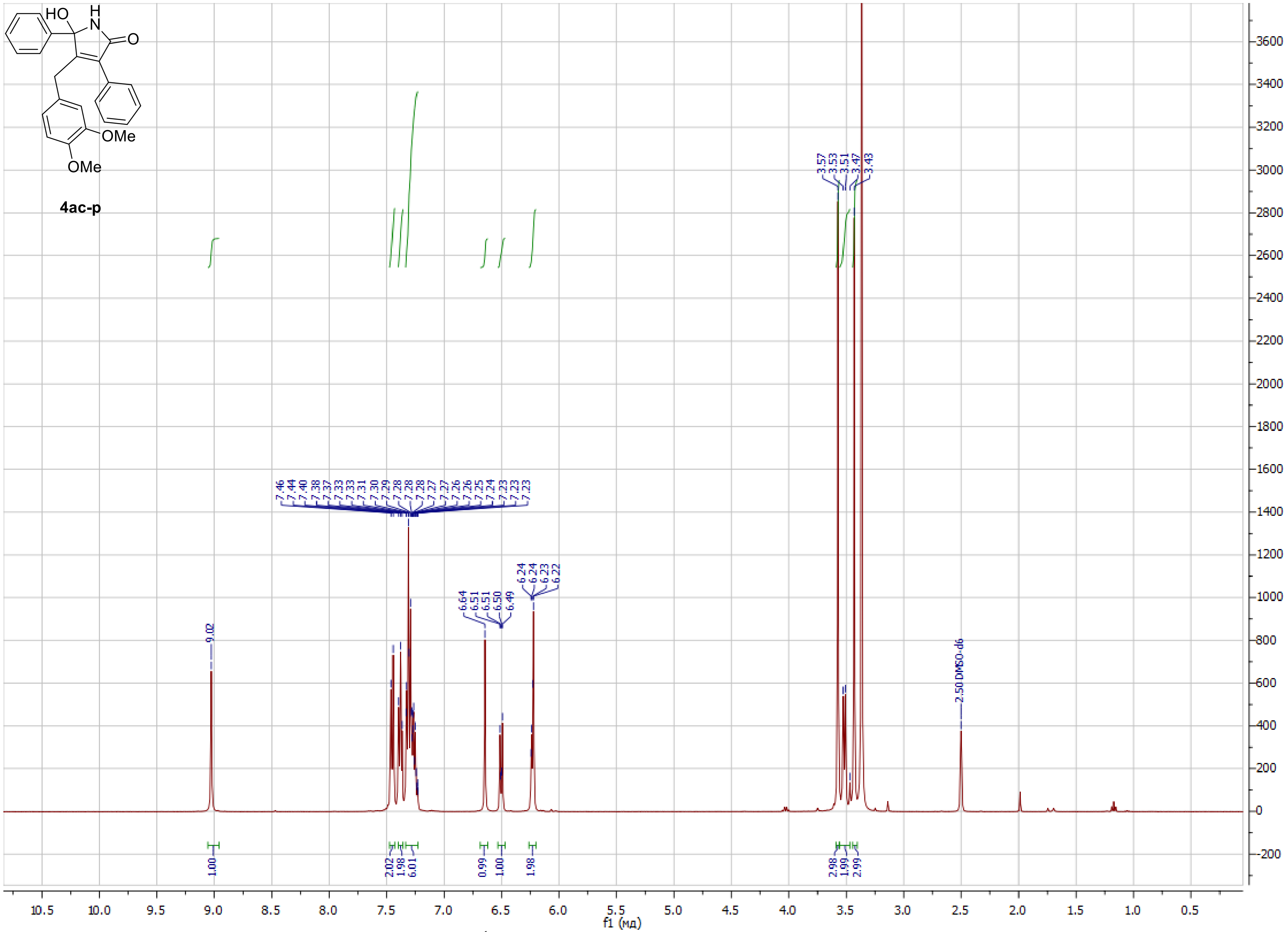

Figure S46. ^{1}H NMR spectrum of **4ac-p** in DMSO-d_6 (400 MHz)

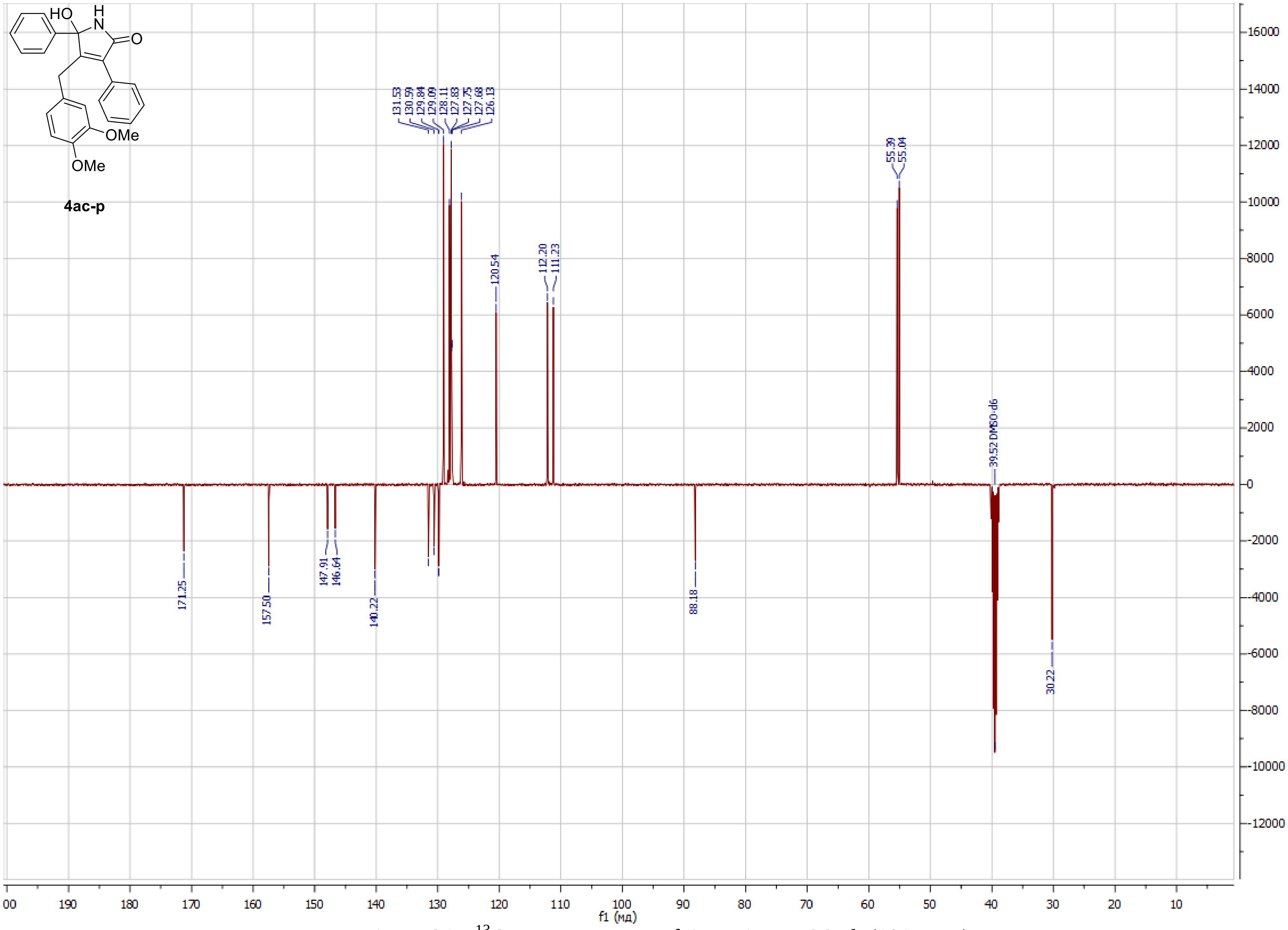

Figure S47. ^{13}C NMR spectrum of **4ac-p** in DMSO-d_6 (101 MHz)

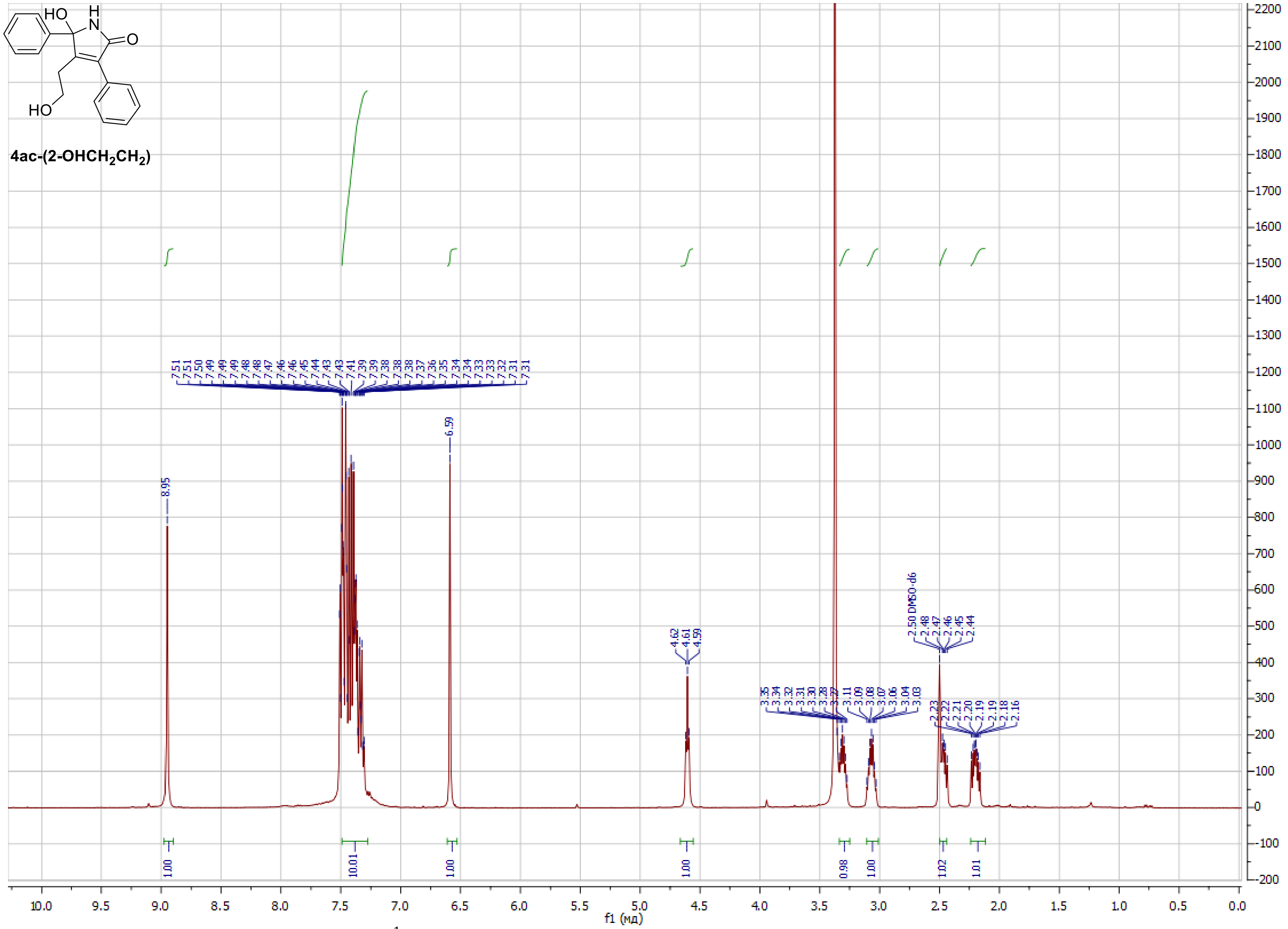

Figure S48. ^{1}H NMR spectrum of **4ac-(2OHCH$_2$CH$_2$)** in DMSO-d_6 (400 MHz)

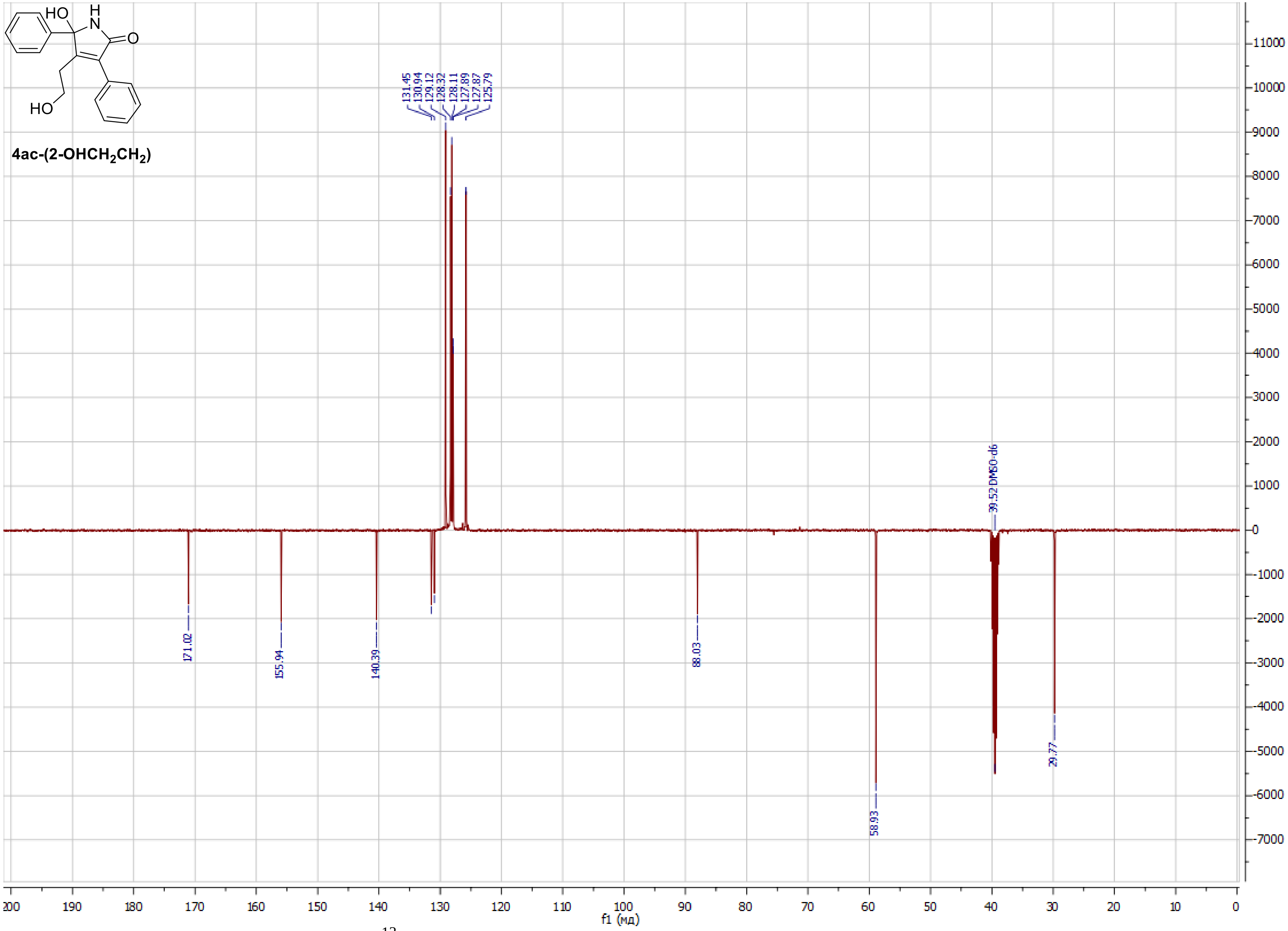

Figure S49. ^{13}C NMR spectrum of **4ac-(2OHCH$_2$CH$_2$)** in DMSO-d_6 (101 MHz)

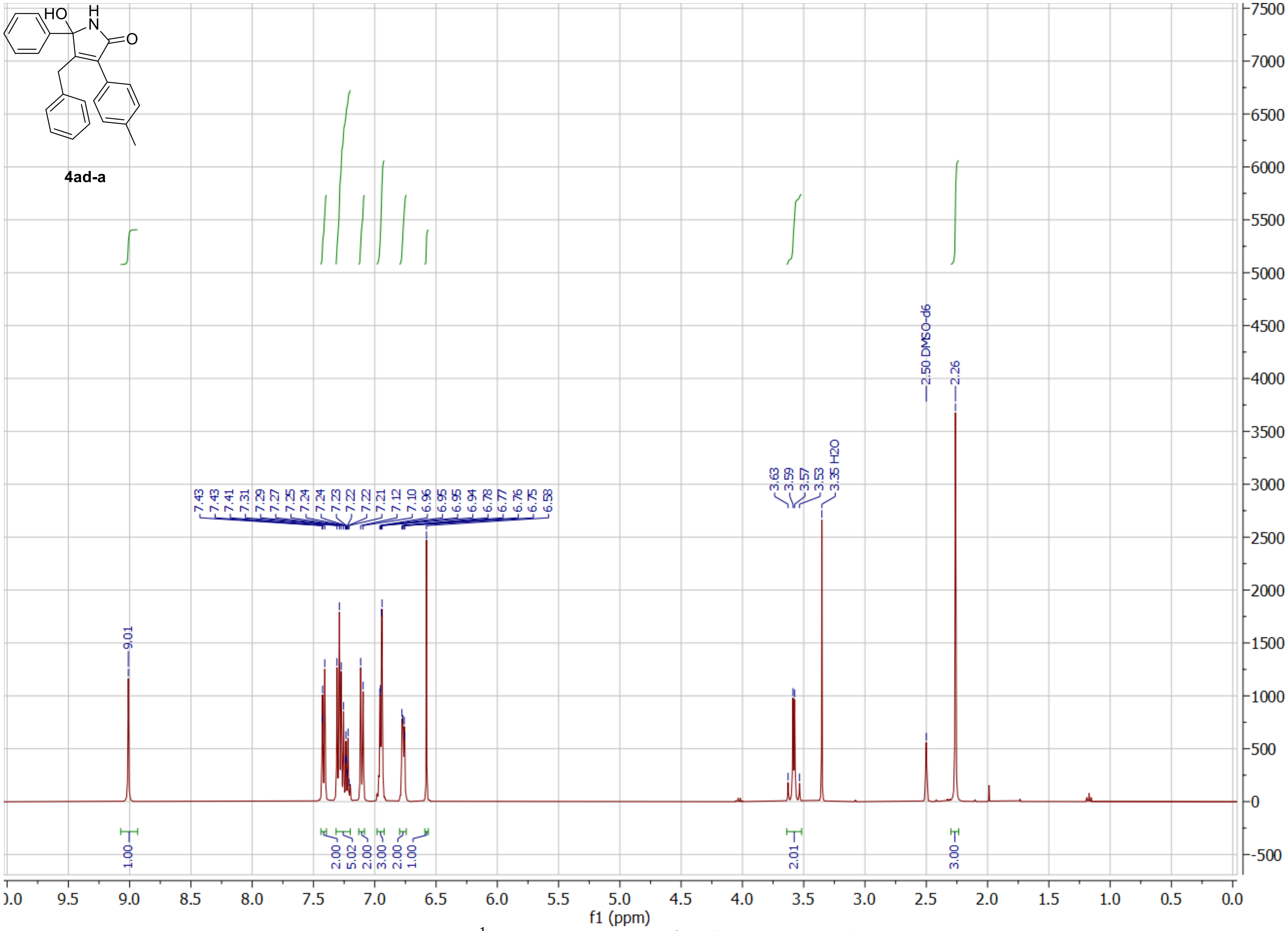

Figure S50. ^{1}H NMR spectrum of **4ad-a** in DMSO-d_6 (400 MHz)

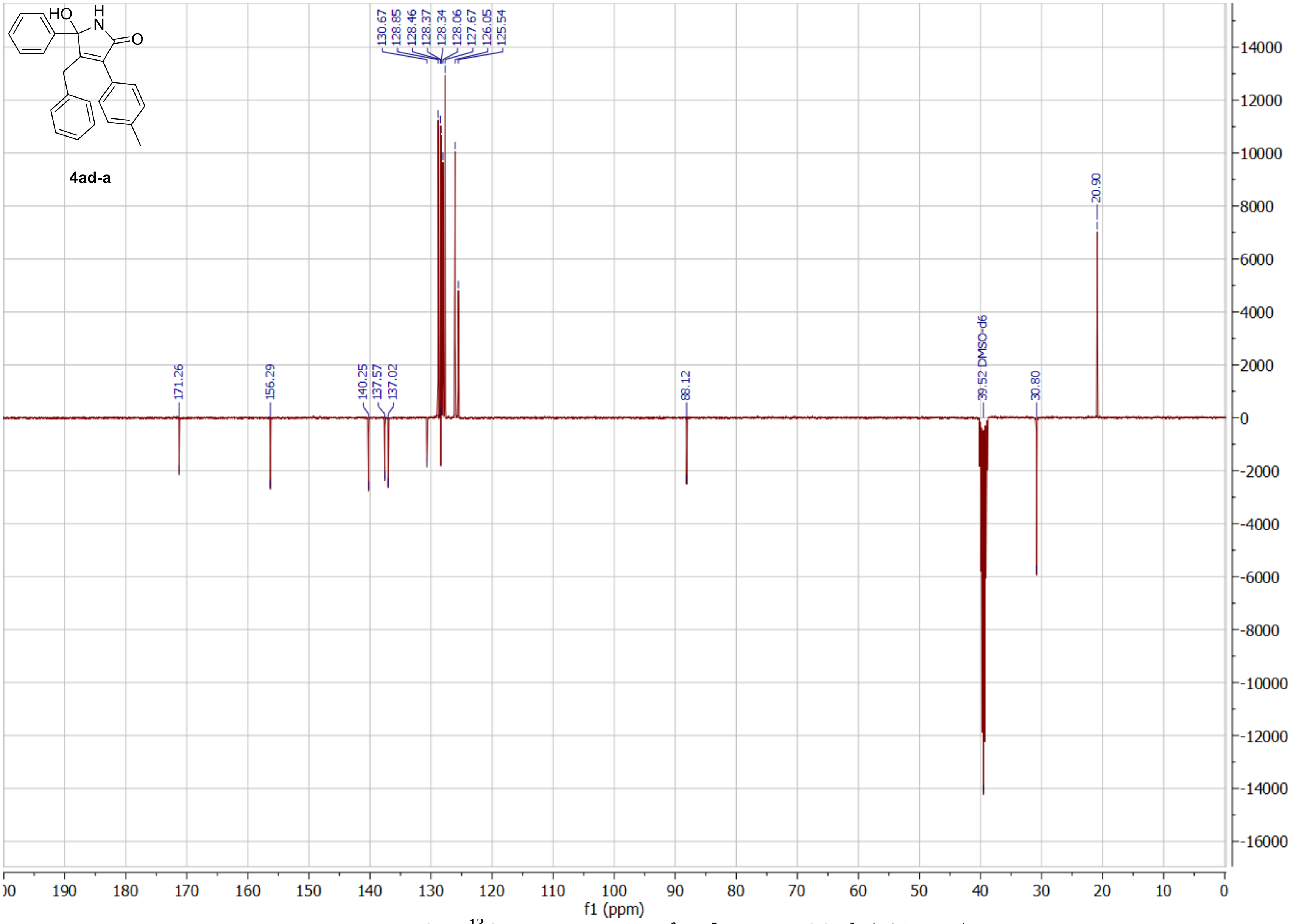

Figure S51. ^{13}C NMR spectrum of **4ad-a** in DMSO-d_6 (101 MHz)

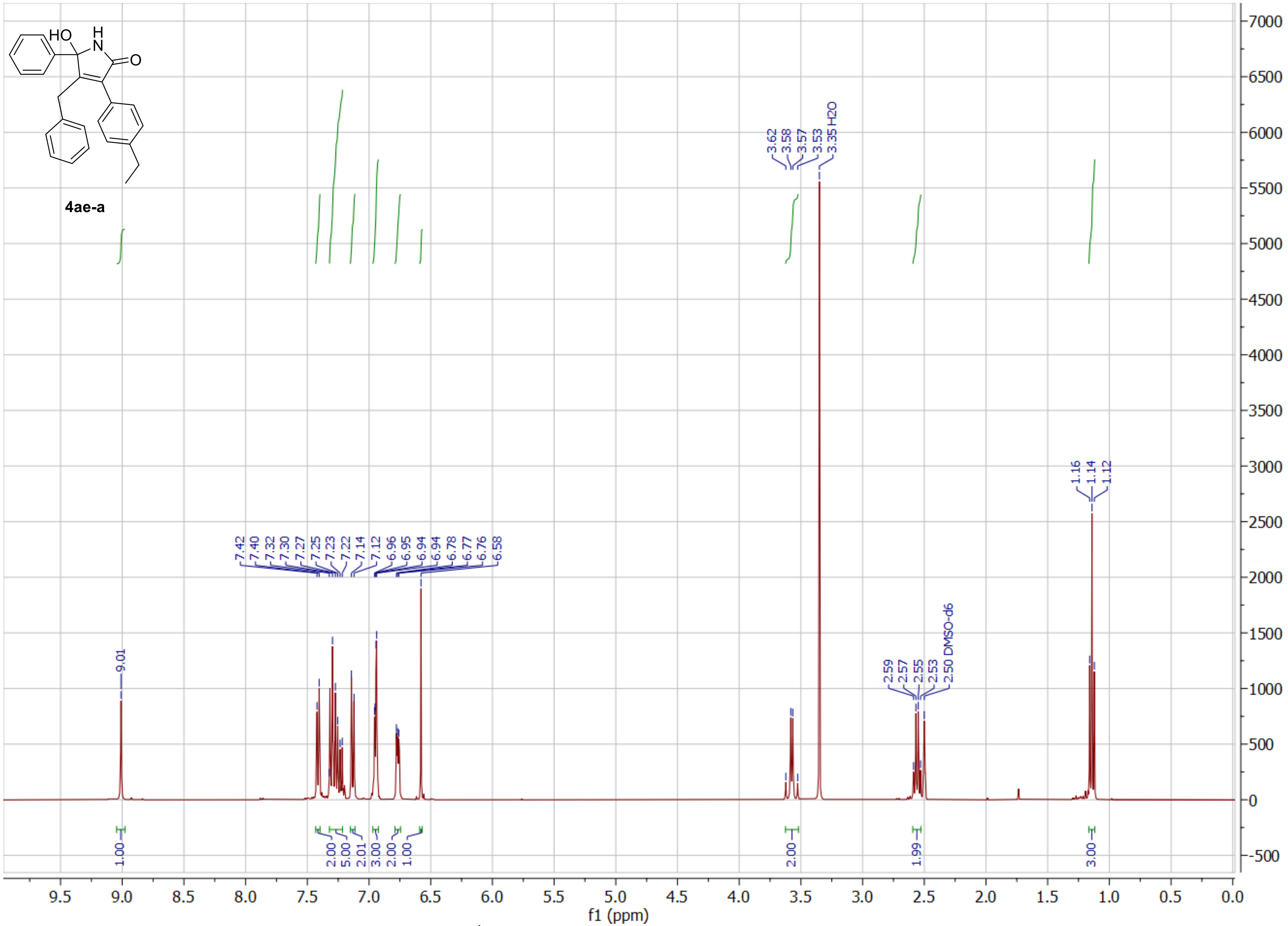

Figure S52. ^{1}H NMR spectrum of **4ae-a** in DMSO-d_6 (400 MHz)

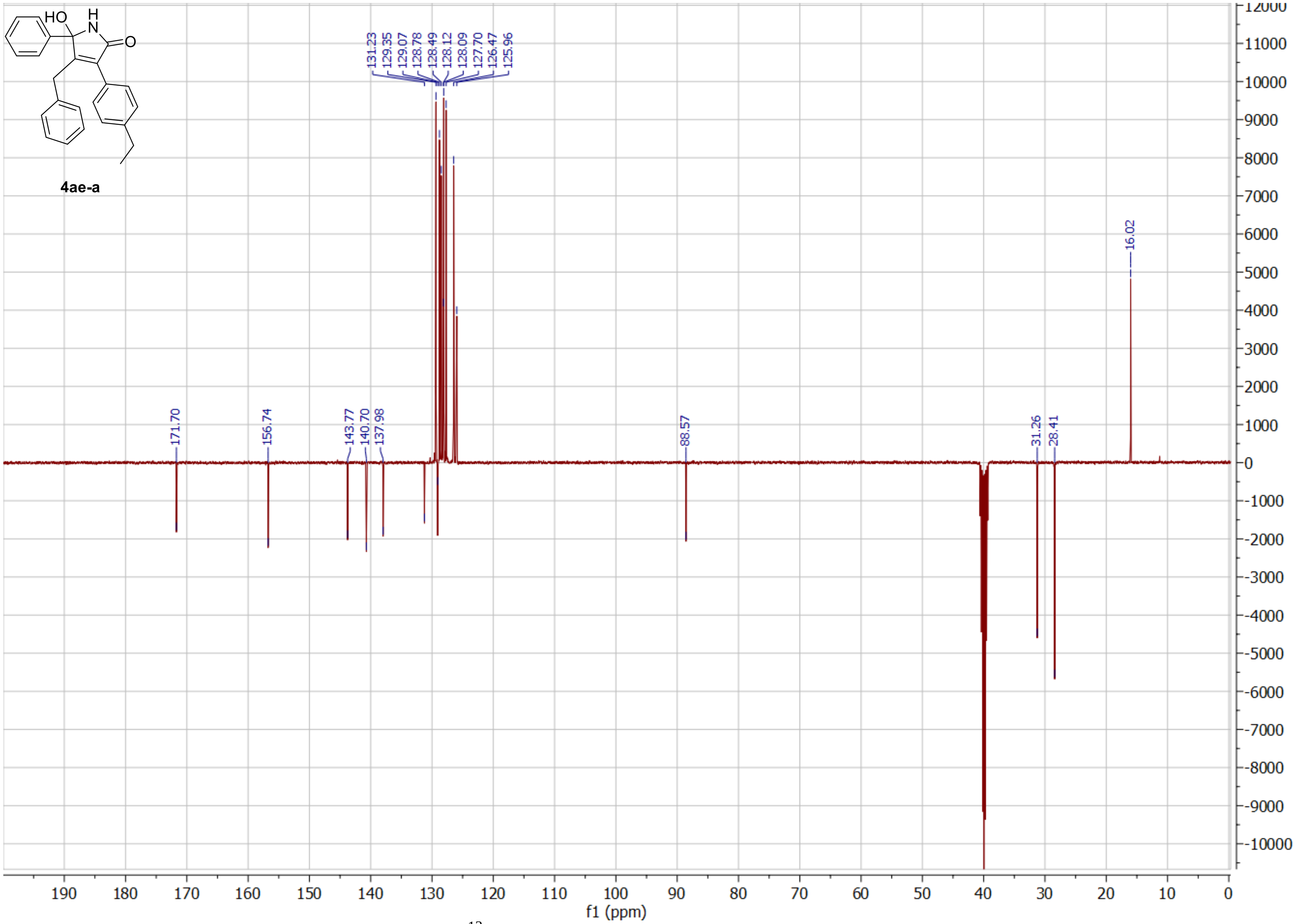

Figure S53. ^{13}C NMR spectrum of **4ae-a** in DMSO-d_6 (101 MHz)

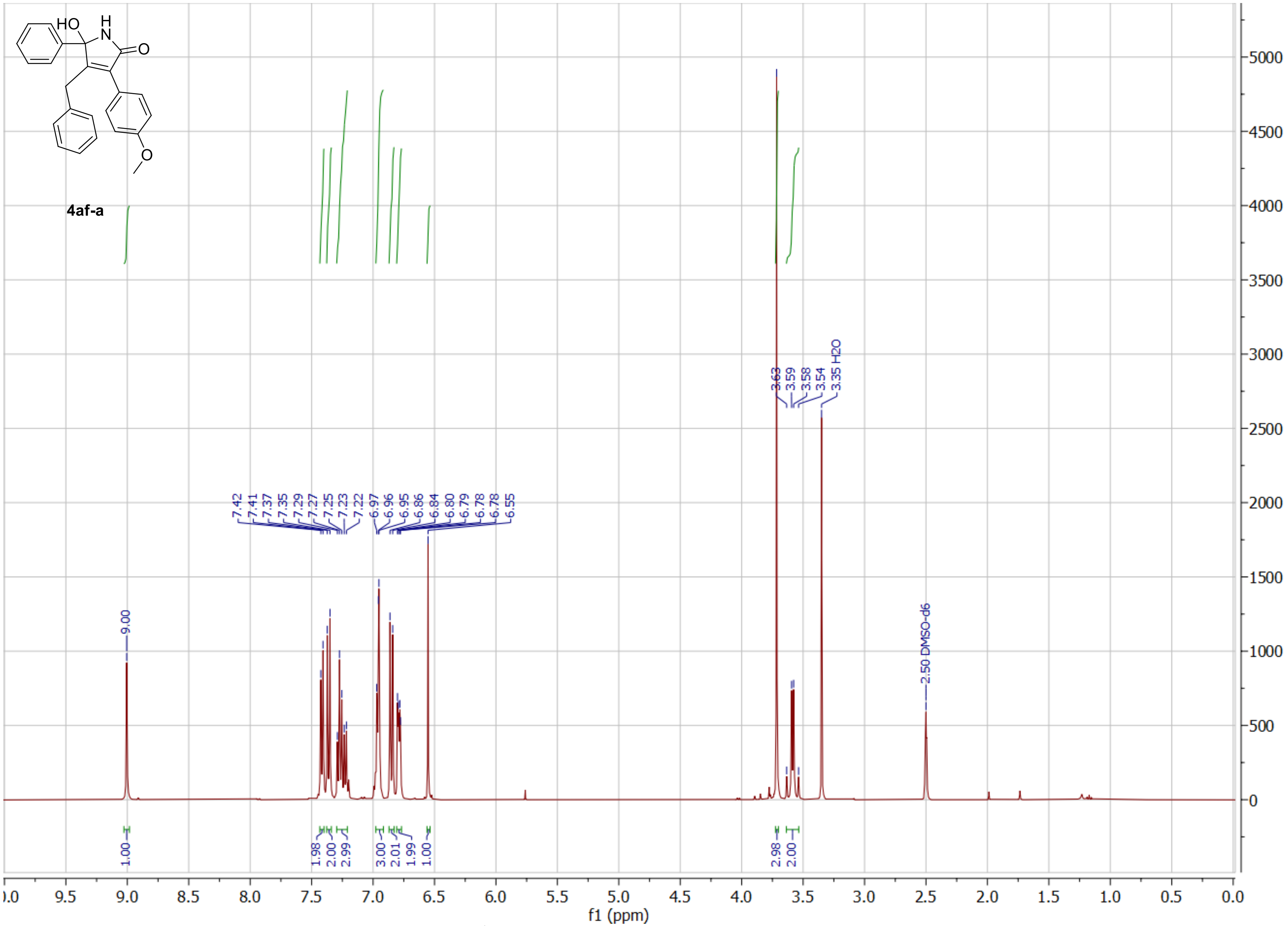

Figure S54. ^{1}H NMR spectrum of **4af-a** in DMSO-d_6 (400 MHz)

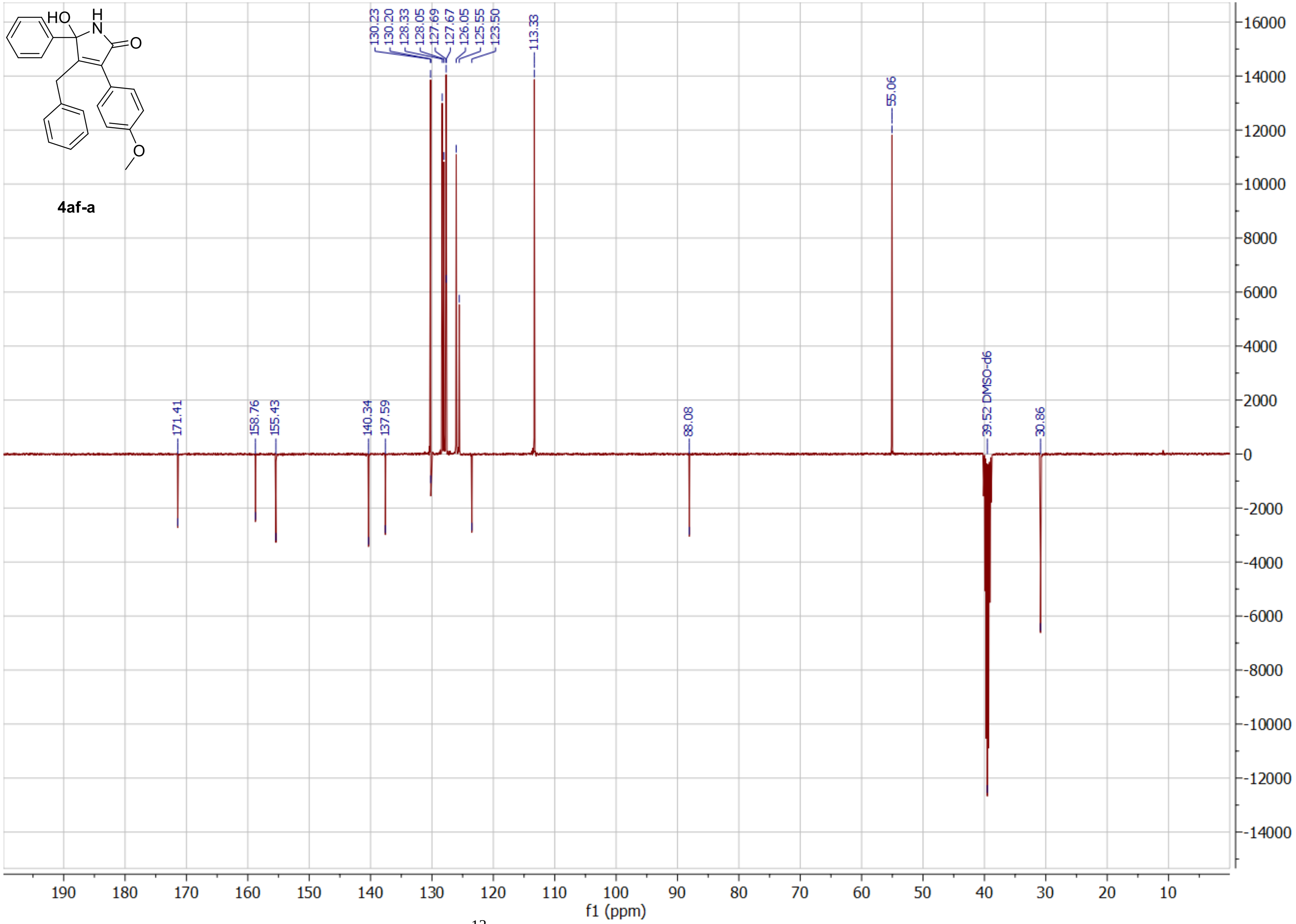

Figure S55. ^{13}C NMR spectrum of **4af-a** in DMSO-d_6 (101 MHz)

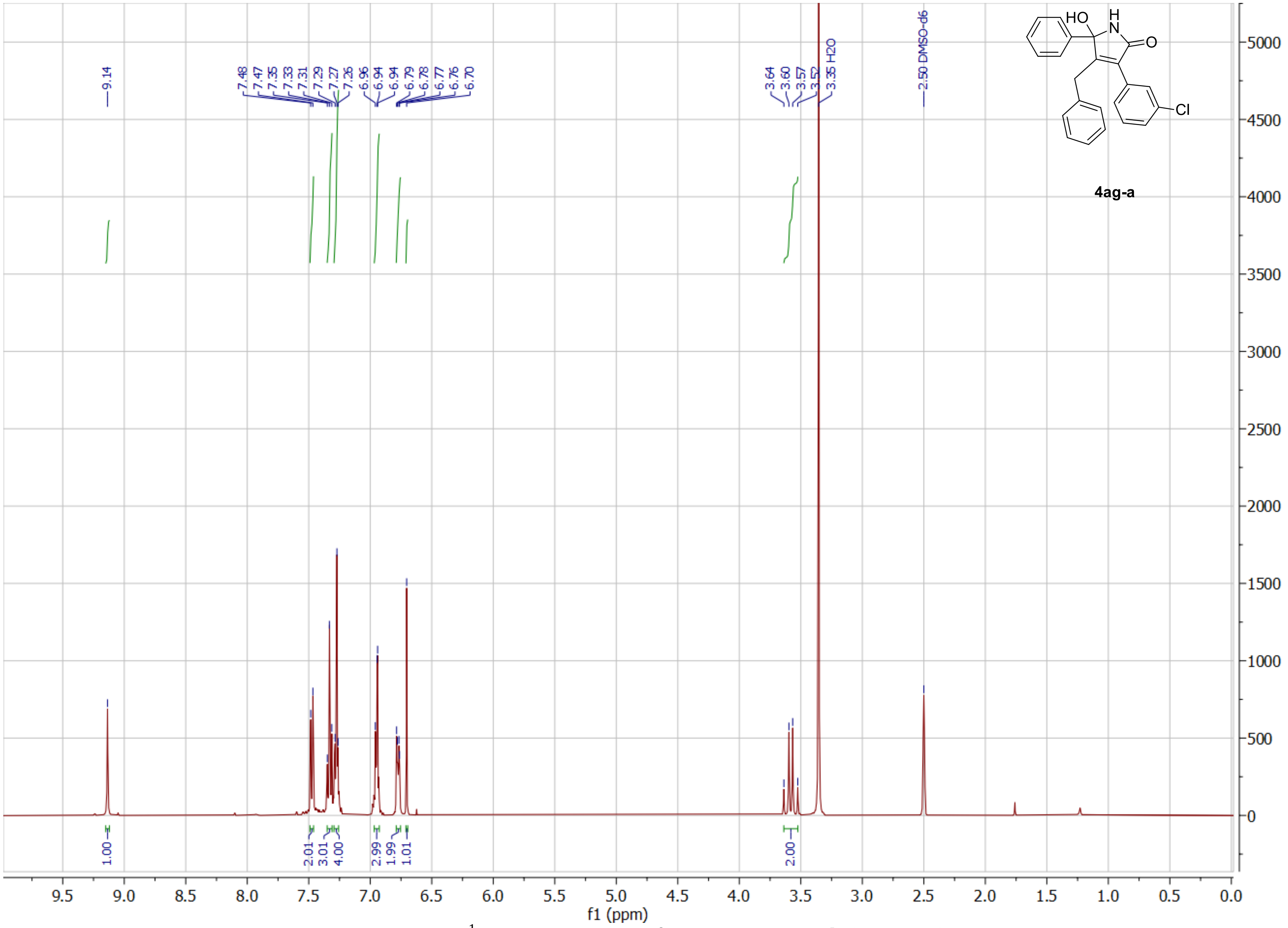

Figure S56. ^{1}H NMR spectrum of **4ag-a** in DMSO-d_6 (400 MHz)

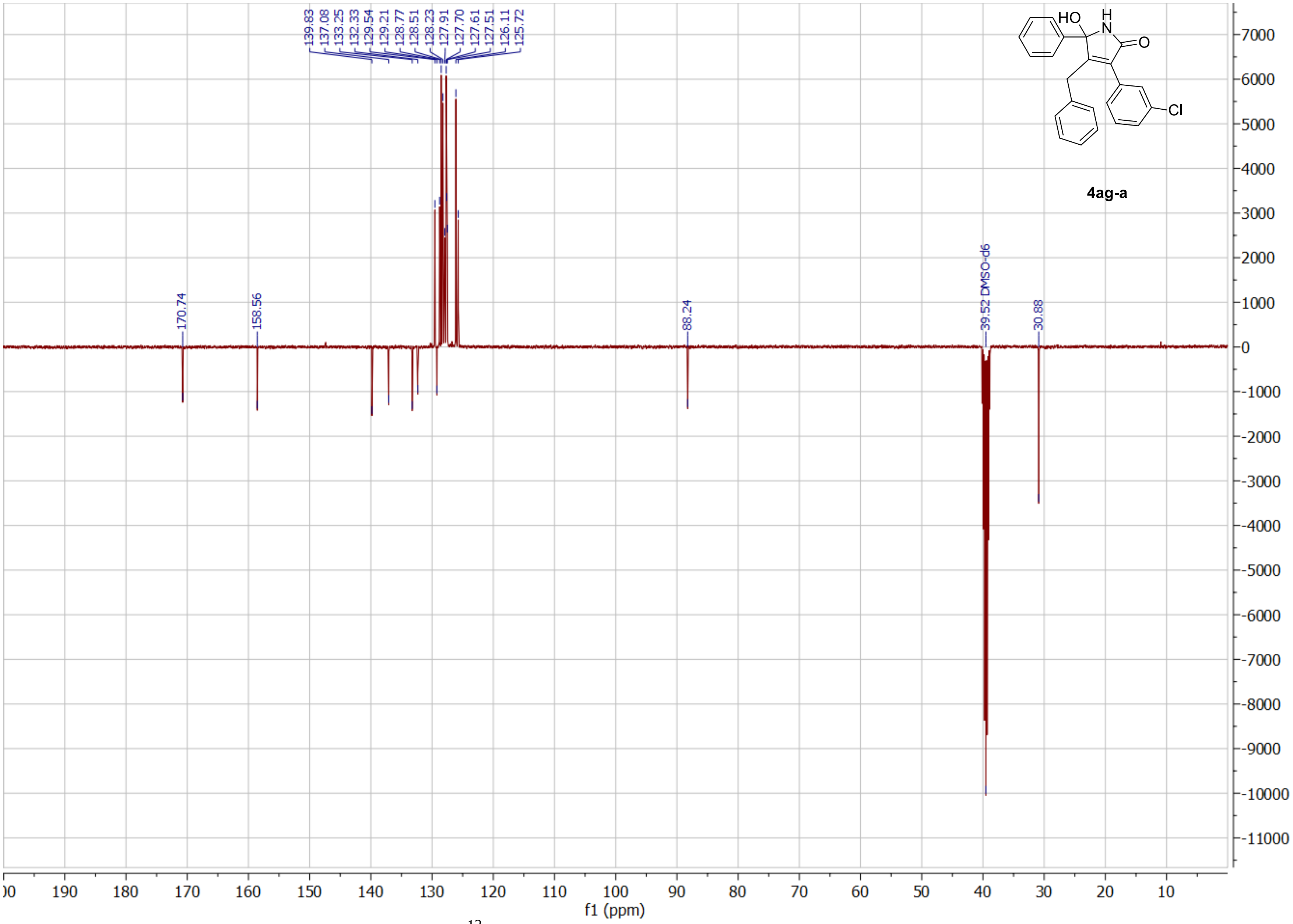

Figure S57. ^{13}C NMR spectrum of **4ag-a** in DMSO-d_6 (101 MHz)

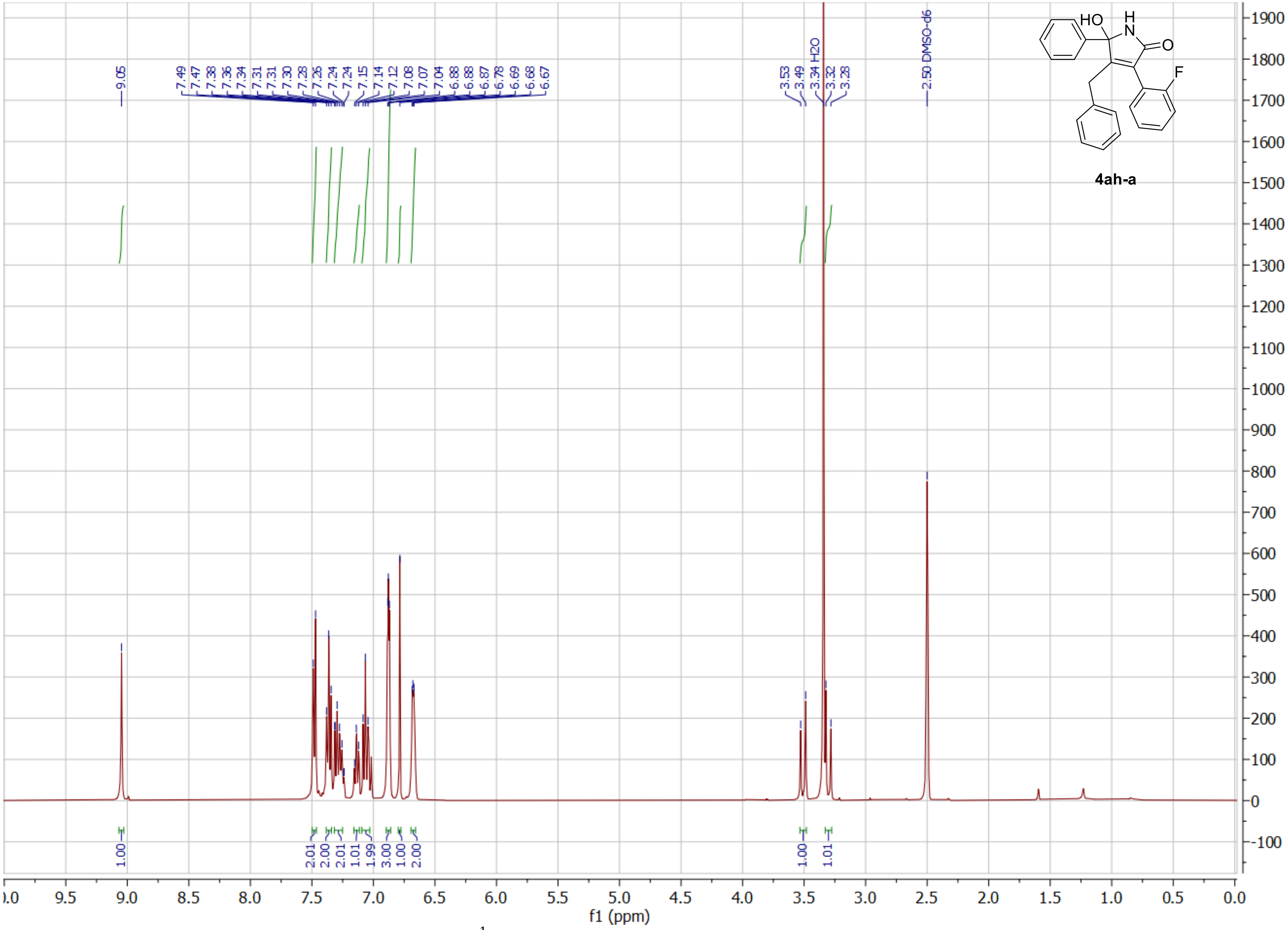

Figure S58. ^{1}H NMR spectrum of **4ah-a** in DMSO-d_6 (400 MHz)

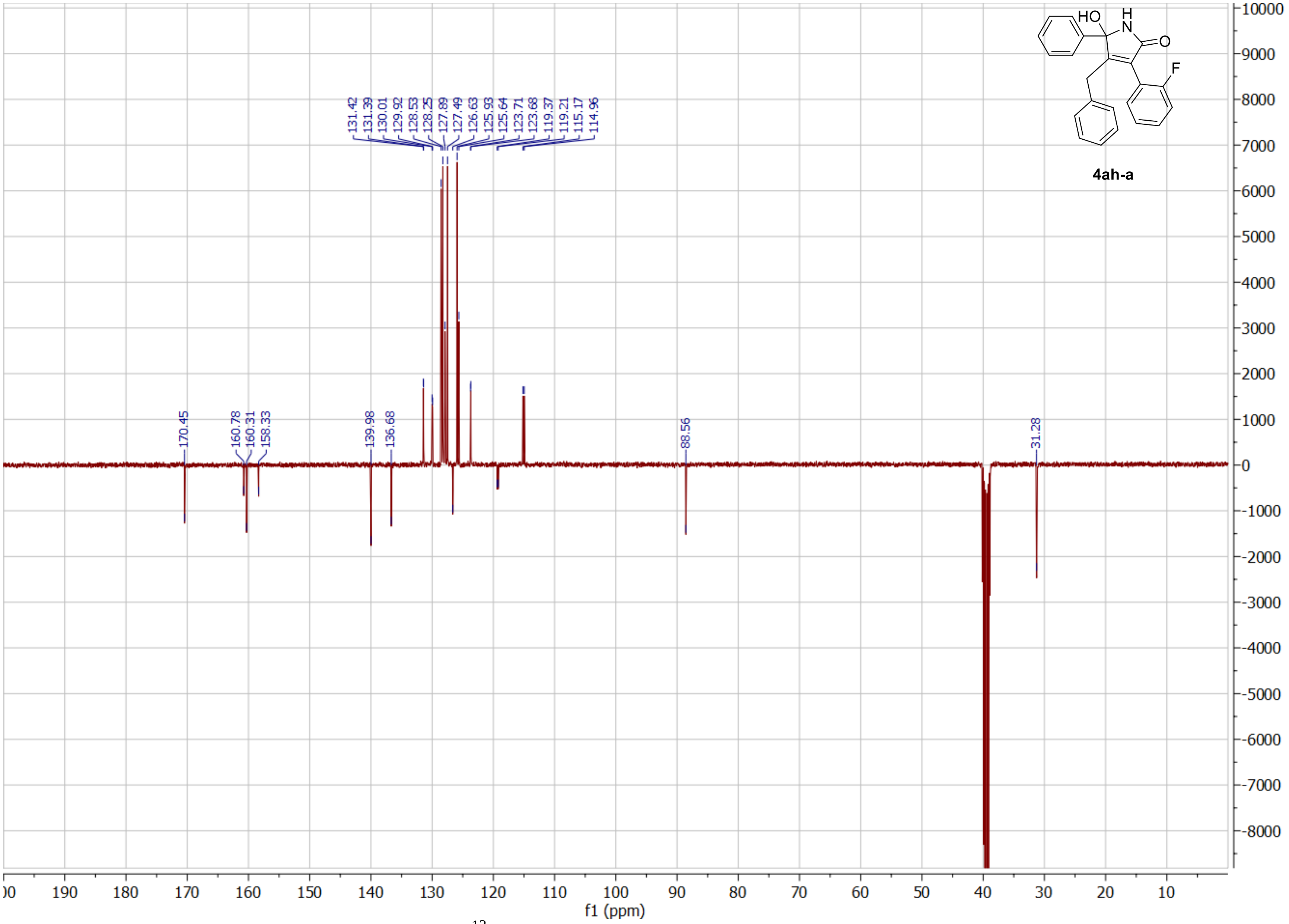

Figure S59. ^{13}C NMR spectrum of **4ah-a** in DMSO-d_6 (101 MHz)

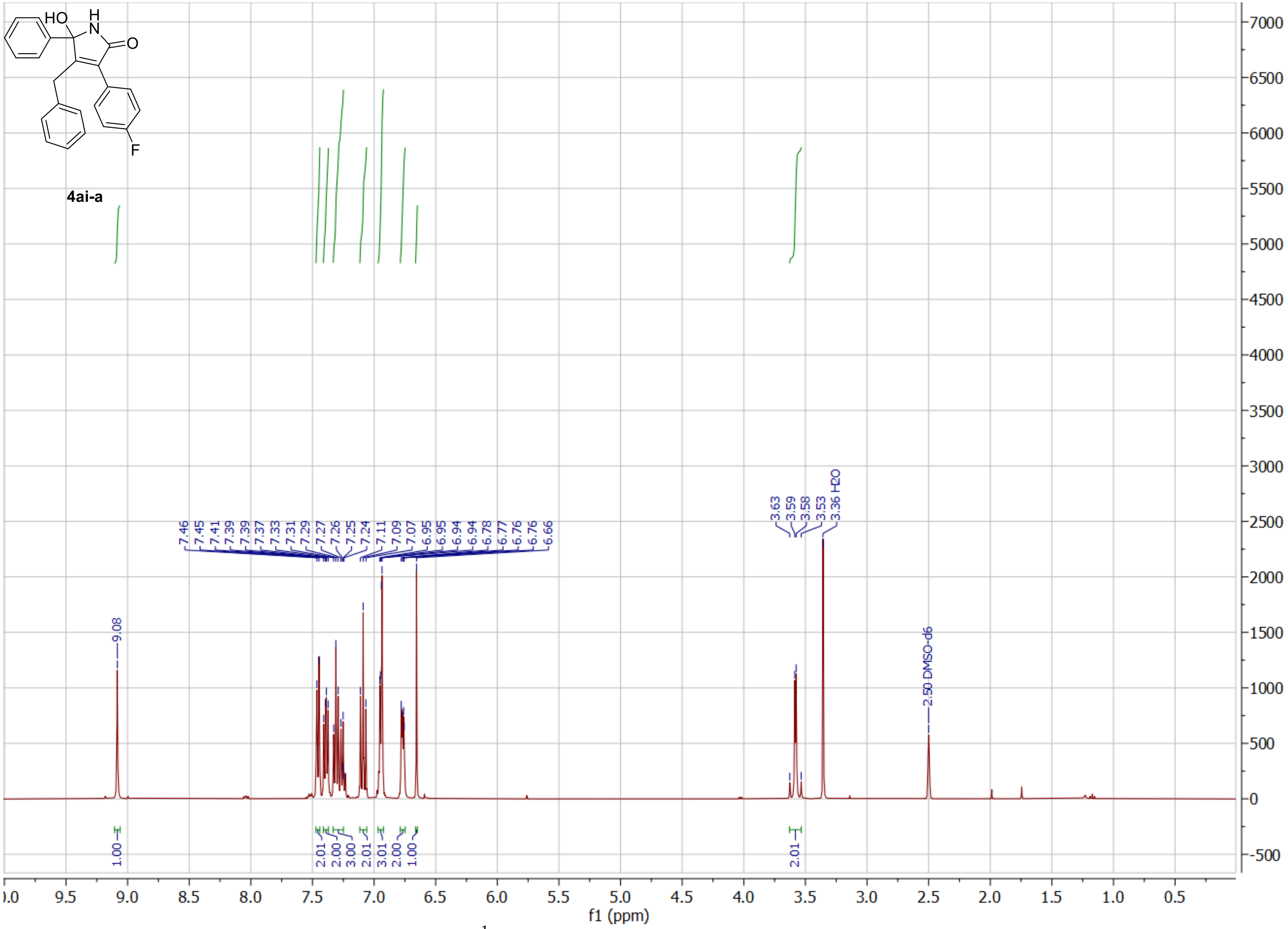

Figure S60. ^{1}H NMR spectrum of **4ai-a** in DMSO-d_6 (400 MHz)

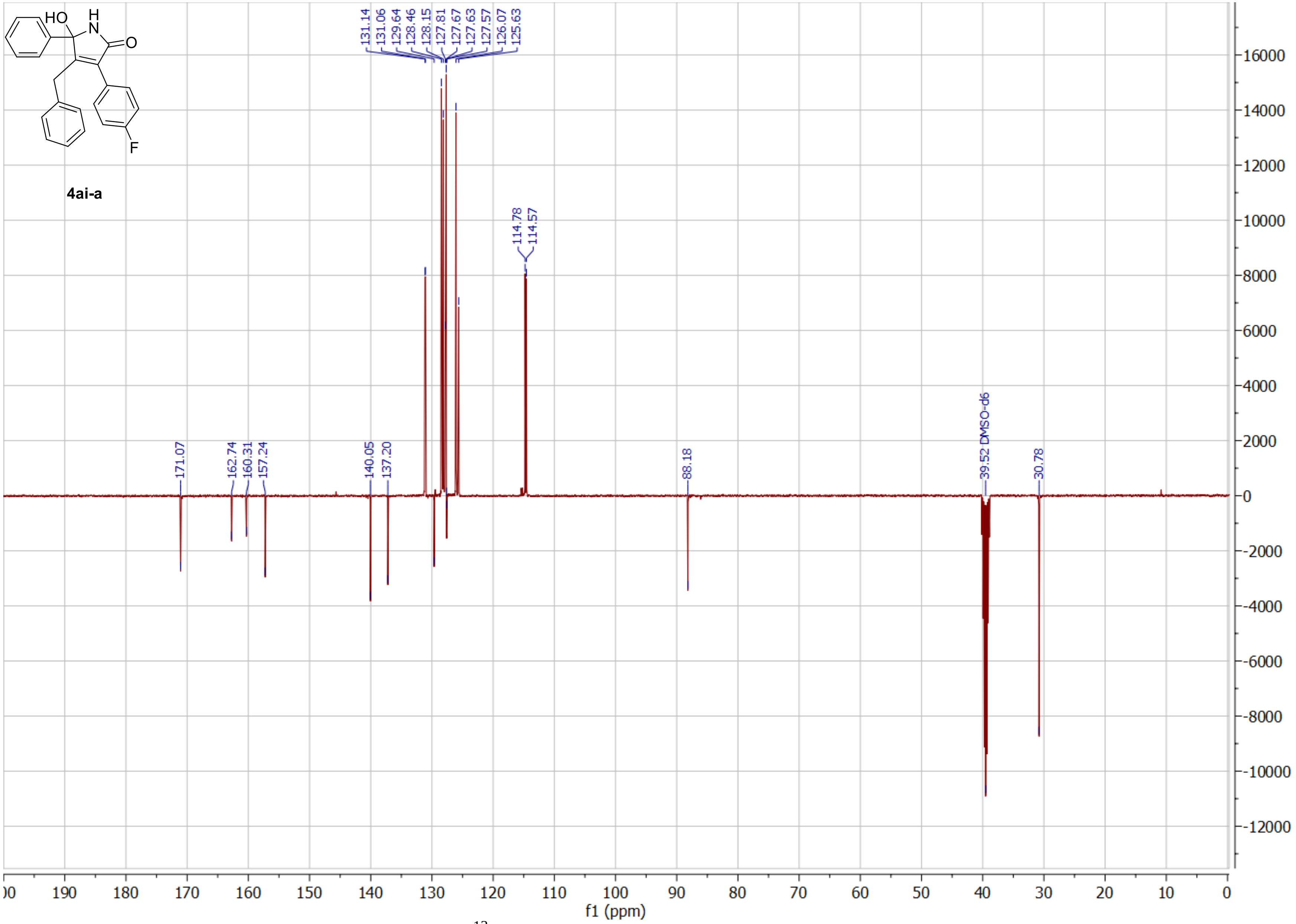

Figure S61. ^{13}C NMR spectrum of **4ai-a** in DMSO-d_6 (101 MHz)

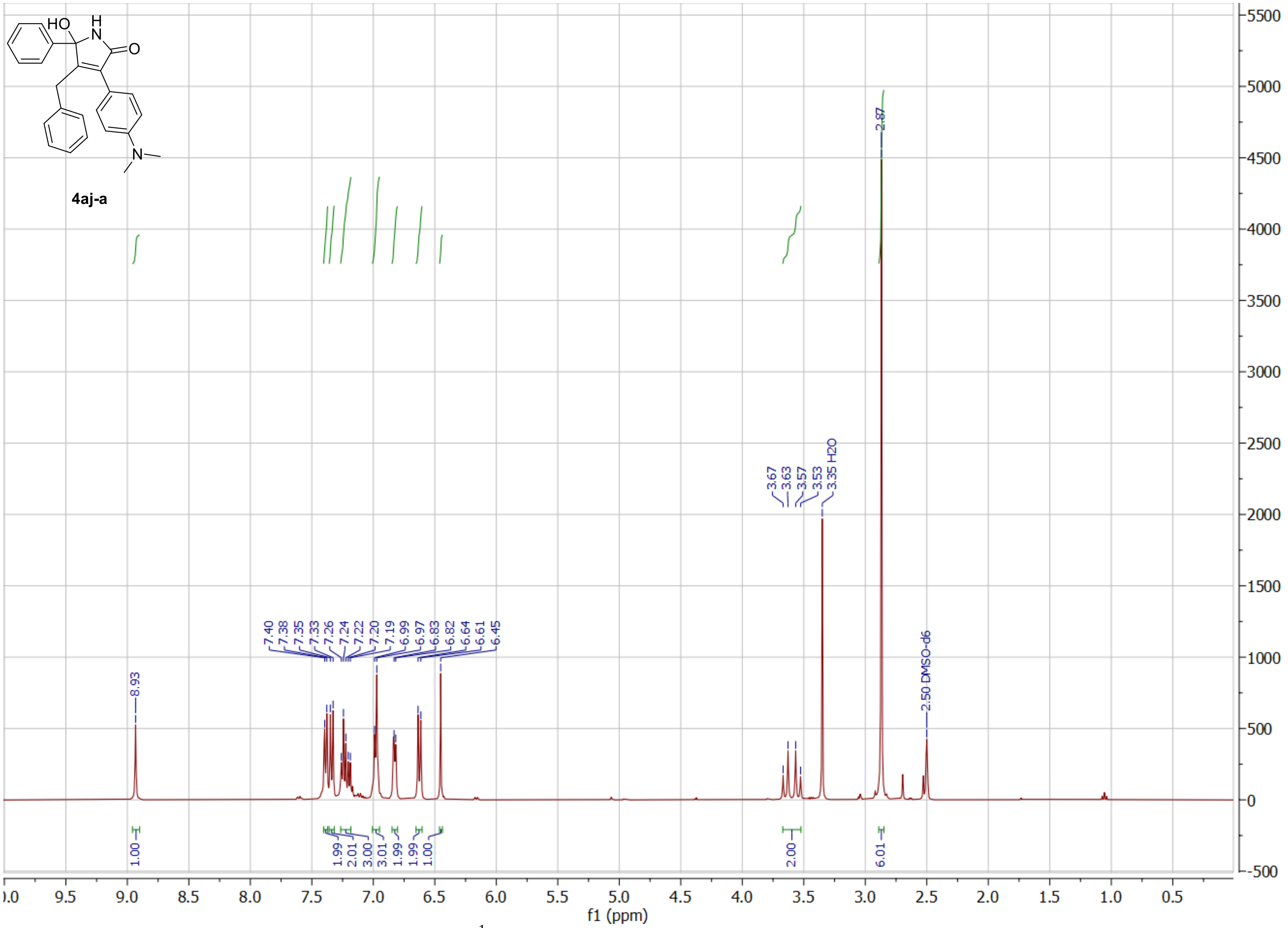

Figure S62. ^{1}H NMR spectrum of **4aj-a** in DMSO-d_6 (400 MHz)

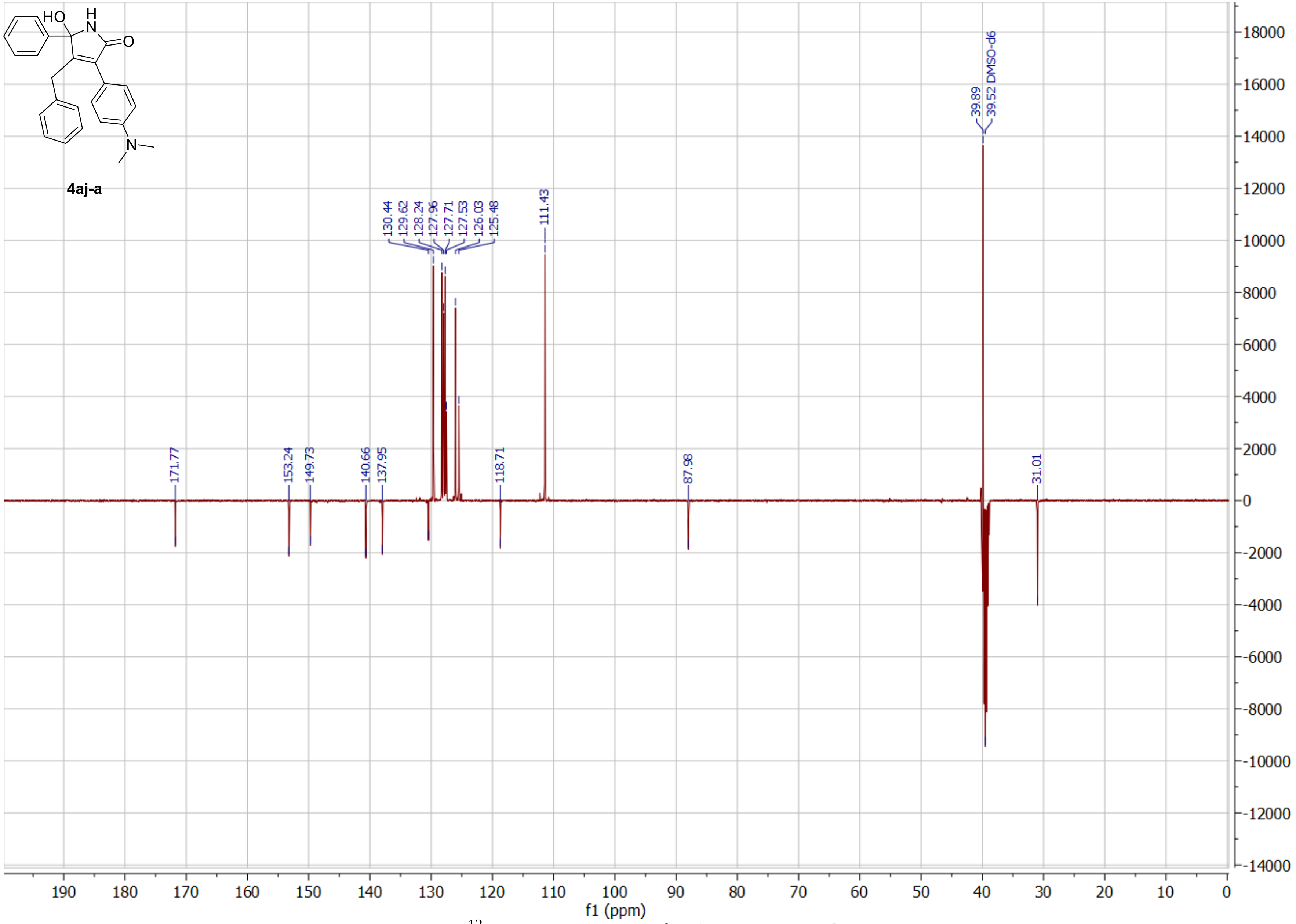

Figure S63. ^{13}C NMR spectrum of **4aj-a** in DMSO-d_6 (101 MHz)

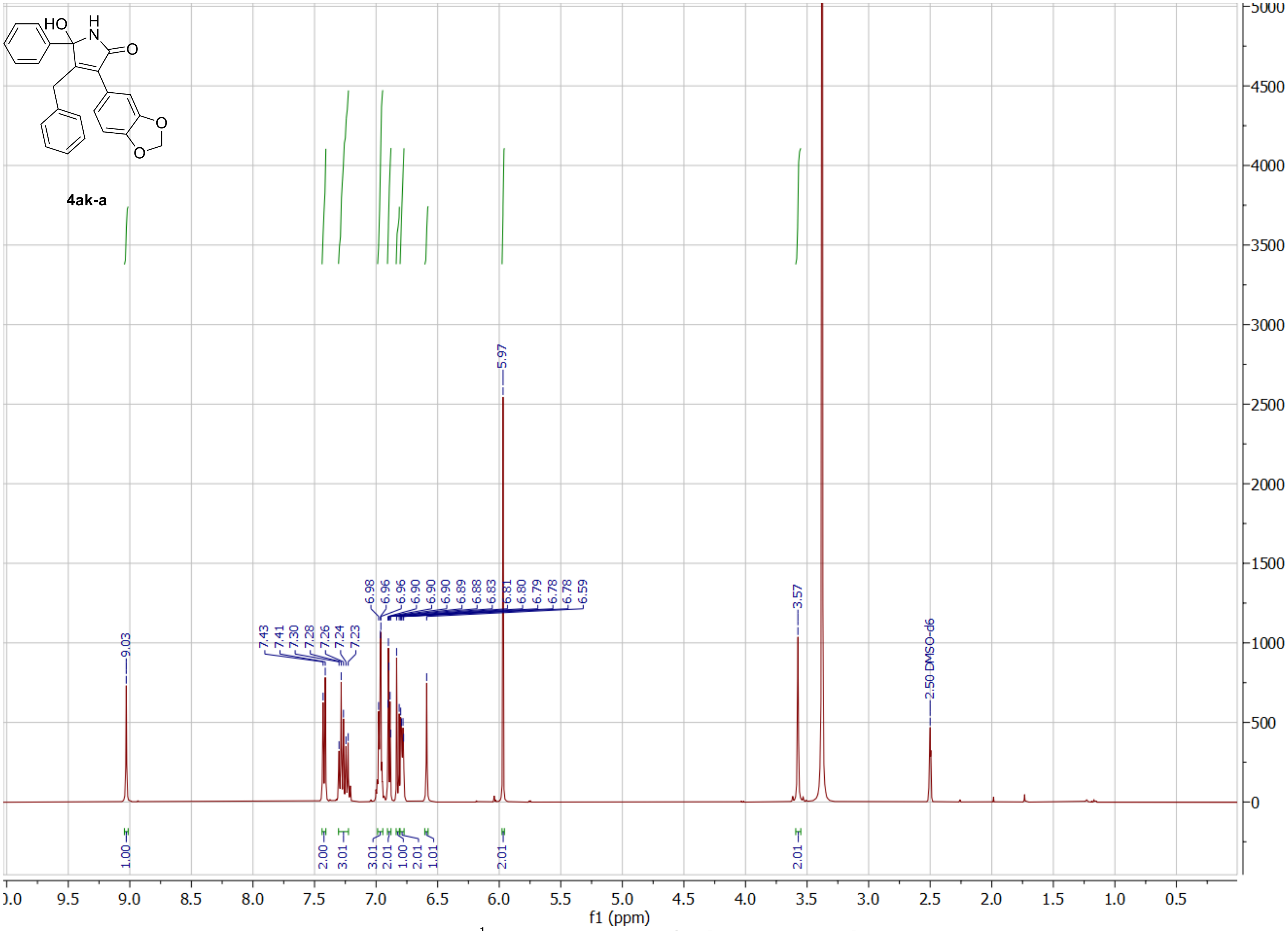

Figure S64. ^{1}H NMR spectrum of **4ak-a** in DMSO-d_6 (400 MHz)

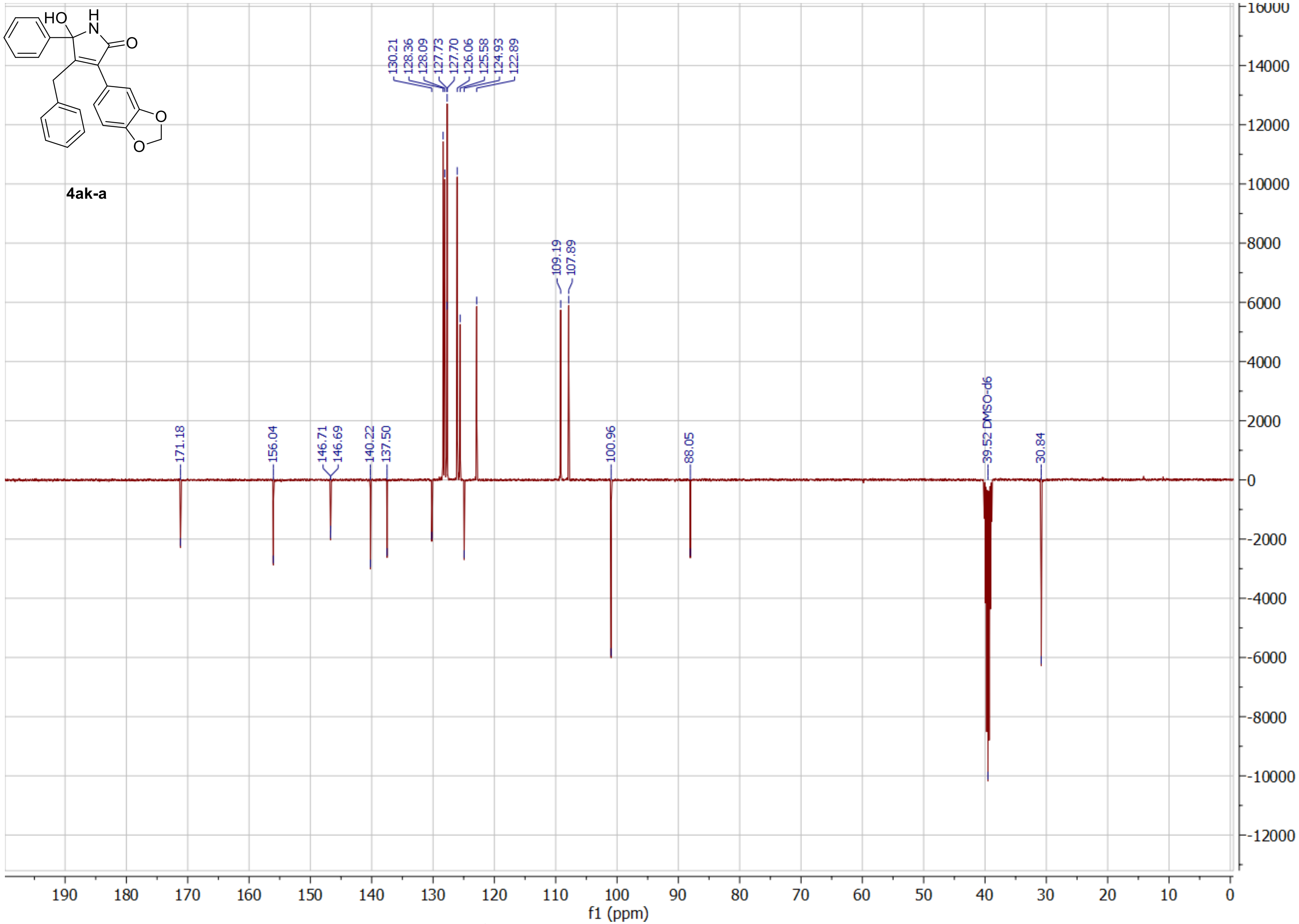

Figure S65. ^{13}C NMR spectrum of **4ak-a** in DMSO-d_6 (101 MHz)

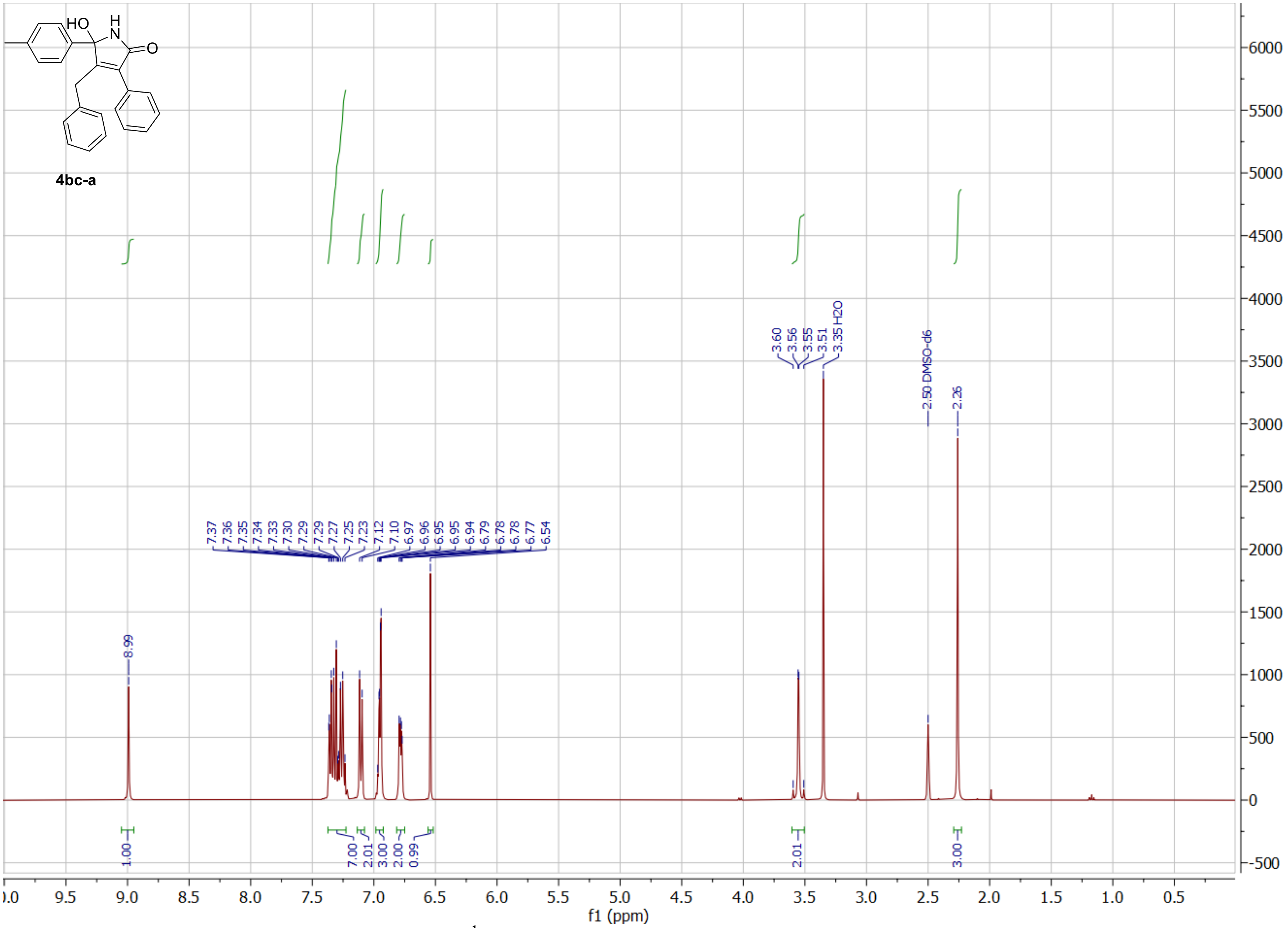

Figure S66. ^{1}H NMR spectrum of **4bc-a** in DMSO-d_6 (400 MHz)

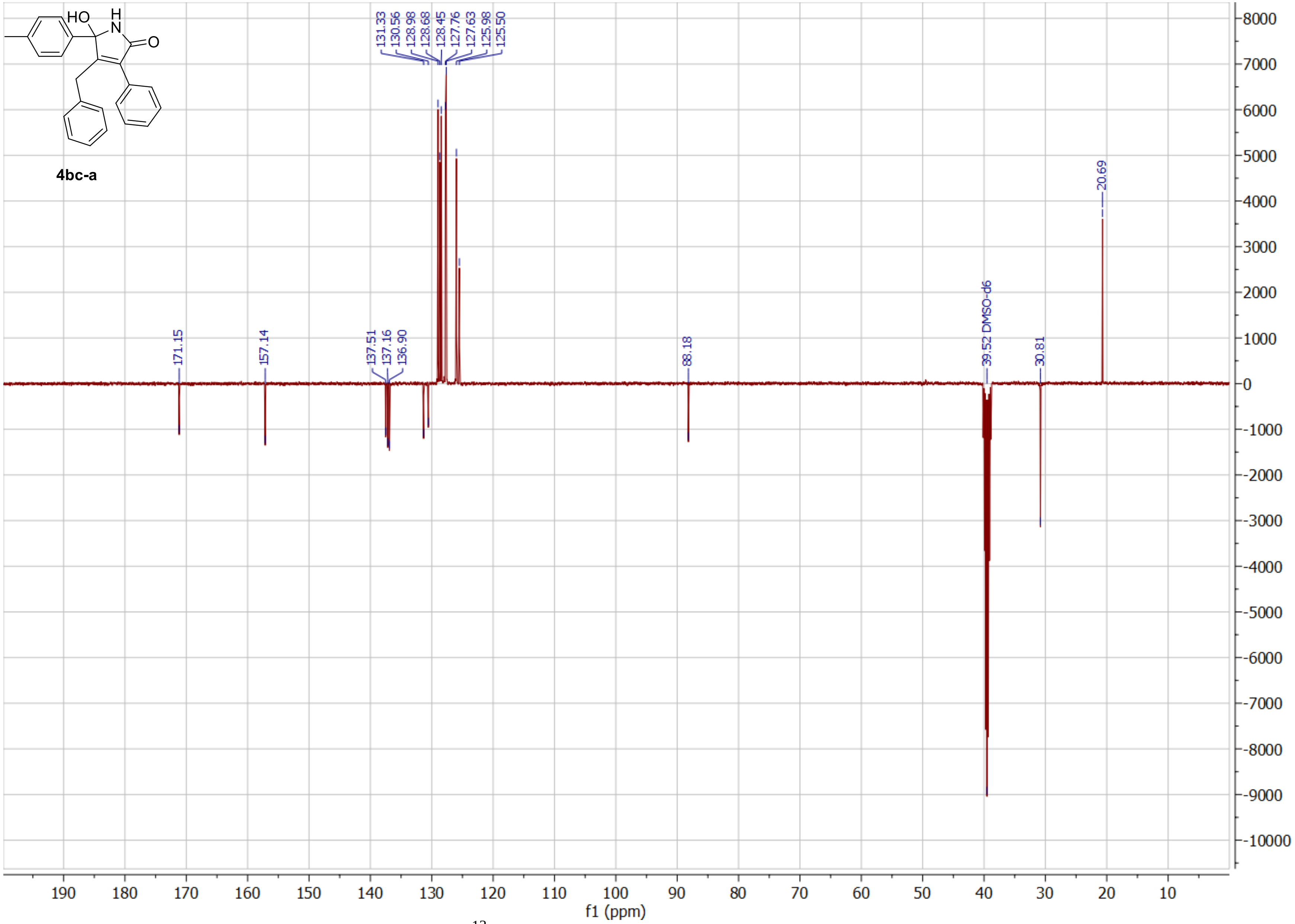

Figure S67. ^{13}C NMR spectrum of **4bc-a** in DMSO-d_6 (101 MHz)

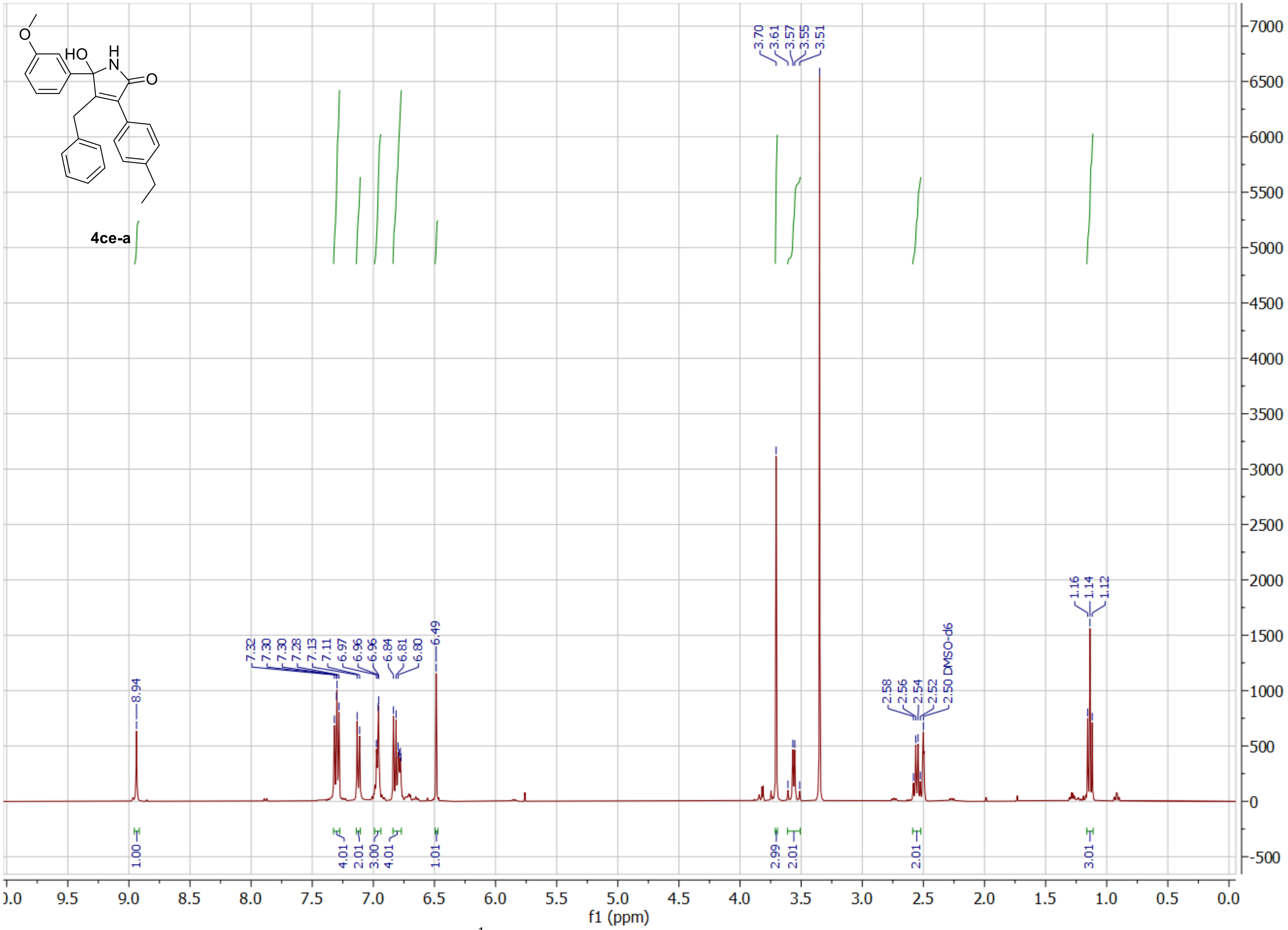

Figure S68. ^{1}H NMR spectrum of **4ce-a** in DMSO-d_6 (400 MHz)

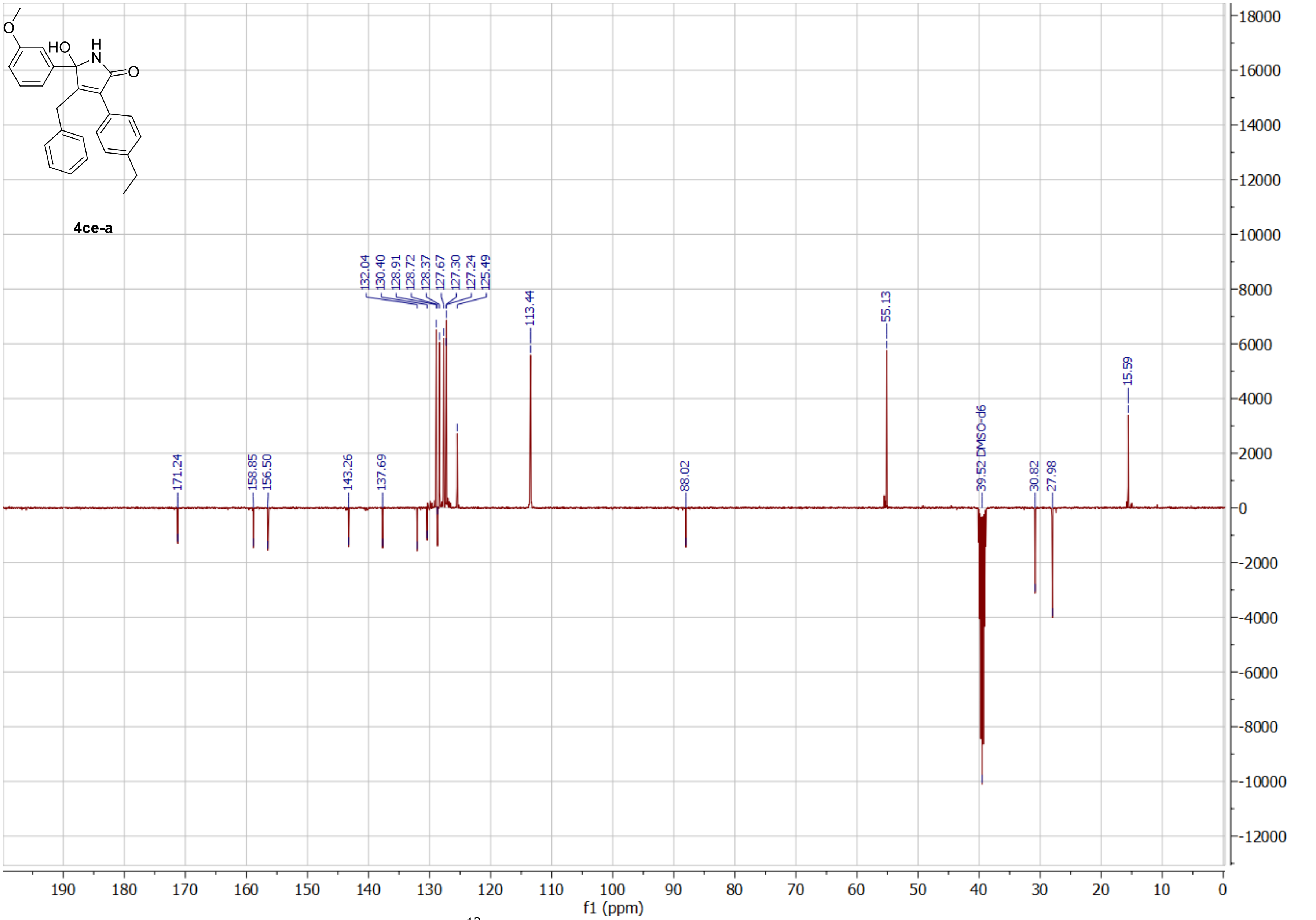

Figure S69. ^{13}C NMR spectrum of **4ce-a** in DMSO-d_6 (101 MHz)

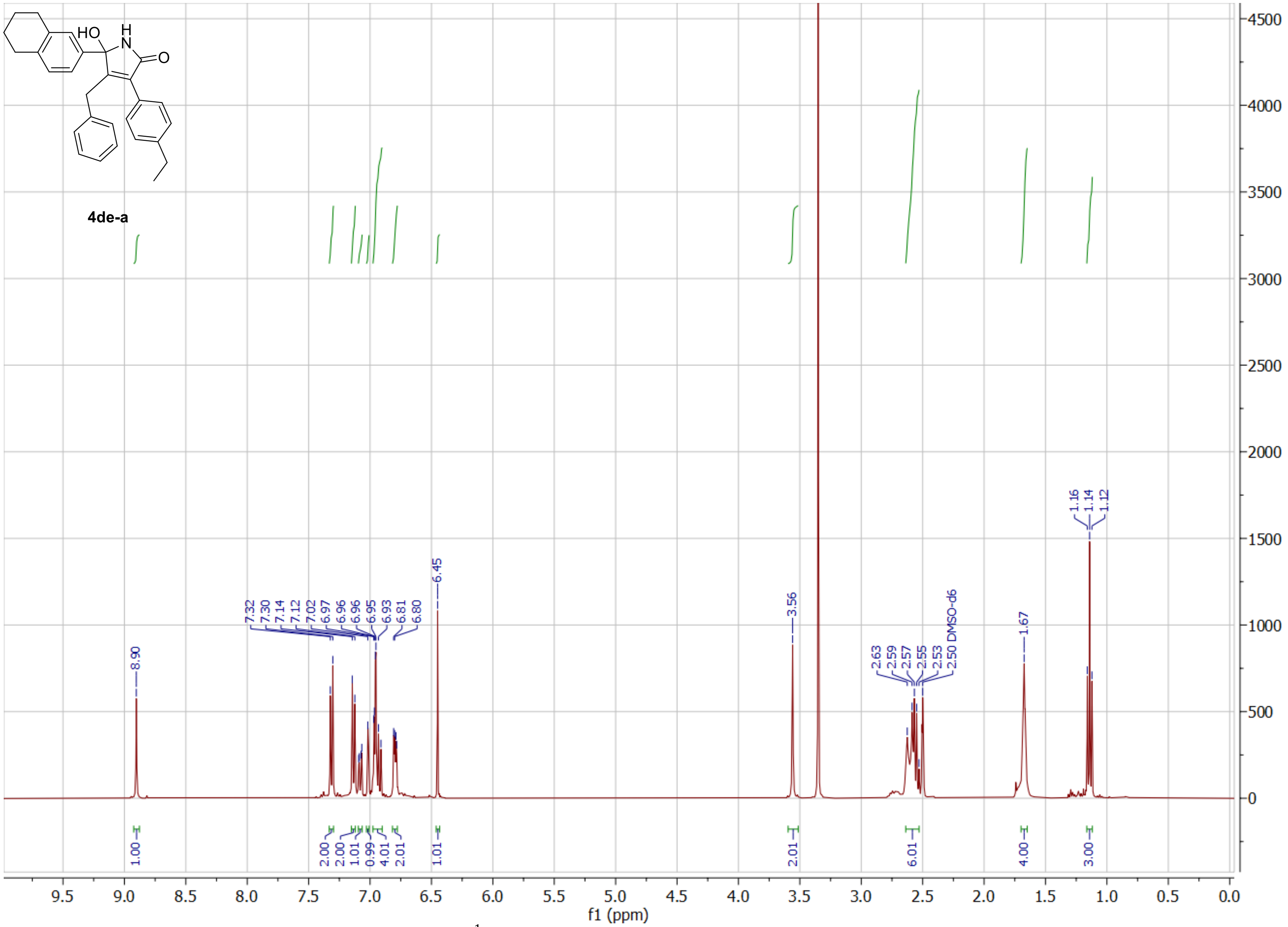

Figure S70. ^{1}H NMR spectrum of **4de-a** in DMSO-d_6 (400 MHz)

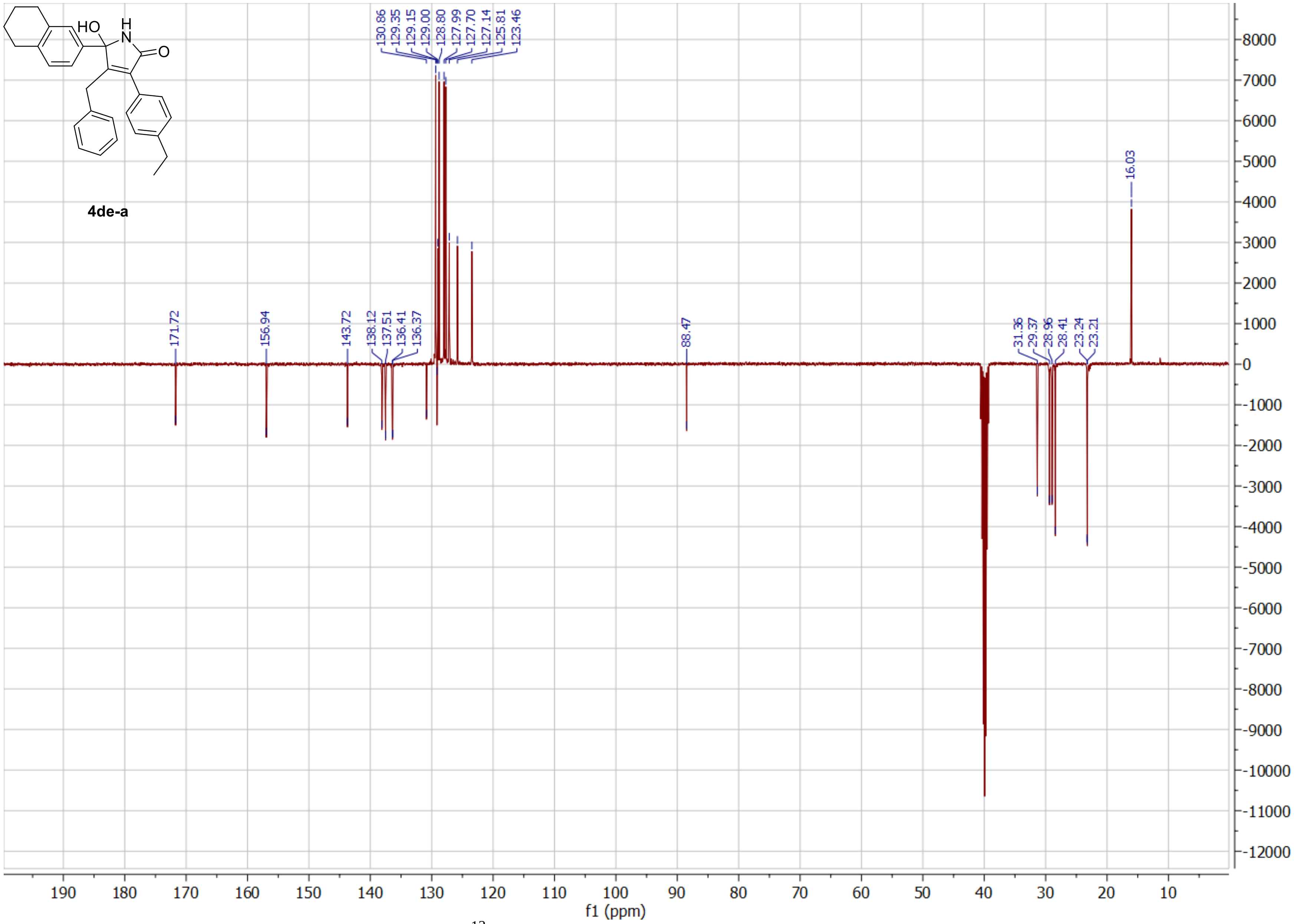

Figure S71. ^{13}C NMR spectrum of **4de-a** in DMSO-d_6 (101 MHz)

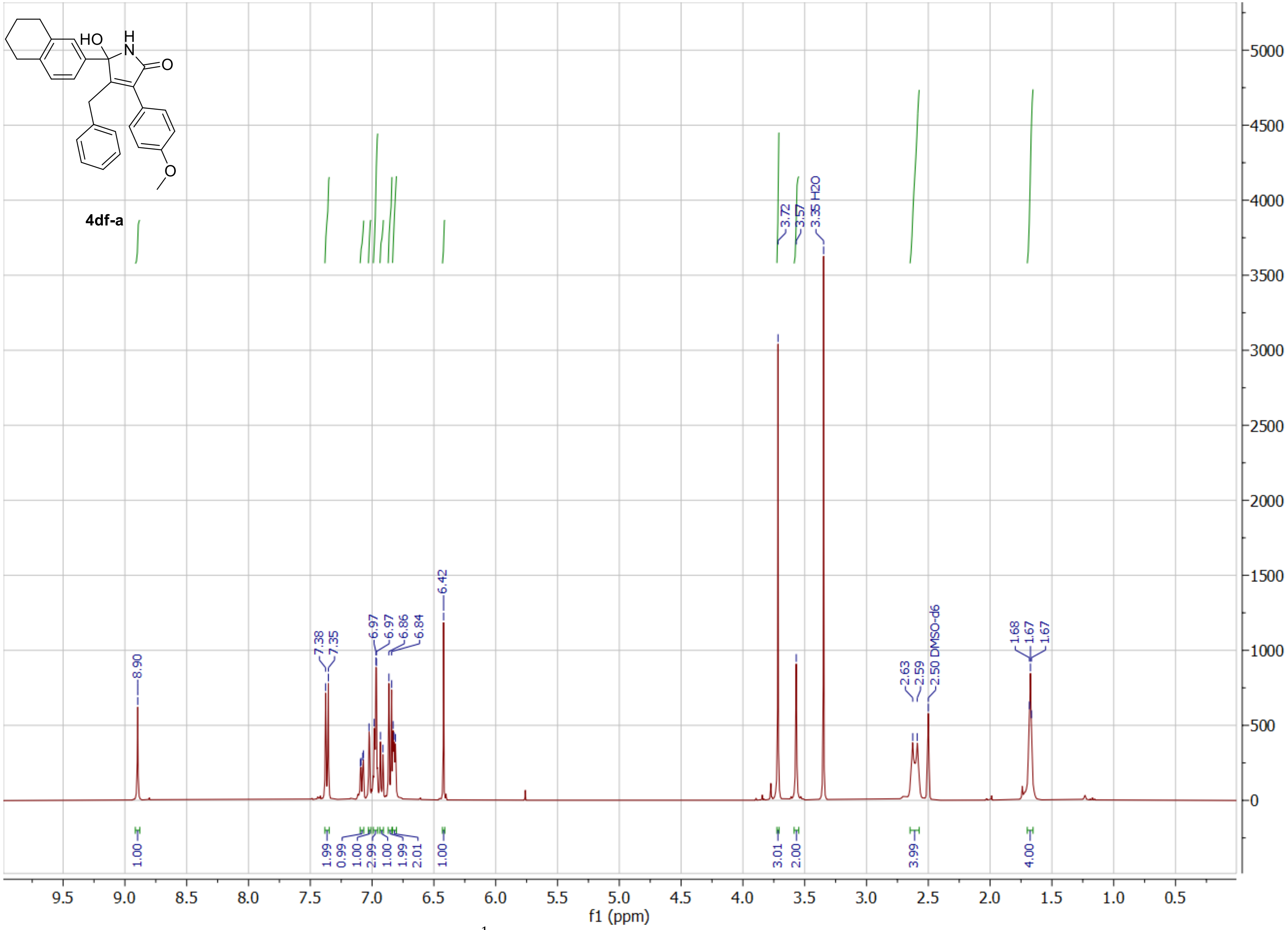

Figure S72. ^{1}H NMR spectrum of **4df-a** in DMSO-d_6 (400 MHz)

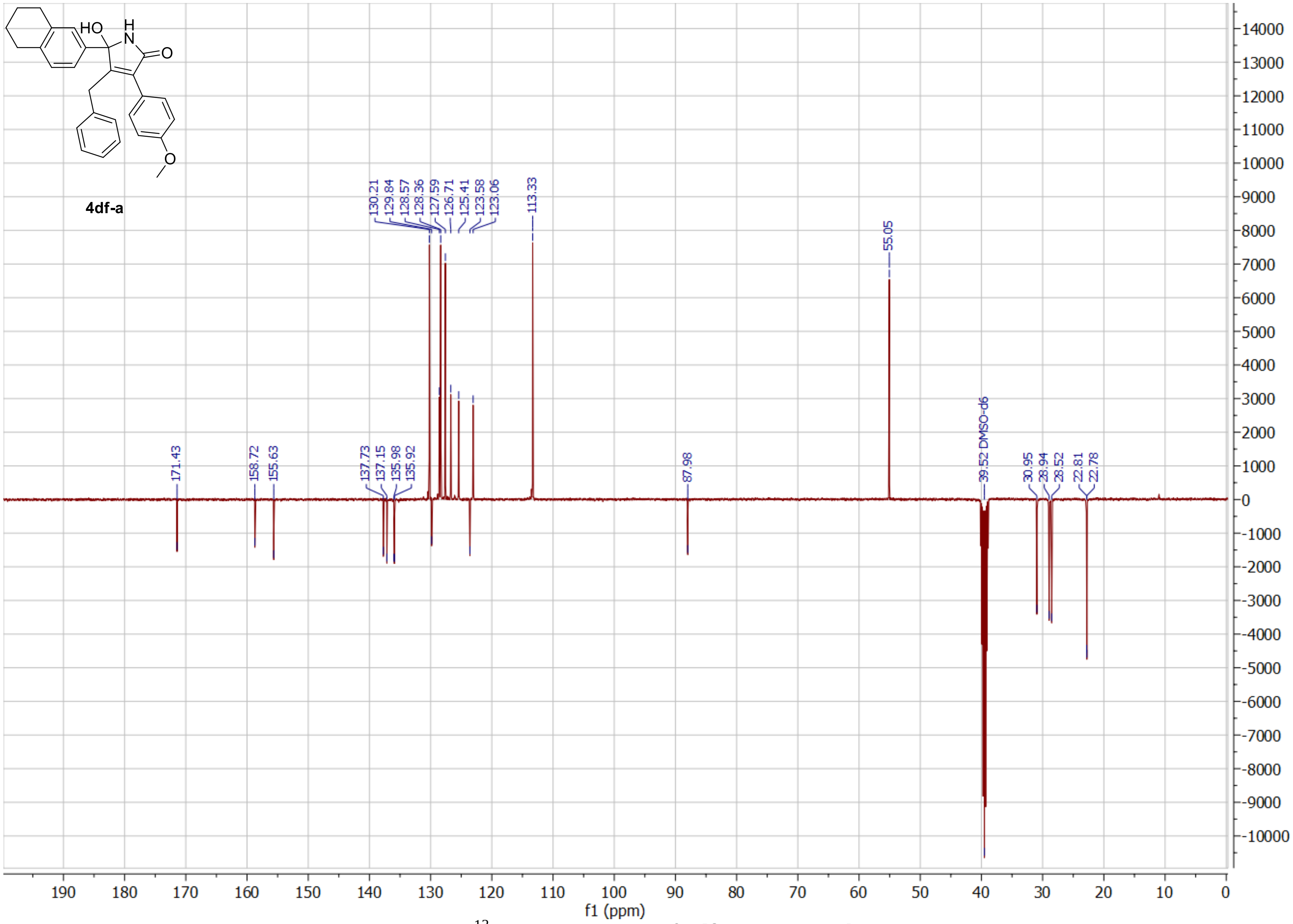

Figure S73. ^{13}C NMR spectrum of **4df-a** in DMSO-d_6 (101 MHz)
